# Comprehensive Molecular Docking and Molecular Dynamics Reveal Inhibitors of HER2 L755S, T798I, and T798M based on a Large Database of Curcumin Derivatives

**DOI:** 10.31557/APJCP.2026.27.1.265

**Published:** 2026-01-22

**Authors:** Mantiqa Syafa Duvadillan Gusrin, Yonika Arum Larasati, Rohmad Yudi Utomo

**Affiliations:** 1 *Graduate School of Master of Pharmaceutical Sciences, Faculty of Pharmacy, Universitas Gadjah Mada, Indonesia. *; 2 *Cancer Chemoprevention Research Center, Faculty of Pharmacy, Universitas Gadjah Mada, Sekip Utara, Sleman, Yogyakarta Indonesia.*; 3 *Translational Research Center in Oncohaematology, Department of Cell Physiology and Metabolism, Faculty of Medicine, University of Geneva, Swiss.*; 4 *Laboratory of Medicinal Chemistry, Department of Pharmaceutical Chemistry, Faculty of Pharmacy, Universitas Gadjah Mada, Sekip Utara, Sleman, Yogyakarta Indonesia. *

**Keywords:** HER2 L755S- HER2 T798I- HER2 T798M- Lapatinib- Curcumin

## Abstract

**Objective:**

This study presents a methodology employing virtual screening to identify curcumin derivatives with selective affinity for the HER2 mutations L755S, T798I, and T798M.

**Methods:**

Curcumin derivatives were retrieved from the ChEMBL database and filtered using KNIME. HER2 mutations were modeled in silico using MOE software with PDB ID 3RCD. Molecular docking and dynamics simulations were conducted to screen high-affinity compounds and evaluate binding interactions.

**Result:**

From 505 curcumin derivatives, the RDKit module implemented in KNIME successfully filtered 317 compounds. Subsequent molecular docking against wild-type HER2 identified 100 curcumin derivatives with low docking scores, among which the top 20 compounds exhibited better binding affinities than Lapatinib. Further molecular docking screening against the three HER2 mutations identified five lead compounds with the lowest docking scores. Molecular docking and molecular dynamics simulation revealed critical binding interactions with residues essential for kinase domain stability. Chemical structural analysis revealed key modifications, such as geranyl and tripeptide modifications. CHEMBL3758656 and CHEMBL3827366, two curcumin derivatives, demonstrated consistent binding across HER2 mutations and a favorable ADMET profile.

**Conclusion:**

This study successfully identified CHEMBL3758656 and CHEMBL3827366 as promising HER2 inhibitors through comprehensive virtual screening. Their high binding affinity against L755S, T798I, and T798M mutations and favorable ADME and toxicity properties underscore their potential as alternative therapeutics for HER2-positive breast cancer.

## Introduction

Breast cancer remains one of the most prevalent malignancies and a leading cause of cancer-related mortality worldwide. A significant subset of breast cancer cases, approximately 25%, is characterized by the overexpression of Human Epidermal Growth Factor Receptor 2 (HER2) proto-oncogene [1–3]. This overexpression is associated with a more aggressive tumor phenotype and correlates with poor patient prognosis [4–6]. While HER2-targeted therapies, such as the monoclonal antibody trastuzumab and tyrosine kinase inhibitor Lapatinib, have revolutionized treatment options and improved survival rates, many patients eventually develop resistance [7–9]. This resistance can arise from various intrinsic and acquired mechanisms, including mutations in the HER2 gene, which allow tumor cells to evade inhibition and continue proliferating despite treatment.

Lapatinib is a reversible dual tyrosine kinase inhibitor that targets HER2 and Epidermal Growth Factor Receptor (EGFR). It functions by binding to the ATP-binding site of the HER2 kinase domain, thereby inhibiting downstream signaling pathways critical for tumor growth and survival [10–12]. However, mutations in HER2, such as L755S, T798I, and T798M, have been identified as significant contributors to Lapatinib resistance [13–15]. These mutations alter the binding dynamics of Lapatinib, leading to diminished therapeutic efficacy and necessitating alternative treatment strategies. The L755S mutation occurs in 1% of all breast cancer cases. These mutations are significantly increased in metastatic tumors [14]. This HER2 alteration is a missense mutation that makes a reversible tyrosine kinase inhibitor (TKI) like lapatinib unable to interact with the hinge region in the HER2 kinase domain, and loses its efficacy [15]. Though the T798I and T798M are less commonly detected than the L755S mutation, these two mutations have been identified as an acquired gatekeeper mutation, which no longer responds to irreversible TKI neratinib. 

Curcumin, a bioactive compound derived from turmeric, has garnered attention for its potential anti-cancer properties, particularly against HER2-positive breast cancer [16–18]. Curcumin and its derivatives exert an inhibitory effect on the HER2 signaling pathway and could overcome resistance in several types of cancer cells [19–21]. The ability of curcumin and its derivatives to modulate various molecular targets makes them a promising candidate for enhancing the effectiveness of existing HER2-targeted therapies.

**Figure 1:**
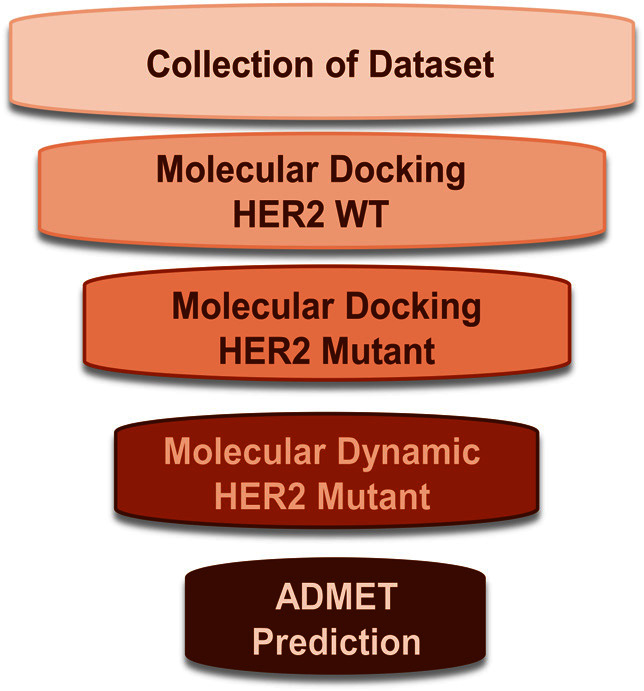
Workflow of Virtual Screening in This Study

This study proposes an approach utilizing virtual screening techniques to identify curcumin derivatives that specifically target the HER2 mutations L755S, T798I, and T798M using our workflow (Figure 1). By employing extensive molecular docking, molecular dynamic simulations, and ADMET prediction, we could elucidate the binding interactions between these derivatives and HER2 mutations. This research also informed the chemical structure requirements for developing curcumin derivatives as HER2 inhibitors, both their wild-type and mutated versions.

## Materials and Methods

### Preparation of Dataset containing Curcumin Derivatives from ChEMBL Database

The curcumin derivatives were retrieved from the ChEMBL database using the advanced search module, inputting the SMILES query O=C(CC(C=C)=O)C=C. The list of compounds was then downloaded as a .csv file for further filtering based on the Lipinski rule of five using the RDKit module in the KNIME workflow and Principal Moment of Inertia (PMI) analysis. 

### In Silico Mutation of HER2 in MOE

The default protocol for in silico mutation in MOE was used without further modification. As the model of HER2 wild-type (WT), this study employed PDB ID 3RCD, considering the location of the ATP-binding site, which is relevant to the location of the mutation. After single-point amino acid modifications, the crystal structure models were subjected to energy minimization and side-chain optimization algorithms to ensure the mutated structure achieves a stable conformation. The quality of models was then analyzed using a Ramachandran plot based on the Phi-Psi geometry.

### Molecular Docking Simulation

Molecular docking using MOE was performed on the HER2 protein, utilizing PDB ID 3RCD, with TAK-285 as a known inhibitor in the kinase domain [22]. The default protocol was conducted based on the previous report by Lestari et al. [23]. The HER2 structure was prepared by removing unnecessary water molecules while retaining the co-crystallized inhibitor, adding hydrogen, and optimizing the structure using the Amber10 force field. The binding site for simulation was defined based on the active pocket of TAK-285 as the native ligand in the kinase domain consisting of several amino acids such as Leu726, Gly727, Ser728, Gly729, Val734, Ala751, Ile752, Lys753, Ser783, Arg784, Leu785, Leu796, Thr798, Gln799, Leu800, Met801, Gly804, Cys805, Leu852, Thr862, Asp863, Leu852, Phe864, and Phe1004. Docking simulations were performed using the Triangle Matcher algorithm for pose generation, both for flexible ligands and rigid proteins. Initial scoring was conducted with the London dG scoring function, which estimated the free energy of binding using the following equation based on previous report from Labute [24], where *c* represented the average gain or loss of rotational and translational entropy; *E*_flex_ accounted for the energy penalty due to the loss of flexibility of the ligand (calculated from ligand topology only); *f*_HB_ and *f*_M _were geometric factors (ranging from 0 to 1) for hydrogen


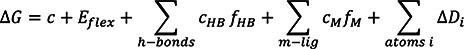


 bonds and metal coordination imperfections, respectively; *c*_HB_ and *c*_M_ were the ideal energies for hydrogen bond and metal coordination; and *Di* denoted the desolvation energy of atom i. Desolvation energy differences were calculated according to the formula below,


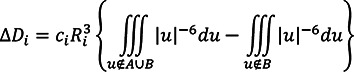


where *A* and *B* represented the volume of the protein and/or ligand, with atom i belonging to volume *B*; *R*_i_ denoted the solvation radius of atom i, defined as the OPLS-AA van der Waals sigma parameter plus 0.5 Å; and *c*_i_ was the desolvation coefficient specific to of atom i. The coefficients {*c,c*_HB_*,c*_M_*,c*_i_} were derived by fitting data from approximately 400 X-ray crystal structures of protein-ligand complexes with known experimental *pK*_i_ values. For the assignment of the *c*_i_ coefficients, atoms were classified into approximately a dozen atom types. The triple integrals required for desolvation energy calculations were approximated using Generalized Born integral formulations.

Further refinement of binding energy estimation was performed using GBVI/WSA dG as the rescoring procedure to estimate the free energy of binding of the ligand from a given pose based on forcefield scoring function using the following equation based on Naïm et al. [25],

where, *c* represented entropic contributions; *α* and *β* were force field-dependent constants 


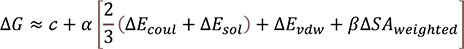


obtained during training; *E*_coul_ was the coulombic electrostatic term, calculated using partial charges with a constant dielectric of ϵ=1; *E*_sol_ was the electrostatic solvation energy calculated using the GB/VI solvation model; *E*_vdw_ represented the van der Waals interactions; and *SA*_weighted_ denoted the weighted solvent-accessible surface area. The evaluation of the redocking protocol was based on the RMSD value, with an acceptable score of ≤ 2Å. Top-ranked poses after virtual screening were analyzed for key interactions with the kinase domain residues.

### Molecular Dynamics Simulation

The molecular dynamics (MD) simulation protocol was performed according to established procedures previously reported by Hermawan et al. [26] . The MD simulations were performed using NAMD 2.14 and visualized with VMD 1.9.4 [27, 28]. Protein and ligand parametrizations were generated with CHARMM36 and CGenFF, which were available on the CHARMM-GUI web server. To prepare for solvation and neutralization, a cubic water box with 20 Å padding was added, followed by the addition of K+ and Cl- ions. The complex underwent a one ns minimization step, followed by a 10 ns simulation, which was carried out under NPT ensemble conditions (pressure 1 atm, temperature 303K) to complete the MD simulation. The MD results were analyzed using VMD, which employed simulation trajectories to calculate the root-mean-square deviation (RMSD), root-mean-square fluctuation (RMSF), radius of gyration (Rg), and solvent-accessible surface area (SASA). 

### MM-GBSA Calculation

The binding free energies of curcumin derivatives and the HER2 protein were estimated using the Molecular Generalized Born Surface Area (MM-GBSA) method, as implemented in MolAICal. This open-access software platform integrated deep learning models with a fragment growth algorithm (https://molaical.github.io). MM-GBSA calculations were conducted based on MD simulation trajectories to account for the dynamic nature of ligand-receptor interactions. The binding free energy (ΔGbind) was determined using the following equation:

${∆G}_{\mathrm{bind}}=G_{\mathrm{complex}}-(G_{\mathrm{complex}}+G_{\mathrm{lingand}})$

Where G_complex_, G_protein_, and G_ligand_ represented the total free energies of the protein-ligand complex, the unbound protein, and the free ligand, respectively. This methodology incorporated solvation effects and electrostatic interactions, yielding a biologically relevant assessment of binding stability.

### Prediction of ADMET Profile

Since the interaction of compounds with protein targets was partially determined by their bioavailability profile, we utilized pkCSM webserver (http://biosig.unimelb.edu.au/pkcsm/prediction) to predict the ADMET properties of curcumin derivatives [29]. The SMILES representations of the curcumin derivatives were input as queries, generating predictive data such as logP, water solubility, Caco-2 permeability, intestinal absorption, skin permeability, VDss, total clearance, max. human tolerated dose, oral rat acute toxicity, oral rat chronic toxicity.

## Results

### Collection of Curcumin Database and Molecular Docking Screening of HER2 wild type

A dataset of reported curcumin derivatives was systematically retrieved from ChEMBL using a SMILES-based query to explore their structural diversity and pharmacology potential. Given that curcumin derivatives had well-documented bioactivities and versatile chemical frameworks, modifying their structure could enhance binding affinities [30–32]. To refine the dataset, we applied a KNIME workflow that enabled automated filtering and molecular property analysis (Supplementary Figure 1A). Using the RDKit module, KNIME analysis filtered the 505 curcumin derivatives based on the Lipinski rule of five, selecting 317 curcumin derivatives with favorable drug-like characteristics (Supplementary Table 1). To assess molecular shape diversity, we performed the PMI analysis, which played a key role in understanding the chemical structure recognition. The PMI profile revealed that most derivatives retained a rod scaffold, while some displayed a disc structure (Supplementary Figure 1B). The curated set of curcumin derivatives was subsequently utilized for further computational analysis.

The virtual screening was initiated by performing a redocking study and molecular docking screening against the HER2 wild-type. This study utilized PDB ID 3RCD, considering the presence of known inhibitor TAK-285, which bond to the ATP-binding site through a combination of hydrophobic contacts and direct hydrogen bonds [22]. Our redocking analysis, performed using the default docking protocol in MOE, generated a docking score of -9.8297 kcal/mol with an RMSD of 0.643 Å, indicating that our procedure was acceptable for virtual screening (Supplementary Figure 2). The crystal structure model was then utilized for molecular docking screening against curcumin derivatives. Molecular docking was useful for the high-throughput virtual screening identification of several inhibitors [33]. From 317 curcumin derivatives, the best 100 compounds with the lowest docking score were collected for further screening (Supplementary Table 2). Among the top 20, curcumin derivatives possessed docking scores with a range from -10.2 to -11.5 kcal/mol, which was lower than that of Lapatinib, with a docking score of -8.1879 kcal/mol (Table 1). These findings verified the potency of curcumin derivatives as promising inhibitors of HER2.

### Construction of HER2 Mutations

Since the crystal structure of HER2 was still unavailable, this study performed an in silico mutation of HER2 using the MOE protocol [34]. We selected L755S, T798I, and T798M as the representative HER2 mutations that contribute to Lapatinib resistance [13–15]. A single point mutation in MOE was employed, followed by energy minimization and evaluation of the Phi-Psi geometry profile to identify the outlier. The location of all three mutations was in the kinase domain of HER2, which could affect the binding affinity of Lapatinib and other TKIs (Supplementary Figure 3A). The Phi-Psi angle in the Ramachandran plot indicated shifts in the secondary structure preferences for the L755S, T798I, and T798M mutations compared to typical regions in HER2 WT. These differences could reflect the structural destabilization or altered flexibility, which might contribute to changes in the drug resistance profile. Notably, there were only three to five outliers on the HER2 mutation model in this study, demonstrating a low disallowed region of less than 0.5% (Supplementary Figure 3B). These models were further used for molecular docking screening to identify the best inhibitors targeting HER2 mutations.

### Molecular Docking Screening of Curcumin Derivatives toward HER2 WT and Mutations

**Figure 2:**
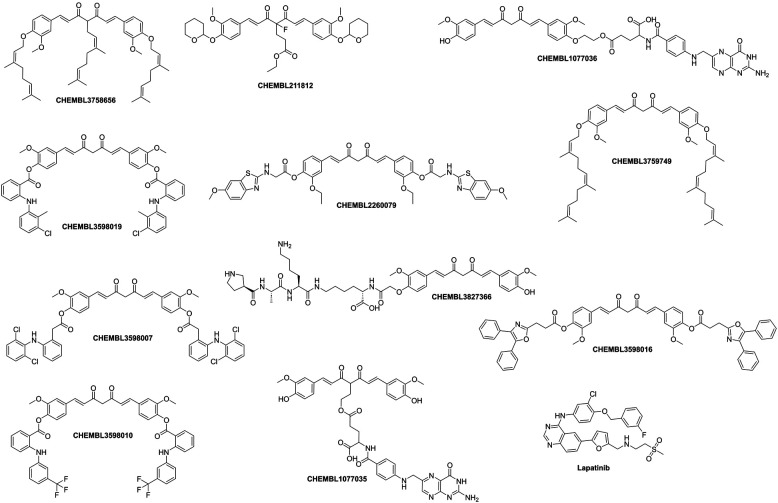
Chemical Structure of Lapatinib and Curcumin Derivatives with Lowest Docking Score against HER2 Mutants

The HER2 mutation model was then used for molecular docking screening of the top 100 curcumin derivatives. Previous virtual screening studies on HER2 mutations have demonstrated promising results in identifying potential inhibitors, highlighting the critical role of molecular docking screening in this process [34]. In our study, we found that several curcumin derivatives performed lower docking scores against Lapatinib for the HER2 L755S mutations, suggesting a higher binding affinity (Table 2). Similar trends were also observed for the HER2 T798I and HER2 T798M, where Lapatinib exhibited weaker binding affinities than curcumin derivatives (Table 2). Based on chemical structure analysis, the top five curcumin derivatives showed unique chemical structures. The lengths of each compound were comparable to that of ATP, which had a length of around 7-15 Å [35–37]. In addition, all of the curcumin derivatives also possessed diverse structural modifications that were essential for binding interaction [38–41]. Curcumin derivatives CHEMBL3758656 and CHEMBL3759749 have geranyl modifications on benzene substitution, which contribute to the lower docking score compared to other compounds (Figure 2). The tripeptide substitution curcumin derivative CHEMBL3827366 existed in the top five compounds in all HER2 mutations. Series of diphenylamine curcumin derivatives such as CHEMBL3598007, CHEMBL3598010, and CHEMBL3598019 also possessed considerable docking scores as the second or third position based on docking score (Figure 2). Introduction of pteroyl on curcumin derivatives as found on CHEMBL1077035 and CHEMBL1077036 showed as middle-rank HER2 inhibitor toward three mutations (Figure 2). Our study highlights the top curcumin derivatives exhibited unique chemical structure, diverse modifications, with specific substitutions contributing to their effectiveness as HER2 inhibitors. 

**Figure 3:**
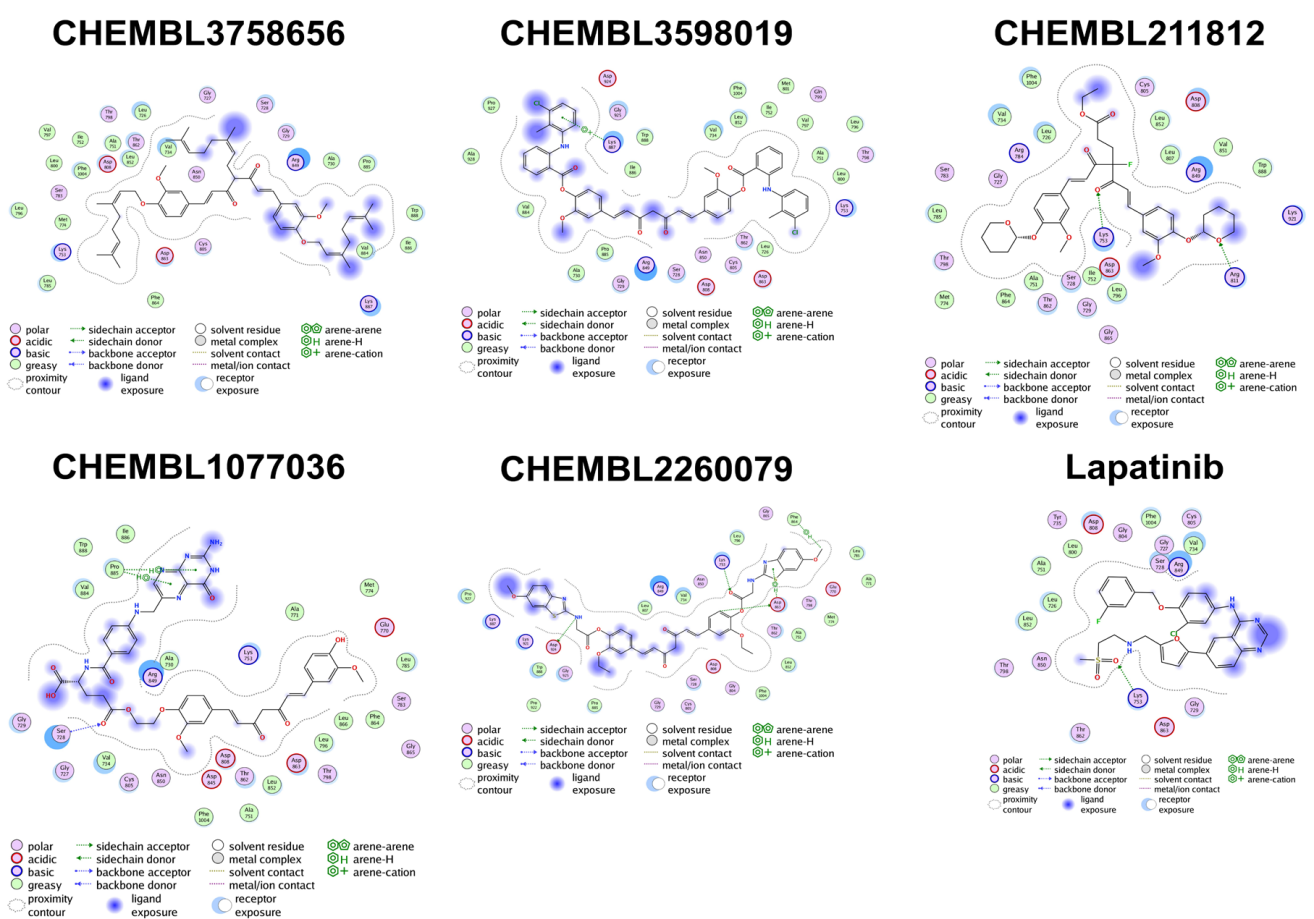
Binding Interaction of Lapatinib and Top Five Curcumin Derivatives against HER2 L755S in 2D Visualization

**Figure 4:**
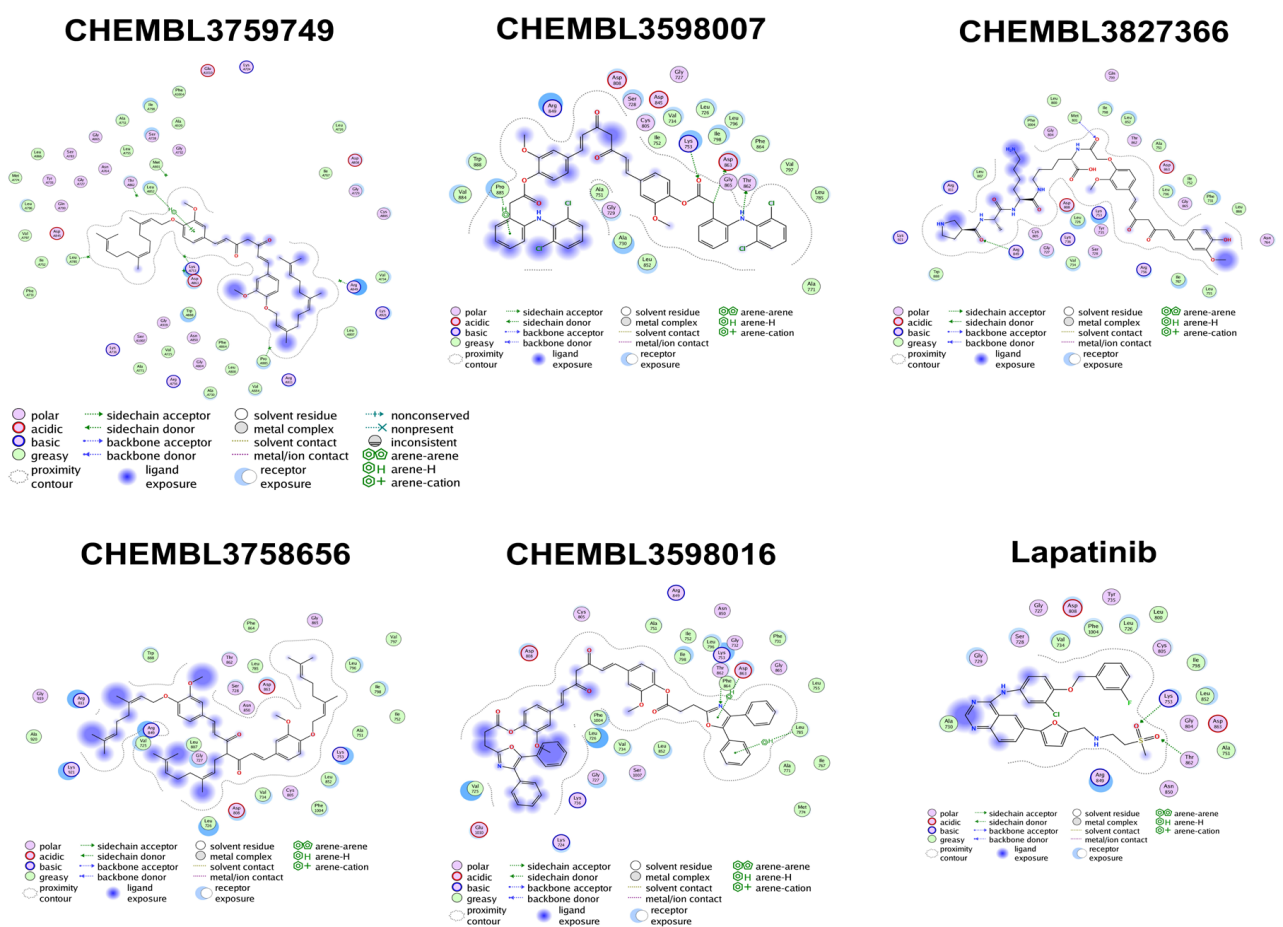
Binding Interaction of Lapatinib and Top Fve Curcumin Derivatives against HER2 T798I in 2D Visualization

**Figure 5:**
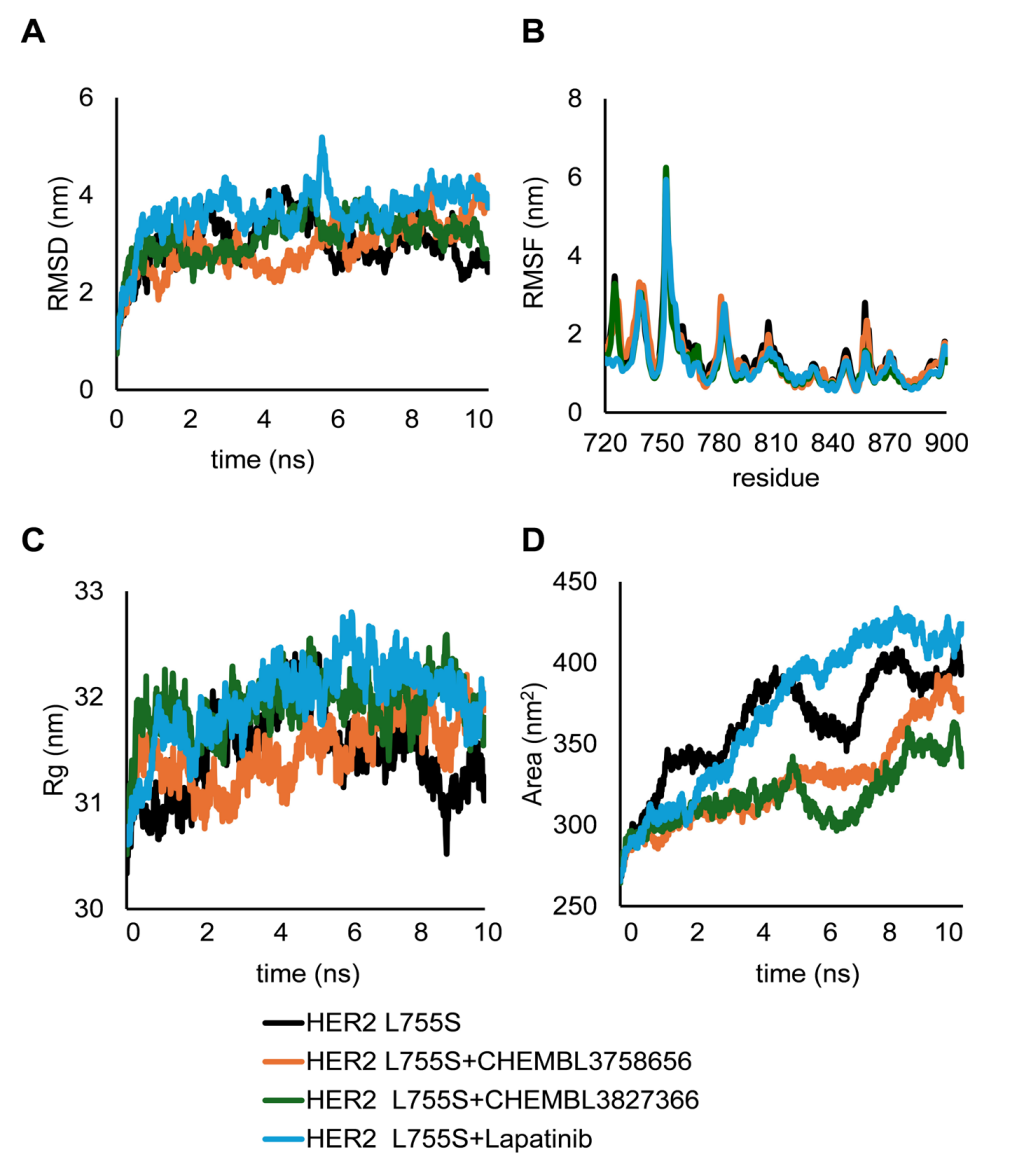
Binding Interaction of Lapatinib and Top Five Curcumin Derivatives against HER2T798M in 2D Visualization

Binding interaction analysis showed that curcumin derivatives shared a similar binding interaction profile with Lapatinib. Lapatinib in all HER2 mutations bound to two key amino acids such as Asp863 and Pro811 (Figure 3-5; Supplementary Figure 4-6). However, curcumin derivatives bound to several key amino acids in the kinase domain of HER L755S, such as Ser728, Lys753, Arg811, Asp863, Pro885, and Lys887 (Figure 3; Supplementary Figure 4). Disruption on Lys753 and Lys887 could affect the structural integrity of kinase domain during HER2 activation [42–44]. In HER2 T798I, Lapatinib and curcumin derivatives formed direct hydrogen bonds and arene bonds toward Lys753, Leu785, Arg849, Leu852, Thr862, Asp863, and Pro885 (Figure 4; Supplementary Figure 5). Binding toward Leu785 could affect the overall conformation of kinase domain, while interaction on Thr862 could disrupt the stability of loop in HER2 active conformation [45–47]. Curcumin derivatives and Lapatinib interacted with HER2 T798M by forming several bonds toward Phe731, Met798, Met801, Asp808, Arg811, Asp863, and Leu866 (Figure 5; Supplementary Figure 6). Interaction with Met798 and Met801 as the primary mutation location could alter the HER2 activation on the kinase domain [48–50]. Overall, our molecular docking screening revealed that several curcumin derivatives exhibited higher binding affinities than Lapatinib in HER2 mutations, with key interactions potentially disrupting kinase domain activation.

### Molecular dynamic simulation of Selected Curcumin Derivatives in complex with HER2 Mutations

**Figure 6:**
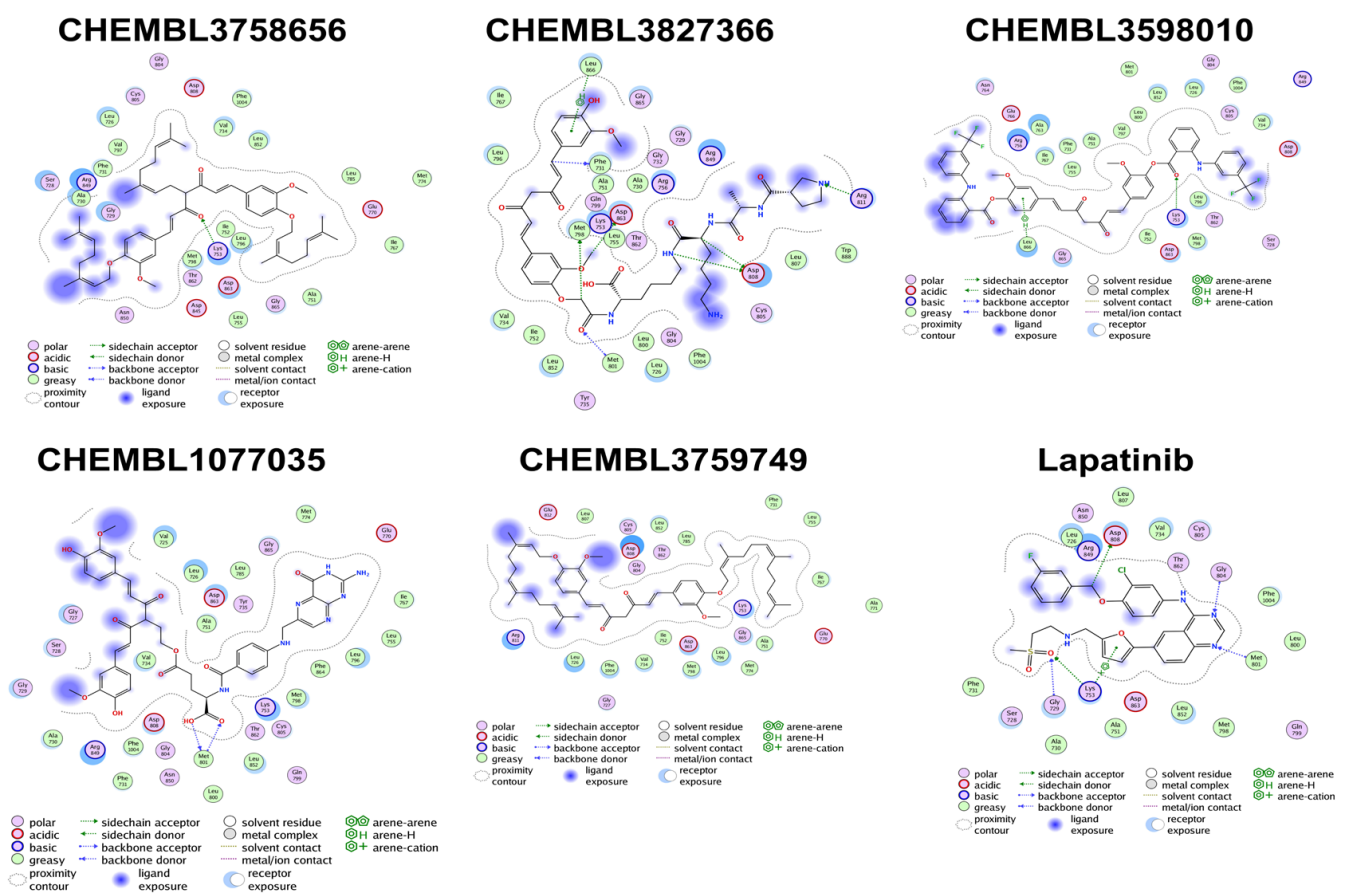
The RMSD (A), RMSF (B), Rg (C), and SASA (D) value of Lapatinib and Curcumin Derivatives in Complex with HER2 L755S

**Figure 7:**
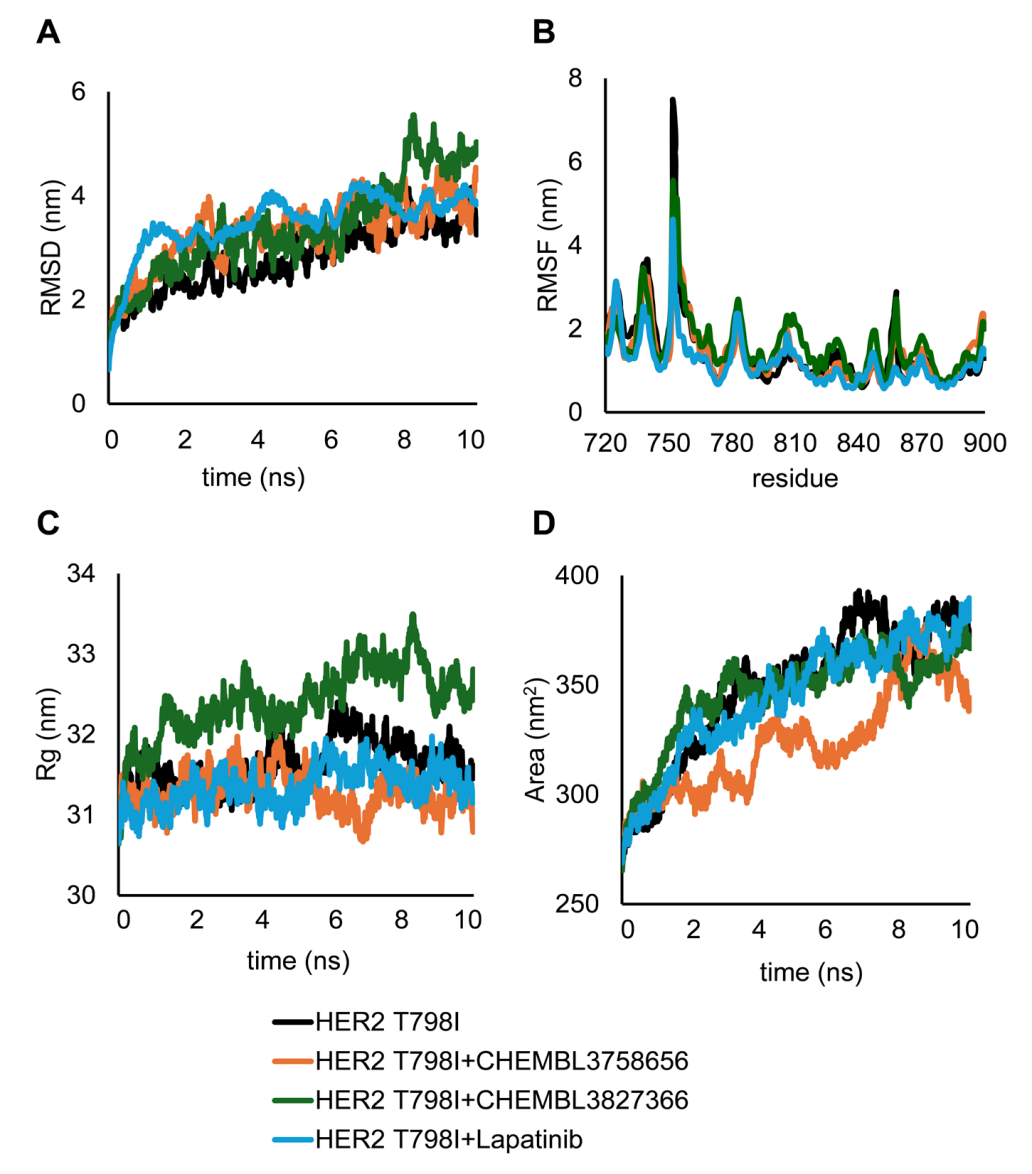
The RMSD (A), RMSF (B), Rg (C), and SASA (D) value of Lapatinib and Curcumin Derivatives in Complex with HER2 T798I

**Figure 8:**
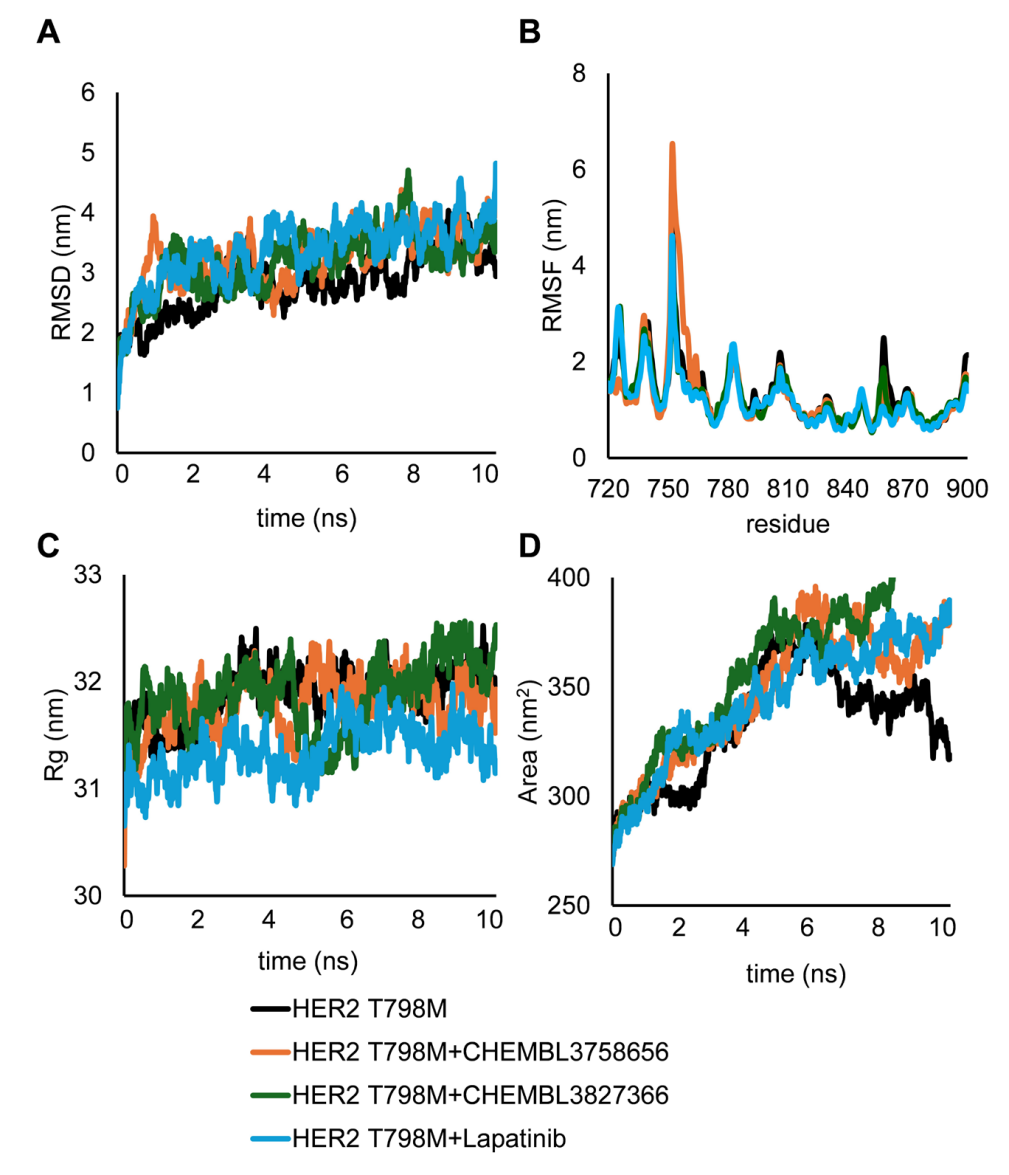
The RMSD (A), RMSF (B), Rg (C), and SASA (D) value of Lapatinib and Curcumin Derivatives in Complex with HER2 T798M

MD simulation complemented molecular docking by providing precise insight into the dynamic behavior and binding stability of protein-ligand complexes. From the molecular docking screening, CHEMBL3758656 and CHEMBL3827366 emerged as the top-performing curcumin derivatives and were selected for MD simulation to assess their binding properties further. After conducting 10 ns MD simulation, we analyzed several post-MD analyses, including RMSF, Rg, and SASA. For HER2 L755S, both CHEMBL3758656 and CHEMBL3827366 maintained lower RMSD values compared to Lapatinib, indicating enhanced binding stability (Figure 6A; Supplementary Table 3). RMSF analysis showed minimal differences across all complexes, suggesting that ligand binding had no effect on local flexibility (Figure 6B; Supplementary Table 3). The Rg profiles indicated that CHEMBL3827366 promoted slightly more compact protein conformation than other compounds (Figure 6C; Supplementary Table 3). In addition, SASA measurements revealed that both CHEMBL3758656 and CHEMBL3827366 binding reduced solvent exposure more effectively, implying a tighter overall packing of the ligand-protein complex (Figure 6D; Supplementary Table 3). In the case of HER2 T798I, the binding of CHEMBL3758656 and CHEMBL3827366 resulted in higher RMSD values relative to Lapatinib, reflecting increased structural flexibility and reduced binding stability (Figure 7A; Supplementary Table 3). The T798I gatekeeper was bulkier and more hydrophobic, which reduced ATP-site volume and altered local packing [15]. The engagement of curcumin derivatives could be spanning the activation of loop region resulting in elevation of RMSD and Rg. RMSF patterns remained largely consistent across all complexes, though Rg analysis indicated that CHEMBL3827366 induced a more expanded protein structure (Figure 7B and C; Supplementary Table 3). This observation was further supported by the SASA profile, which showed an increase in surface area in the presence of CHEMBL3827366, consistent with a less compact conformation that CHEMBL3758656 and Lapatinib (Figure 7D; Supplementary Table 3). For HER2 T798M, RMSD profiles showed comparable trends among all complexes, with CHEMBL3827366 inducing slightly greater fluctuations (Figure 8A; Supplementary Table 3). The RMSF analysis indicated stable local flexibility regardless of the bound ligand (Figure 8B; Supplementary Table 3). Rg data demonstrated that both CHEMBL3758656 and CHEMBL3827366 showed stable Rg profile, suggesting a compact conformation (Figure 8C; Supplementary Table 3). Correspondingly, SASA analysis revealed an increased solvent exposure in both CHEMBL3758656 and CHEMBL3827366 complex, whereas Lapatinib binding resulted in a less solvent-exposed structure (Figure 8D; Supplementary Table 3). Collectively, these results suggested that curcumin derivative CHEMBL3758656 and CHEMBL3827366 modulated HER2 mutations dynamic in mutation-dependent manner, stabilizing HER2 L755S while promoting structural destabilization in HER2 T798I and T798M, which could have implications for its inhibitory activity.

MD simulations of HER2 L755S, HER2 T798I, and HER2 T798M also revealed deeper insights into distinct conformational behaviors of these proteins in both unbound and ligand-bound states. In the absence of ligands, HER2 L755S demonstrated a relatively compact structural conformation, maintaining its structural integrity throughout the simulation (Supplementary Figure 7). In contrast, HER2 T798I and HER2 T798M exhibited a more flexible structure, with notable fluctuations in their overall structure (Supplementary Figure 8-9). Upon ligand binding, HER2 L755S maintained more stabilized conformation with both curcumin derivatives, showing minimal structural changes compared to its unbound form (Supplementary Figure 7). In HER2 T798I and HER2 T798M, which were already more flexible in their unbound states, underwent further conformational rearrangements when bound to curcumin derivatives, particularly around the ligand-binding region (Supplementary Figure 8 and 9). These changes were particularly pronounced in the binding pocket, where curcumin derivatives appeared to induce localized stabilization. However, Lapatinib induced more pronounced structural shifts in all three mutations, leading to greater conformational alterations and suggesting less effective stabilization than curcumin derivatives (Supplementary Figure 7-9). These findings highlighted that curcumin derivatives could stabilize the HER2 L755S, HER2 T798I and HER2 T798M through distinct binding mechanisms, offering potential as alternative therapeutic agents alongside established inhibitors such as Lapatinib.

Building on these conformational insights, the binding free energy ΔGbind values derived from MM-GBSA calculations further elucidated the differential binding affinities of curcumin derivatives and Lapatinib across HER2 mutations. The curcumin derivatives, CHEMBL3758656, consistently exhibited the strongest binding affinity across all HER2 mutations (Table 3). Moderate binding interaction was shown by CHEMBL3827366, while Lapatinib demonstrated the weakest binding across all HER2 mutations. These findings supported the superior stabilizing effect of CHEMBL3758656, reinforcing its potential as a lead compound for overcoming HER2 mutation-associated resistance through enhanced and selective binding interactions.

### ADME Properties of Curcumin Derivatives by pkCSM

Prediction of ADMET properties was crucial for evaluating the drug-like properties of compounds. The ADMET analysis could identify potential pharmacokinetic limitations and toxicity concerns early in drug development [51–53]. The ADMET predictions by pkCSM for CHEMBL3758656 and CHEMBL3827366 revealed several differences in their drug potential. CHEMBL3758656 exhibited a higher logP than CHEMBL3827366, indicating higher lipophilicity, which aligned with its lower water solubility (Table 4). However, CHEMBL3758656 demonstrated superior Caco-2 permeability and intestinal absorption, suggesting better bioavailability (Table 4). Both compounds shared similar skin permeability and total clearance value, while the tissue distribution of CHEMBL3758656 was lower than CHEMBL3827366 (Table 4). The maximum human-tolerated dose was higher for CHEMBL375865, implying a wider therapeutic window. In terms of toxicity, CHEMBL3827366 possessed higher oral rat acute and chronic toxicity, suggesting a better longer-term risk. Overall, both curcumin derivatives demonstrated comparable bioavailability, favorable toxicity profile, and acceptable pharmacokinetic balance, making them promising candidates for further drug development.

## Discussion

The primary goal of this study was to develop reported curcumin derivatives as HER2 inhibitors, particularly targeting Lapatinib resistance-associated HER2 mutations through multiple virtual screening. Lapatinib is used as the first line therapy for HER2-expressed cancer cells without or with other chemotherapy such as Trastuzumab or Capecitabine [54–57]. However, the increasing prevalence of HER2 mutations in breast cancer and their association with resistance to Lapatinib highlight the urgent need for further research on alternative therapeutic strategies [58–61]. HER2 mutations such as L755S, T798I, and T798M significantly alter the binding affinity of conventional inhibitors, reducing their efficacy and necessitating the development of novel compounds [62–64]. By leveraging computational methodologies, this study contributed to the ongoing search for targeting HER2 protein and its mutations related to Lapatinib resistance.

The integration of molecular docking and molecular dynamic simulation in our study effectively identified potential curcumin derivatives that targeted HER2 WT and its mutations. Notably, CHEMBL3758656 and CHEMBL3827366 consistently ranked among the top five compounds with low docking score across all three HER2 mutations. CHEMBL3758656, which incorporated a geranyl group on the hydroxyl groups of both phenolic rings and one of its C-alpha center, had been reported as a weak inhibitor of HDAC and mPGES-1 [65]. Moreover, CHEMBL3827366, a conjugated curcumin derivative linked to the fibrinogen-derived peptide fragment Pro-Ala-Lys, had demonstrated the ability to restore the mitochondrial reticular networks and promote cell survival [66]. Both compounds exhibited stable interaction with key amino acids in the kinase domain of HER2 despite their relatively large molecular sizes. MD analysis revealed that RMSD, Rg, and SASA patterns of curcumin derivatives were consistent with mutation-dependent modulation. The MM-GBSA scores of curcumin derivatives were also more superior than Lapatinib, implying enhanced structural stabilization of the kinase domain. Such stabilization may hinder conformational transitions necessary for ATP binding and phosphorylation, mimicking the biological inhibition mechanism [67]. Binding interaction also suggested that curcumin derivatives extended beyond the canonical ATP-binding cleft toward peripheral regions, potentially engaging allosteric sites. The ability to access such alternative binding modes is especially relevant for T798I and T798M, where steric hindrance could block traditional hinge-binding inhibitors [15]. Owing to the conserved structure of kinase domains, many small-molecule targeted therapies display varying degrees of selectivity and could interact with multiple kinases [67–69]. Several studies support the importance of large-sized small molecules in effectively targeting the HER2 kinase domain. For example, bulky substituents added to small molecule inhibitors, such as benzyl ether groups in quinazoline derivatives, have been shown to increase potency and selectivity toward HER2 by better occupying its larger hydrophobic pocket [70]. However, designing such molecules required careful balancing of size to maintain sufficient membrane permeability and favorable pharmacokinetic properties [71-73]. Our findings highlight the potential of curcumin derivatives, particularly CHEMBL3758656 and CHEMBL3827366, to interact with HER2 and its mutations.

Docking scores and binding free energies correlated well with predicted ADMET properties. ADMET predictions further supported the potential of CHEMBL3758656 and CHEMBL3827366 as drug candidates. CHEMBL3758656, with the lowest MM-GBSA energies, also showed favorable oral absorption and moderate lipophilicity which is associated with effective kinase inhibition. CHEMBL3827366, despite slightly higher docking energies, displayed an acceptable toxicity profile and solubility, suggesting potential for safer chronic administration. Both compounds exhibited favorable pharmacokinetic properties, including high intestinal absorption and acceptable toxicity profiles. The superior bioavailability of these curcumin derivatives enhances their therapeutic prospects. However, differences in lipophilicity and clearance rates suggest the need for further optimization to improve drug likeness and minimize potential side effects [74-76]. The limitation of this study was the exclusive use of curcumin derivatives sourced from the ChEMBL database, as well as its focus on only three mutations. In addition, future studies should focus on in vitro and in vivo validation of these compounds to confirm their efficacy and safety in biological systems.

In conclusion, this study demonstrates the potency of curcumin derivatives as an effective inhibitor of HER2, particularly in the context of mutations associated with Lapatinib resistance, such as L755S, T798I, and T798M. The findings underscore the results from virtual screening using 505 curcumin databases to identify the top two curcumin derivatives, CHEMBL3758656 and CHEMBL3827366, as the most promising compounds against three HER2 mutations and exhibited favorable ADMET profiles.

**Table 1:** Top 20 Curcumin Derivatives based on Molecular Docking Screening toward HER2 WT

No	CHEMBL ID	SMILES
1	Lapatinib	CS(=O)(=O)CCNCC1=CC=C(O1)C2=CC3=C(C=C2)N=CN=C3NC4=CC(=C(C=C4)OCC5=CC(=CC=C5)F)Cl
2	CHEMBL3758656	O=C(/C=C/c1cc(OC)c(OC/C=C(\CC/C=C(\C)/C)/C)cc1)C(C(=O)/C=C/c1cc(OC)c(OC/C=C(\CC/C=C(\C)/C)/C)cc1)C/C=C(\CC/C=C(\C)/C)/C
3	CHEMBL2259872	O=C(Oc1c(OCC)cc(/C=C/C(=O)CC(=O)/C=C/c2cc(OCC)c(OC(=O)CNCCN(CC)CC)cc2)cc1)CNCCN(CC)CC
4	CHEMBL1077035	O=C(O)C(NC(=O)c1ccc(NCc2nc3C(=O)NC(N)=Nc3nc2)cc1)CCC(=O)OCCC(C(=O)/C=C/c1cc(OC)c(O)cc1)C(=O)/C=C/c1cc(OC)c(O)cc1
5	CHEMBL3759699	O=C(C(C(=O)/C=C/c1cc(OC)c(OC/C=C(\CC/C=C(\C)/C)/C)cc1)(C/C=C(\CC/C=C(\C)/C)/C)C/C=C(\CC/C=C(\C)/C)/C)/C=C/c1cc(OC)c(OC/C=C(\CC/C=C(\C)/C)/C)cc1
6	CHEMBL3598007	Clc1c(Nc2c(CC(=O)Oc3c(OC)cc(/C=C/C(=O)CC(=O)/C=C/c4cc(OC)c(OC(=O)Cc5c(Nc6c(Cl)cccc6Cl)cccc5)cc4)cc3)cccc2)c(Cl)ccc1
7	CHEMBL3598018	O=C(/C=C/c1cc(OC)c(OC(=O)c2c(OC(=O)c3c(O)cccc3)cccc2)cc1)CC(=O)/C=C/c1cc(OC)c(OC(=O)c2c(OC(=O)c3c(O)cccc3)cccc2)cc1
8	CHEMBL3598010	FC(F)(F)c1cc(Nc2c(C(=O)Oc3c(OC)cc(/C=C/C(=O)CC(=O)/C=C/c4cc(OC)c(OC(=O)c5c(Nc6cc(C(F)(F)F)ccc6)cccc5)cc4)cc3)cccc2)ccc1
9	CHEMBL3827366	O=C(O)[C@@H](NC(=O)COc1c(OC)cc(/C=C/C(=O)CC(=O)/C=C/c2cc(OC)c(O)cc2)cc1)CCCCNC(=O)[C@@H](NC(=O)[C@@H](NC(=O)[C@@H]1CNCC1)C)CCCCN
10	CHEMBL3827366	O=C(Oc1c(OC)cc(/C=C/C(=O)CC(=O)/C=C/c2cc(OC)c(OC(=O)CCc3oc(c(-c4ccccc4)n3)-c3ccccc3)cc2)cc1)CCc1oc(c(-c2ccccc2)n1)-c1ccccc1
11	CHEMBL2260092	O=C(Oc1c(OC)cc(/C=C/C(=O)CC(=O)/C=C/c2cc(OC)c(OC(=O)CNCCN(C)C)cc2)cc1)CNCCN(C)C
12	CHEMBL2259876	S(=O)(=O)(N)c1ccc(NCC(=O)Oc2c(OC)cc(/C=C/C(=O)CC(=O)/C=C/c3cc(OC)c(OC(=O)CNc4ccc(S(=O)(=O)N)cc4)cc3)cc2)cc1
13	CHEMBL444980	O=C(Oc1c(OC)cc(/C=C/C(=O)CC(=O)/C=C/c2cc(OC)c(OC(=O)[C@@H](N)CCC(=O)O)cc2)cc1)[C@@H](N)CCC(=O)O
14	CHEMBL3827416	O=C(O)[C@@H](NC(=O)COc1c(OC)cc(/C=C/C(=O)CC(=O)/C=C/c2cc(OC)c(OC)cc2)cc1)CCCCNC(=O)[C@@H](NC(=O)[C@@H](NC(=O)[C@@H]1CNCC1)C)CCCCN
15	CHEMBL3827416	O=C(/C(/C(=O)/C=C/c1cc(OC)c(OC2OCCCC2)cc1)=C\C=C(/OCC)\O)/C=C/c1cc(OC)c(OC2OCCCC2)cc1
16	CHEMBL2260076	O=C(Oc1c(OC)cc(/C=C/C(=O)CC(=O)/C=C/c2cc(OC)c(OC(=O)CCNc3sc(C)nn3)cc2)cc1)CCNc1sc(C)nn1
17	CHEMBL2260076	O=C(/C=C/c1cc(OC)c(OCCN2CCN(Cc3ccc(-c4oc(-c5cc(Nc6nc(-c7cnccc7)ccn6)c(C)cc5)nn4)cc3)CC2)cc1)CC(=O)/C=C/c1cc(OC)c(O)cc1
18	CHEMBL2260089	O=C(Oc1c(OC)cc(/C=C/C(=O)CC(=O)/C=C/c2cc(OC)c(OC(=O)CNc3sc(C)nn3)cc2)cc1)CNc1sc(C)nn1
19	CHEMBL2259874	O=C(Oc1c(OCC)cc(/C=C/C(=O)CC(=O)/C=C/c2cc(OCC)c(OC(=O)CCNCCN(C)C)cc2)cc1)CCNCCN(C)C
20	CHEMBL3558372	O=C(/C=C/c1cc(OC)c(OCCCCC[n+]2ccccc2)cc1)CC(=O)/C=C/c1cc(OC)c(OCCCCC[n+]2ccccc2)cc1
21	CHEMBL3598014	O=C(/C=C/c1cc(OC)c(OC(=O)c2c(Nc3c(C)c(C)ccc3)cccc2)cc1)CC(=O)/C=C/c1cc(OC)c(OC(=O)c2c(Nc3c(C)c(C)ccc3)cccc2)cc1

**Table 2:** The Docking Score of Lapatinib and the Top Five Curcumin Derivatives based on the Lowest Docking Score against HER2 Mutation

HER2 L775S			
No	CHEMBL ID	SMILES	Docking Score (kcal/mol)
1	Lapatinib	CS(=O)(=O)CCNCC1=CC=C(O1)C2=CC3=C(C=C2)N=CN=C3NC4=CC(=C(C=C4)OCC5=CC(=CC=C5)F)Cl	-7.4397
2	CHEMBL3758656	O=C(/C=C/c1cc(OC)c(OC/C=C(\CC/C=C(\C)/C)/C)cc1)C(C(=O)/C=C/c1cc(OC)c(OC/C=C(\CC/C=C(\C)/C)/C)cc1)C/C=C(\CC/C=C(\C)/C)/C	-12.6378
3	CHEMBL3598019	Clc1c(C)c(Nc2c(C(=O)Oc3c(OC)cc(/C=C/C(=O)CC(=O)/C=C/c4cc(OC)c(OC(=O)c5c(Nc6c(C)c(Cl)ccc6)cccc5)cc4)cc3)cccc2)ccc1	-11.9414
4	CHEMBL211812	FC(C(=O)/C=C/c1cc(OC)c(OC2OCCCC2)cc1)(C(=O)/C=C/c1cc(OC)c(OC2OCCCC2)cc1)CCC(=O)OCC	-11.7109
5	CHEMBL1077036	O=C(O)C(NC(=O)c1ccc(NCc2nc3C(=O)NC(N)=Nc3nc2)cc1)CCC(=O)OCCOc1c(OC)cc(/C=C/C(=O)CC(=O)/C=C/c2cc(OC)c(O)cc2)cc1	-11.6619
6	CHEMBL2260079	O=C(Oc1c(OCC)cc(/C=C/C(=O)CC(=O)/C=C/c2cc(OCC)c(OC(=O)CNc3sc4c(n3)ccc(OC)c4)cc2)cc1)CNc1sc2c(n1)ccc(OC)c2	-11.6369
HER2 T798I			
No	CHEMBL ID	SMILES	Docking Score (kcal/mol)
1	Lapatinib	CS(=O)(=O)CCNCC1=CC=C(O1)C2=CC3=C(C=C2)N=CN=C3NC4=CC(=C(C=C4)OCC5=CC(=CC=C5)F)Cl	-8.2521
2	CHEMBL3759749	O=C(/C=C/c1cc(OC)c(OC/C=C(\CC/C=C(\CC/C=C(\C)/C)/C)/C)cc1)CC(=O)/C=C/c1cc(OC)c(OC/C=C(\CC/C=C(\CC/C=C(\C)/C)/C)/C)cc1	-12.6237
3	CHEMBL3598007	Clc1c(Nc2c(CC(=O)Oc3c(OC)cc(/C=C/C(=O)CC(=O)/C=C/c4cc(OC)c(OC(=O)Cc5c(Nc6c(Cl)cccc6Cl)cccc5)cc4)cc3)cccc2)c(Cl)ccc1	-11.7243
4	CHEMBL3827366	O=C(O)[C@@H](NC(=O)COc1c(OC)cc(/C=C/C(=O)CC(=O)/C=C/c2cc(OC)c(O)cc2)cc1)CCCCNC(=O)[C@@H](NC(=O)[C@@H](NC(=O)[C@@H]1CNCC1)C)CCCCN	-11.4027
5	CHEMBL3758656	O=C(/C=C/c1cc(OC)c(OC/C=C(\CC/C=C(\C)/C)/C)cc1)C(C(=O)/C=C/c1cc(OC)c(OC/C=C(\CC/C=C(\C)/C)/C)cc1)C/C=C(\CC/C=C(\C)/C)/C	-11.1608
6	CHEMBL3598016	O=C(Oc1c(OC)cc(/C=C/C(=O)CC(=O)/C=C/c2cc(OC)c(OC(=O)CCc3oc(c(-c4ccccc4)n3)-c3ccccc3)cc2)cc1)CCc1oc(c(-c2ccccc2)n1)-c1ccccc1	-10.8619
HER2 T798M			
No	CHEMBL ID	SMILES	Docking Score (kcal/mol)
1	Lapatinib	CS(=O)(=O)CCNCC1=CC=C(O1)C2=CC3=C(C=C2)N=CN=C3NC4=CC(=C(C=C4)OCC5=CC(=CC=C5)F)Cl	-7.9005
2	CHEMBL3758656	O=C(/C=C/c1cc(OC)c(OC/C=C(\CC/C=C(\C)/C)/C)cc1)C(C(=O)/C=C/c1cc(OC)c(OC/C=C(\CC/C=C(\C)/C)/C)cc1)C/C=C(\CC/C=C(\C)/C)/C	-12.3487
3	CHEMBL3827366	O=C(O)[C@@H](NC(=O)COc1c(OC)cc(/C=C/C(=O)CC(=O)/C=C/c2cc(OC)c(O)cc2)cc1)CCCCNC(=O)[C@@H](NC(=O)[C@@H](NC(=O)[C@@H]1CNCC1)C)CCCCN	-12.1223
4	CHEMBL3598010	FC(F)(F)c1cc(Nc2c(C(=O)Oc3c(OC)cc(/C=C/C(=O)CC(=O)/C=C/c4cc(OC)c(OC(=O)c5c(Nc6cc(C(F)(F)F)ccc6)cccc5)cc4)cc3)cccc2)ccc1	-11.7694
5	CHEMBL1077035	O=C(O)C(NC(=O)c1ccc(NCc2nc3C(=O)NC(N)=Nc3nc2)cc1)CCC(=O)OCCC(C(=O)/C=C/c1cc(OC)c(O)cc1)C(=O)/C=C/c1cc(OC)c(O)cc1	-11.6999
6	CHEMBL3759749	O=C(/C=C/c1cc(OC)c(OC/C=C(\CC/C=C(\CC/C=C(\C)/C)/C)/C)cc1)CC(=O)/C=C/c1cc(OC)c(OC/C=C(\CC/C=C(\CC/C=C(\C)/C)/C)/C)cc1	-11.5126

**Table 3:** MM-GBSA Binding Free Energy Profiles of Curcumin Derivatives and Lapatinib with HER2 Mutations

HER2 L755S			
Compound	E_ele_ (Kcal/mol)	E_vdw_ (Kcal/mol)	ΔG_bind_ (Kcal/mol)*
HER2 L755S+CHEMBL3758656	-6.4195	-67.3083	-40.3454±0.2076
HER2 L755S+CHEMBL3827366	-74.8577	-69.5355	-47.6623±0.143
HER2 L755S+Lapatinib	-6.3196	-52.5136	-33.7071±0.1162
HER2 T798I			
Compound	E_ele_ (Kcal/Mol)	E_vdw_ (Kcal/Mol)	ΔG_bind_ (Kcal/Mol)*
HER2 T798I+CHEMBL3758656	-5.8824	-80.5161	-59.3356±0.1608
HER2 T798I+CHEMBL3827366	-74.068	-66.3645	-48.1509±0.1479
HER2 T798I+Lapatinib	-4.2536	-53.2063	-32.5919±0.0983
HER2 T798M			
Compound	E_ele_ (Kcal/Mol)	Evdw (Kcal/Mol)	ΔGbind (Kcal/Mol)*
HER2T798M+CHEMBL3758656	-5.9529	-79.5778	-59.688±0.1374
HER2 T798M+CHEMBL3827366	-74.5382	-69.546	-52.0749±0.1654
HER2 T798M+Lapatinib	-5.193	-54.6709	-32.4044±0.1553

*Data was expressed in mean±SD

**Table 4:** ADMET Prediction of Curcumin Derivatives

Parameter	CHEMBL3758656	CHEMBL3827366
logP	13.4106	1.627
Water Solubility (log mol/L)	-3.145	-2.899
Caco-2 Permeability (log Papp in 10-6 cm/s)	1.217	0.626
Intestinal Absorption (% Absorbed)	93.228	4.232
Skin Permeability (log Kp)	-2.735	-2.735
VDss (log L/kg)	-1.208	0.843
Total Clearance (log ml/min/kg)	0.999	0.864
Max. Human Tolerated Dose (Log mg/kg/day)	0.619	0.432
Oral Rat Acute Toxicity (mol/kg)	1.831	2.607
Oral Rat Chronic Toxicity (log mg/kg_bw/day)	0.467	3.93

## Author Contribution Statement

Mantiqa Syafa Duvadillan Gusrin: Data Curation, Analysis, Writing, and Finalization of Original Draft. Yonika Arum Larasati: Analysis, Writing, and Finalization of Original Draft. Rohmad Yudi Utomo: Conceptualization, Data curation, Funding Acquisition, Methodology, Supervision, Writing, and Finalization of Original Draft.
