# Supplementary material for: Comprehensive Molecular Docking and Molecular Dynamics Reveal Inhibitors of HER2 L755S, T798I, and T798M based on a Large Database of Curcumin Derivatives
Source: Asian Pac J Cancer Prev. 2026 Jan 22;27(1):265–79. doi: 10.31557/APJCP.2026.27.1.265 (PMC13418042; doi:10.31557/APJCP.2026.27.1.265)
Supplement: Figure S1-S9 [file APJCP-27-1-265-s001.pdf]

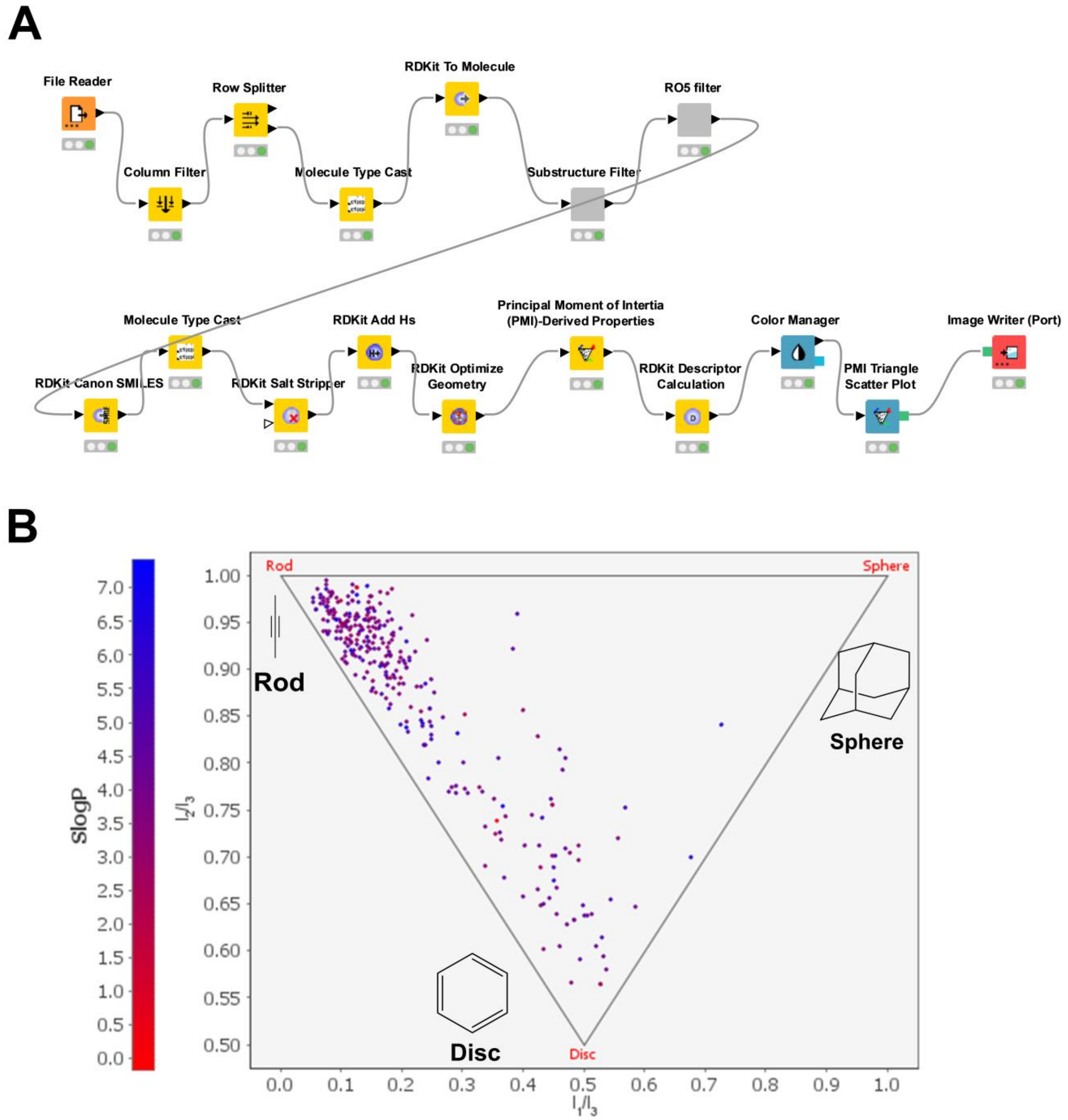

Supplementary Fig. (1) Curation and analysis of curcumin derivatives database. (A) KNIME workflow for filtering curcumin derivatives. (B) PMI analysis of retrieved curcumin derivatives from the database

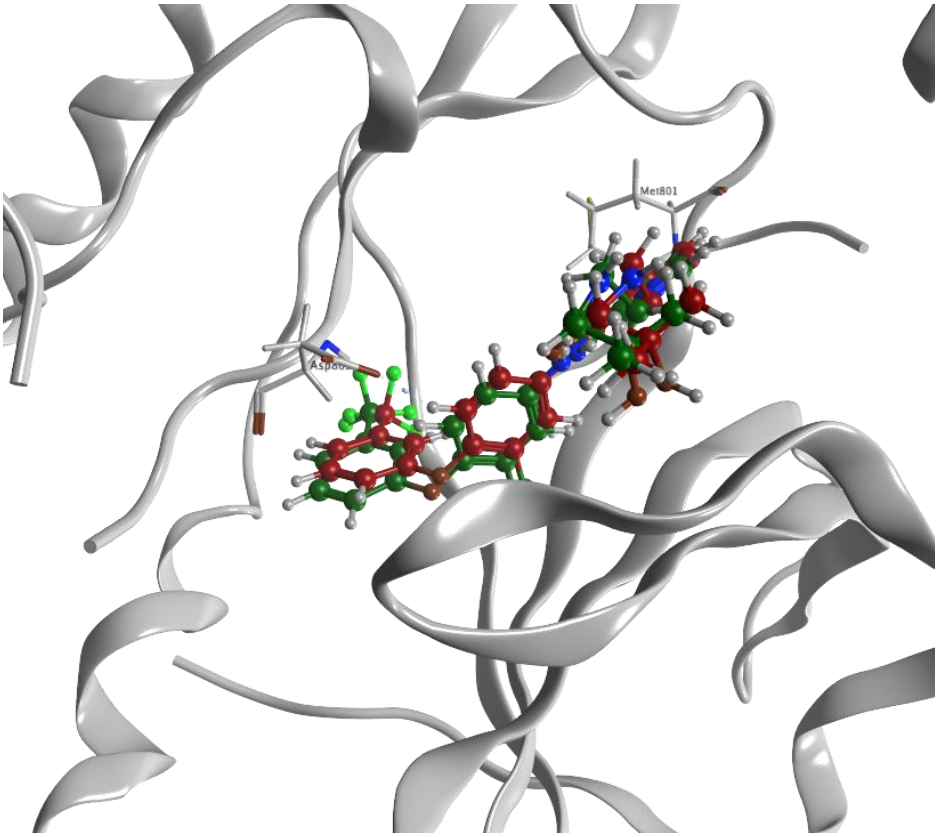

Supplementary Fig. (2) The crystal structure of the redocking study used TAK-285 as the native ligand. Protein was illustrated as a grey ribbon while compounds and interacting amino acids were demonstrated as ball stick color

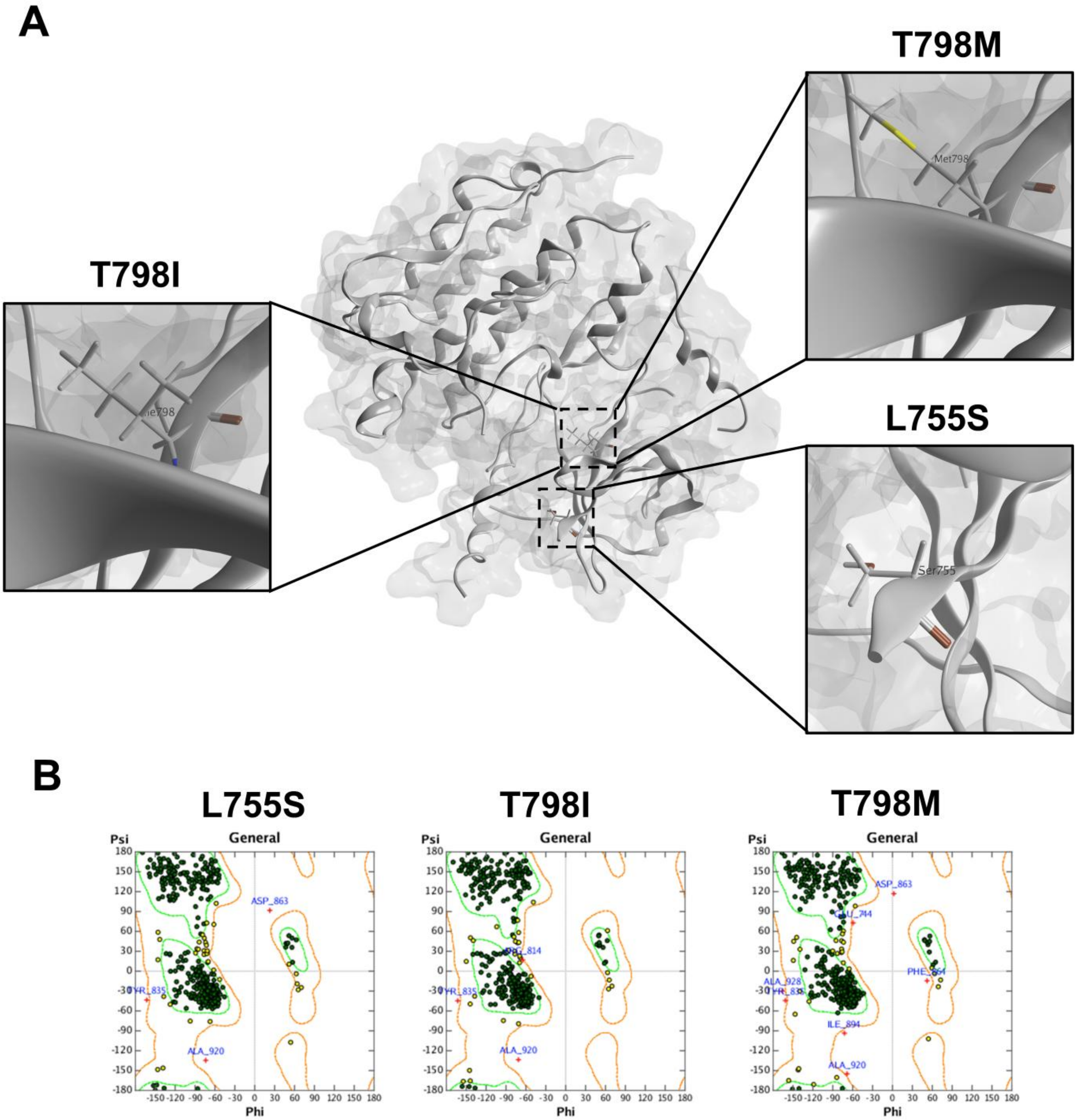

Supplementary Fig. (3) Structural and conformational analysis of HER2 mutations (L755S, T798I, and T798M) and their Ramachandran plot. (A) Overal structure of kinase domain highlighting the mutation site. (B) Ramachandran plots for mutated residues. The green dots represent the conformational spaces of the Phi-Psi angles for mutated residues. Deviations from typically allowed regions (orange outlines) suggest changes in backbone conformational preferences

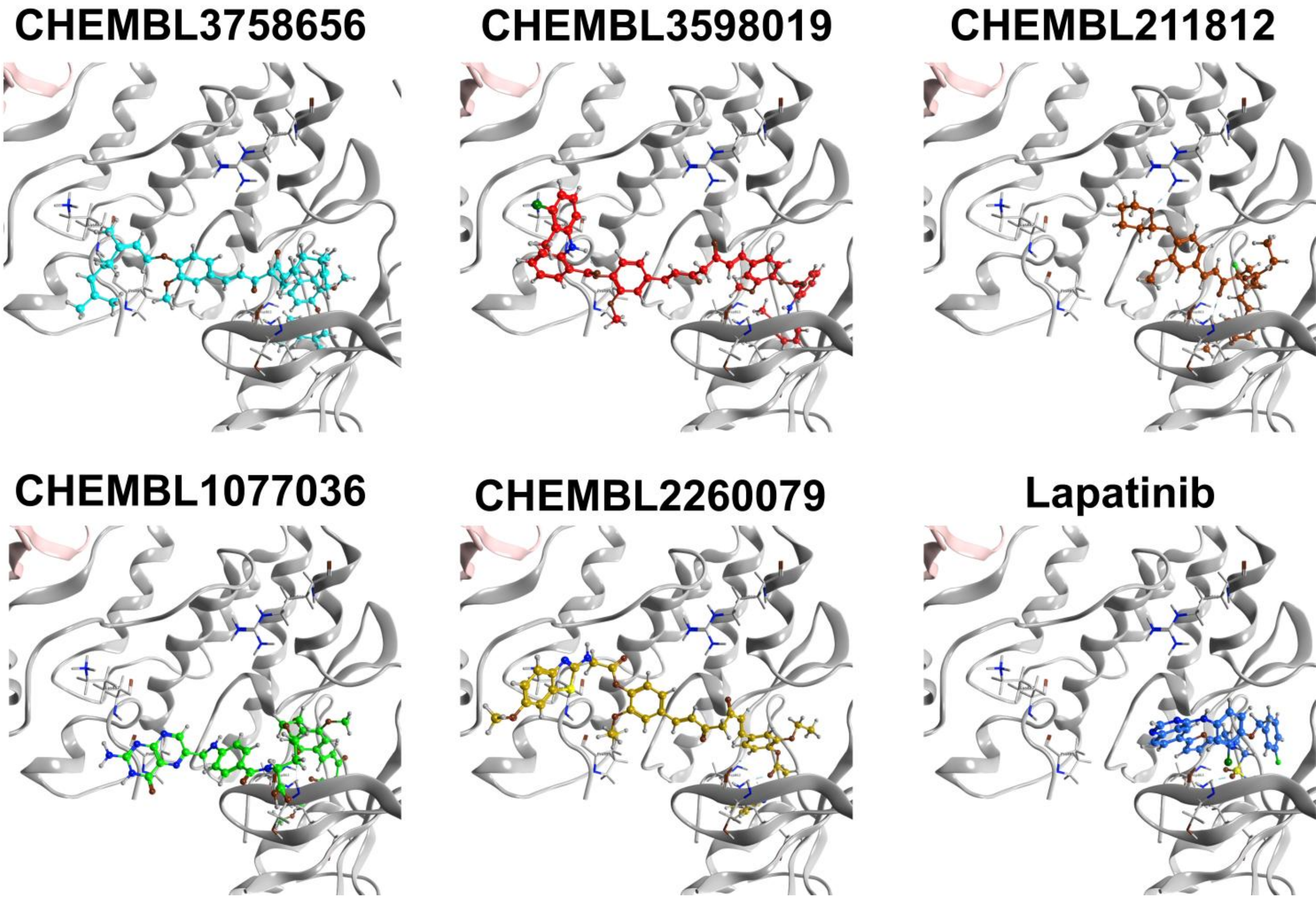

Supplementary Fig. (4) Binding interaction of Lapatinib and top five Curcumin Derivatives against HER2 L755S in 3D Visualization

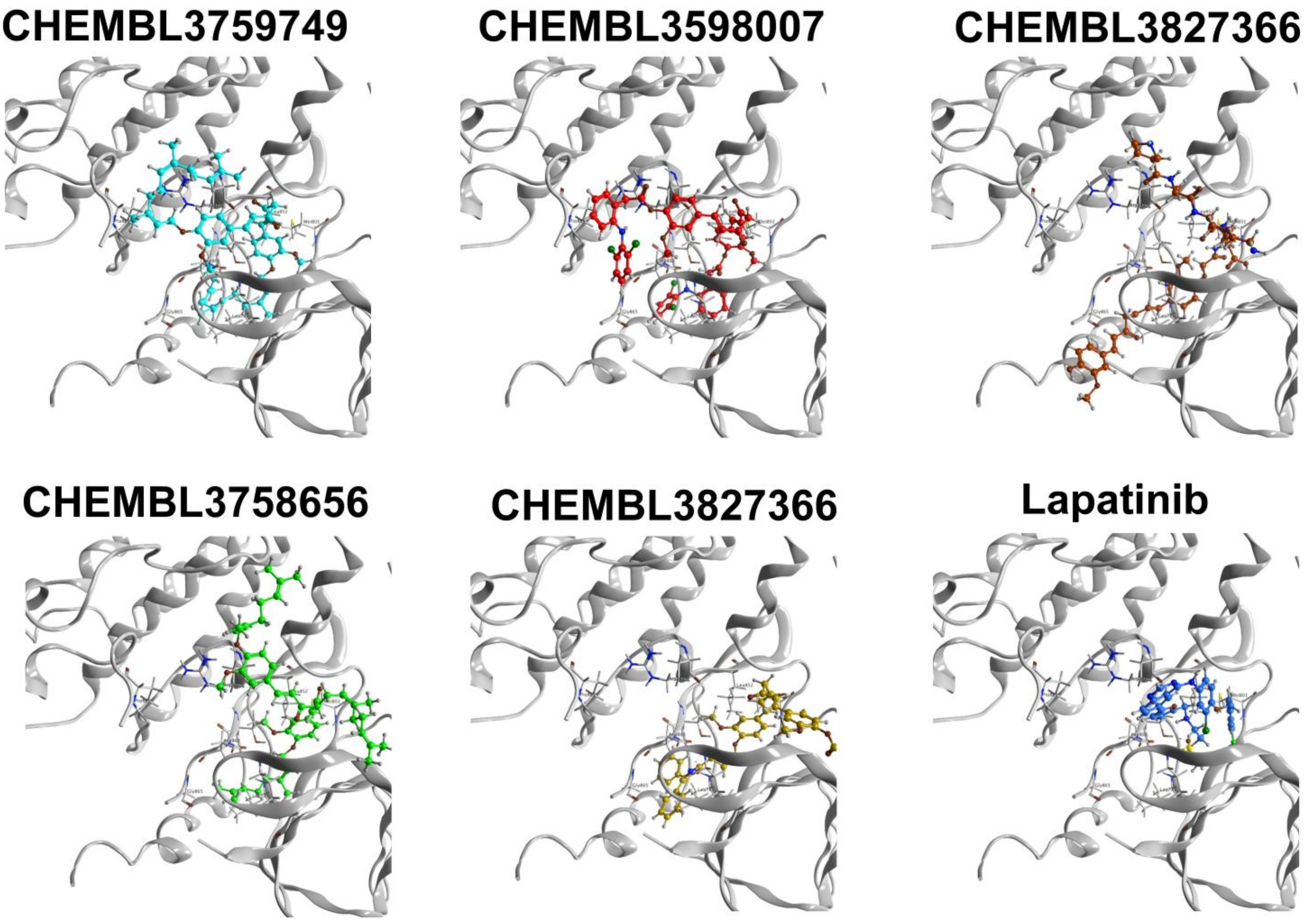

Supplementary Fig. (5) Binding interaction of Lapatinib and top five Curcumin Derivatives against HER2 T798I in 3D Visualization

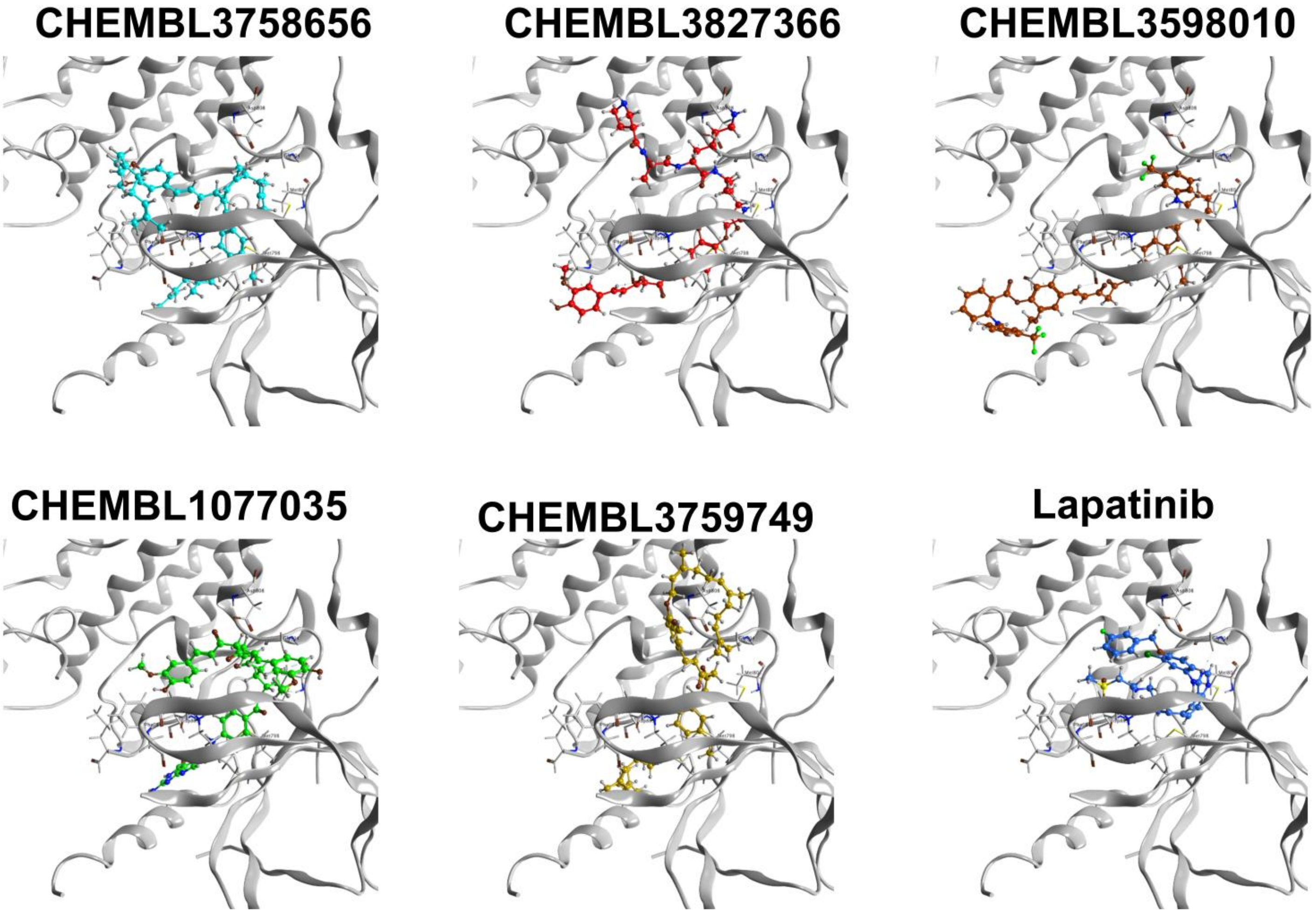

Supplementary Fig. (6) Binding interaction of Lapatinib and top five Curcumin Derivatives against HER2T798M in 3D Visualization

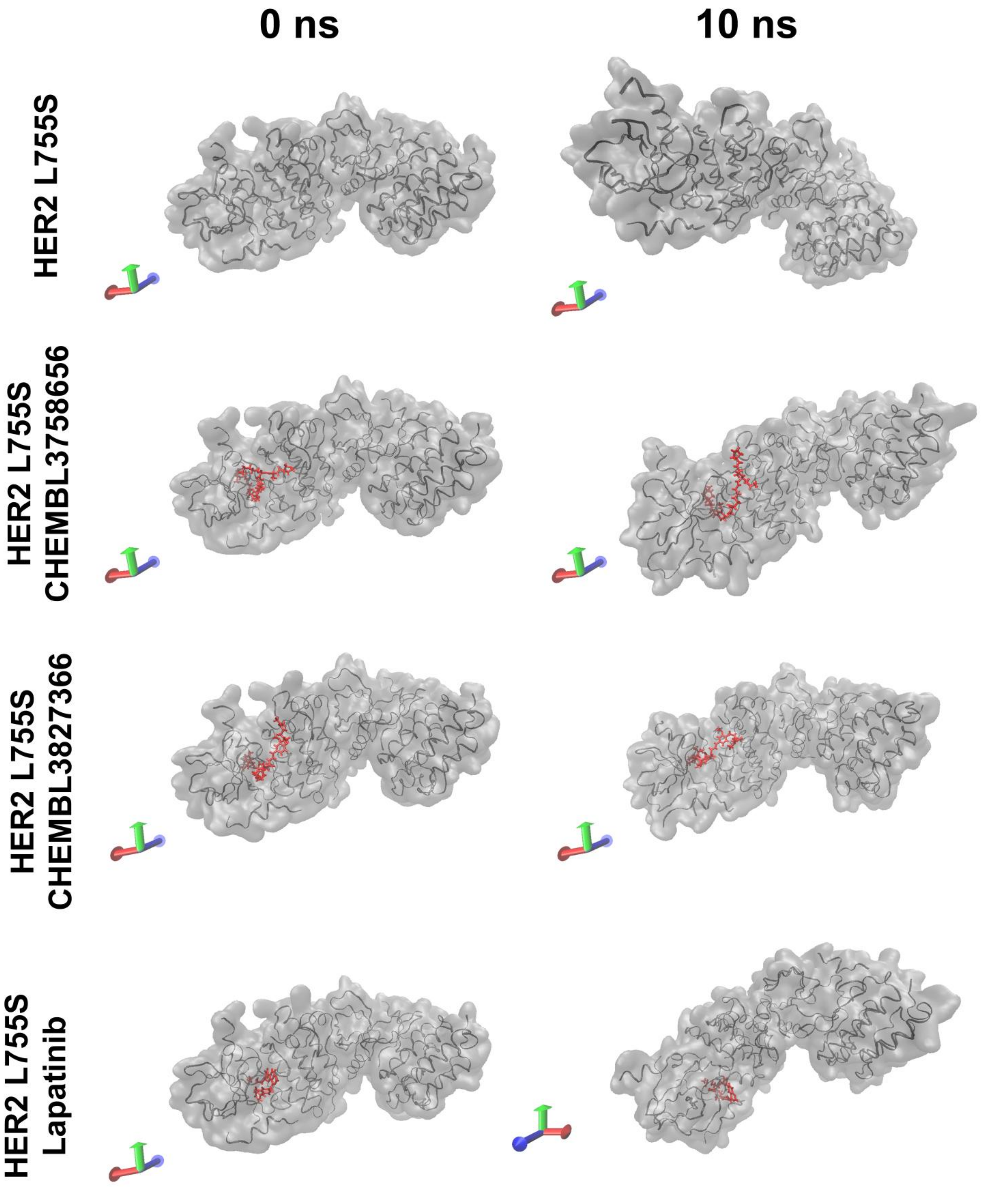

Supplementary Fig. (7). Visualization of the binding interaction of curcumin derivatives againts HER2 L755S

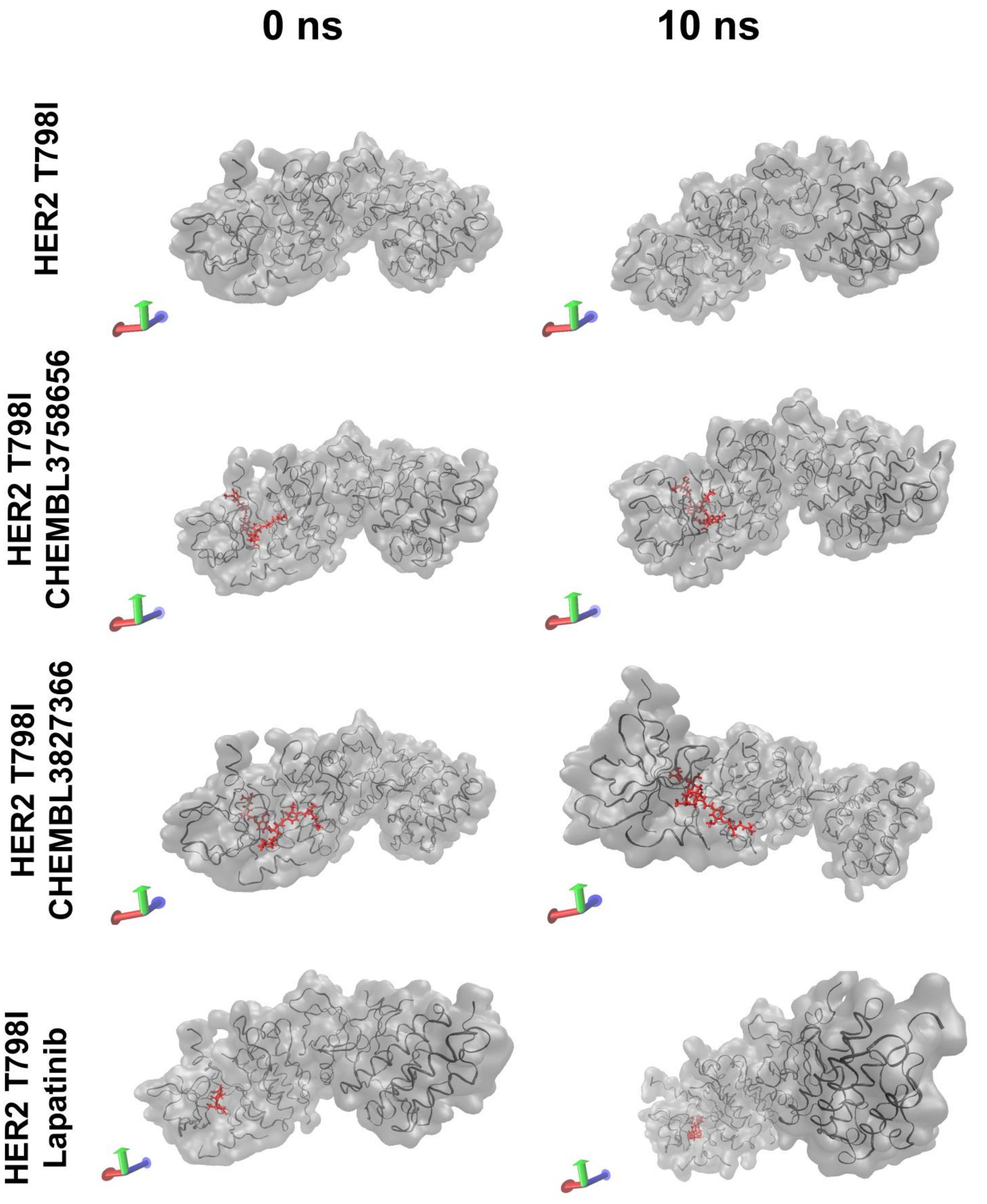

Supplementary Fig. (8). Visualization of the binding interaction of curcumin derivatives againts HER2 T798I Images are useful but would benefit from structural annotations (e.g., ligand conformation changes).

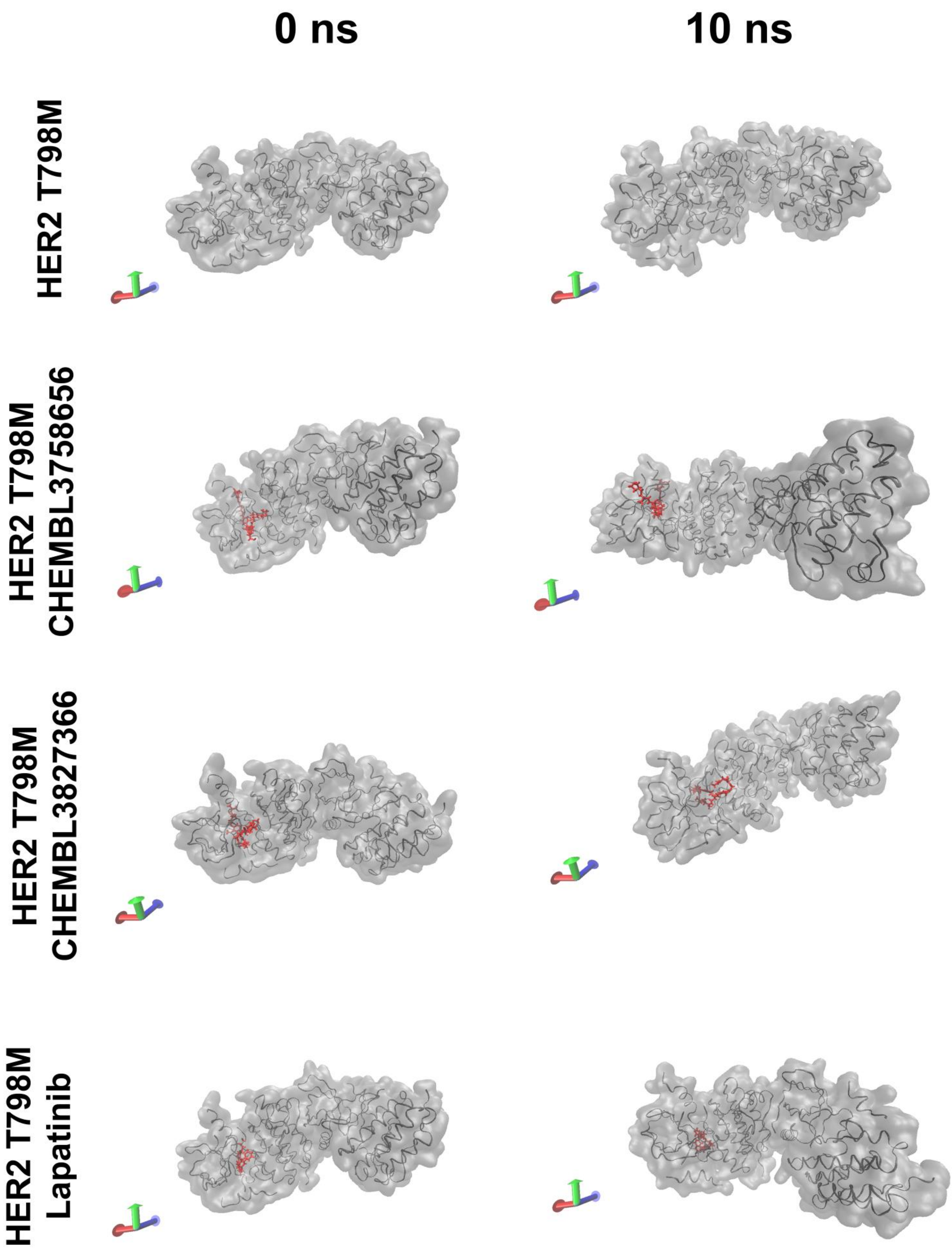

Supplementary Fig. (9). Visualization of the binding interaction of curcumin derivatives againts HER2 T798M

Comprehensive Molecular Docking and Molecular Dynamic Reveal Inhibitor of HER2 L755S, T798I, and T798M based on Large Database of Curcumin Derivatives

Mantiqa Syafa Duvadillan Gusrin[1,2], Yonika Arum Larasati[3], Rohmad Yudi Utomo[2,4]

[1]Graduate School of Master of Pharmaceutical Sciences, Faculty of Pharmacy, Universitas Gadjah Mada, Sekip Utara, Sleman, Yogyakarta Indonesia 55281;

[2]Cancer Chemoprevention Research Center, Faculty of Pharmacy, Universitas Gadjah Mada, Sekip Utara, Sleman, Yogyakarta Indonesia 55281;

[3]Translational Research Center in Oncohaematology, Department of Cell Physiology and Metabolism, Faculty of Medicine, University of Geneva, Geneva, Switzerland;

[4]Laboratory of Medicinal Chemistry, Department of Pharmaceutical Chemistry, Faculty of Pharmacy, Universitas Gadjah Mada, Sekip Utara, Sleman, Yogyakarta Indonesia 55281

*Corresponding author

Email: rohmad.yudi.utomo@ugm.ac.id

Content

Supplementary Table 1. Lipinski rule of five profile of curcumin derivatives............. 2

Supplementary Table 2. Top-100 curcumin derivatives with lower docking score toward HER2 WT... 13

Supplementary Figure 1. 26

Supplementary Figure 2. 27

Supplementary Figure 3. 28

Supplementary Figure 4. 29

Supplementary Figure 5. 30

Supplementary Figure 6. 31

Supplementary Figure 7. 32

Supplementary Figure 8. 33

Supplementary Figure 9. 34

Supplementary Table 1. Lipinski rule of five profile of curcumin derivatives

No	Smiles	SlogP	ExactMW	NumHBD	NumHBA
1	COc1cc(/C=C/C(=O)CC(=O)/C=C/c2ccc(O)cc2)ccc1F	3.7948	340.1111	1	4
2	O=C(/C=C/c1ccc(O)cc1Cl)CC(=O)/C=C/c1ccc(O)cc1Cl	4.6595	376.0269	2	4
3	COc1ccc(/C=C/C(=O)CC(=O)/C=C/c2ccc3[nH]ccc3c2)cc1O	4.137	361.1314	2	4
4	COc1cc(/C=C/C(=O)CC(=O)/C=C/c2ccc(O)c(C[N+](C)(C)C)c2)ccc1O.[I-]	0.5715	537.1012	2	5
5	COc1cc(/C=C/C(=O)C(C)(C)C(=O)/C=C/c2ccc(O)c(OC)c2)ccc1O	4.006	396.1573	2	6
6	COc1ccc(/C=C/C(=O)CC(=O)/C=C/c2cc(O)ccc2[N+](=O)[O-])cc1O	3.2695	383.1005	2	7
7	NCCOc1ccc(/C=C/C(=O)CC(=O)/C=C/c2ccc(OCCN)c(OCCN)c2)cc1OCCN	1.2915	512.2635	4	10
8	COc1cc(/C=C/C(=O)CC(=O)/C=C/c2ccc(OCCCCC[n+]3ccccc3)c(OC)c2)ccc1O.[Br-]	2.2051	595.1569	1	6
9	O=C(/C=C/c1ccc(O)c(OC(F)(F)F)c1)CC(=O)/C=C/c1ccc(O)c(OC(F)(F)F)c1	5.1499	476.0695	2	6
10	COc1ccc(/C=C/C(=O)CC(=O)/C=C/c2ccc(OC)cc2)cc1	3.9587	336.1362	0	4
11	O=C(/C=C/c1ccccc1C(F)(F)F)CC(=O)/C=C/c1ccccc1C(F)(F)F	5.9791	412.0898	0	2
12	O=C(/C=C/c1cccc(O)c1)CC(=O)/C=C/c1cccc(O)c1	3.3527	308.1049	2	4
13	COc1cc(C=C(C(=O)/C=C/c2ccc(N(C)C)cc2)C(=O)/C=C/c2ccc(N(C)C)cc2)ccc1O	5.4812	496.2362	1	6
14	COc1cc(/C=C/C(=O)CC(=O)/C=C/c2ccco2)ccc1O	3.2487	312.0998	1	5
15	COc1cccc(/C=C/C(=O)C(C)(C)C(=O)/C=C/c2cccc(OC)c2)c1	4.5948	364.1675	0	4
16	COc1cc(/C=C/C(=O)CC(=O)/C=C/c2ccc(OCCN)c(OC)c2)ccc1OCCN	2.6337	454.2104	2	8
17	COc1ccccc1/C=C/C(=O)CC(=O)/C=C/c1ccccc1OC	3.9587	336.1362	0	4

18	COc1cc(/C=C/C(=O)CC(=O)/C=C/c2ccc(OCCC[P+](c3ccccc3)(c3ccccc3)c3ccccc3)c(OC)c2)ccc1O.[Cl-]	4.4313	706.225 1	1	6
19	NCCOc1ccc(/C=C/C(=O)CC(=O)/C=C/c2ccc(OCCN)cc2)cc1	2.6165	394.189 3	2	6
20	COc1ccc(/C=C/C(=O)CC(=O)/C=C/c2c[nH]c3cccc([N+](=O)[O-])c23)cc1O	4.0452	406.116 5	2	6
21	COc1cc(C=CC(=O)CC(=O)C=Cc2ccc(O)c(OC)c2)ccc1O	3.3699	368.126	2	6
22	COc1ccc(/C=C/C(=O)CC(=O)/C=C/c2ccc(N(C)C)cc2Cl)cc1O	4.3751	399.123 7	1	5
23	CCOc1ccc(/C=C/C(=O)C(C)(C)C(=O)/C=C/c2ccc(OCC)c(OC)c2)cc1OC	5.3922	452.219 9	0	6
24	O=C(/C=C/c1ccc(O)cc1)CC(=O)/C=C/c1cc(C(F)(F)F)ccc1F	4.805	378.087 9	1	3
25	COc1cc(/C=C/C(=O)CC(=O)/C=C/c2ccc(OCCCCC[n+]3ccccc3)c(OC)c2)ccc1OCCCCC[n+]1ccccc1.[Br-].[Br-]	1.0403	822.187 9	0	6
26	COc1cc(/C=C/C(=O)CC(=O)/C=C/c2ccc(OCCO)c(OC)c2)ccc1O	3.0354	412.152 2	2	7
27	COc1cc(/C=C/C(=O)C(=Cc2ccc(N(C)C)cc2)C(=O)/C=C/c2ccc(O)c(OC)c2)ccc1O	5.1294	499.199 5	2	7
28	COc1ccc(/C=C/C(=O)C(C)(C)C(=O)/C=C/c2ccc(OC)c(OC)c2)cc1OC	4.612	424.188 6	0	6
29	COc1cc(/C=C/C(=O)CC(=O)/C=C/c2ccc(O)cc2)ccc1OC(C)=O	3.581	380.126	1	6
30	COc1cc(/C=C/C(=O)CC(=O)/C=C/c2ccc3[nH]ccc3c2)ccc1O	4.137	361.131 4	2	4
31	COc1ccc(/C=C/C(=O)CC(=O)/C=C/c2ccc(O)c(OC)c2)cc1O	3.3699	368.126	2	6
32	COc1cc(/C=C/C(=O)CC(=O)/C=C/c2ccc(OC(=O)c3ccccc3OC(C)=O)c(OC)c2)ccc1O	4.8088	530.157 7	1	9
33	O=C(/C=C/c1ccc(O)cc1)CC(=O)/C=C/c1cc(O)ccc1Br	4.1152	386.015 4	2	4
34	COc1cccc(/C=C/C(=O)CC(=O)/C=C/c2ccc(O)cc2)c1O	3.3613	338.115 4	2	5
35	CCN(CC)c1ccc(/C=C/C(=O)CC(=O)/C=C/c2ccc(O)cc2)c(OCOC)c1	4.476	423.204 6	1	6
36	COc1cc(/C=C/C(=O)CC(=O)/C=C/c2ccc3ccccc3c2)ccc1O	4.8089	372.136 2	1	4
37	COc1cc(/C=C/C(=O)CC(=O)/C=C/c2ccc(O)c(C[N+](C)(C)C)c2)ccc1O	3.5675	410.196 2	2	5

38	COc1cc(/C=C/C(=O)CC(=O)/C=C/c2ccc(CN(C)C)cc2)ccc1O	3.7173	379.178<br>4	1	5
39	COc1ccc(OC)c(/C=C/C(=O)CC(=O)/C=C/c2cc(OC)ccc2OC)c1	3.9759	396.157<br>3	0	6
40	COc1ccc(/C=C/C(=O)C(C)(C)C(=O)/C=C/c2ccc(OC)cc2)cc1	4.5948	364.167<br>5	0	4
41	COc1cc(/C=C/C(=O)CC(=O)/C=C/c2ccc(OCCN)c(OC)c2)ccc1OCCN.Cl.Cl	3.4773	526.163<br>7	2	8
42	COC(=O)/C=C/C(C(=O)/C=C/c1ccc(OC)c(OC)c1)C(=O)/C=C/c1ccc(OC)c(OC)c1	3.9312	480.178<br>4	0	8
43	COc1cc(/C=C/C(=O)CC(=O)/C=C/c2ccc(OC(=O)CNCCCl)c(OC)c2)ccc1OC(=O)CNCCCl	3.4263	606.153<br>6	2	10
44	COc1cc(/C=C/C(=O)CC(=O)/C=C/c2ccc(O)cc2)ccc1OCCCCC[n+]1ccccc1	5.1925	486.227<br>5	1	5
45	COc1cc(/C=C/C(=O)CC(=O)/C=C/c2cccnc2)ccc1O	3.0507	323.115<br>8	1	5
46	O=C(/C=C/c1ccc(O)cc1)CC(=O)/C=C/c1cc(OCc2ccccc2)ccc1Br	5.9886	476.062<br>3	1	4
47	O=C(/C=C/c1ccc(O)cc1)CC(=O)/C=C/c1cccc(Br)c1	4.4096	370.020<br>5	1	3
48	COCOc1c(O)cccc1/C=C/C(=O)CC(=O)/C=C/c1ccc(O)cc1	3.3354	368.126	2	6
49	CCOc1cc(/C=C/C(=O)CC(=O)/C=C/c2ccc(O)c(OCC)c2)ccc1O	4.1501	396.157<br>3	2	6
50	COc1cc(/C=C/C(=O)CC(=O)/C=C/c2ccc(O)c(O)c2)ccc1O	3.0669	354.110<br>3	3	6
51	COc1cc(/C=C/C(=O)CC(=O)/C=C/c2ccc(OC)c(OC)c2)ccc1O	3.6729	382.141<br>6	1	6
52	C#CCOc1ccc(/C=C/C(=O)CC(=O)/C=C/c2ccc(OCC#C)c(OC)c2)cc1OC	3.9827	444.157<br>3	0	6
53	COc1cc(/C=C/C(=O)CC(=O)/C=C/c2ccc(OC(=O)c3ccccc3O)c(OC)c2)ccc1O	4.5891	488.147<br>1	2	8
54	CCOc1cc(/C=C/C(=O)CC(=O)/C=C/c2ccc(OC(=O)CNCCCl)c(OCC)c2)ccc1OC(=O)CNCCCl	4.2065	634.184<br>9	2	10
55	CN(C)c1ccc(/C=C/C(=O)CC(=O)/C=C/c2ccc(N(C)C)cc2)cc1	4.0735	362.199<br>4	0	4
56	COc1c(O)cc(/C=C/C(=O)CC(=O)/C=C/c2ccc(O)c(O)c2)cc1O	2.7725	370.105<br>3	4	7

57	COc1cc(/C=C/C(=O)CC(=O)/C=C/c2ccc(OCCCCC[n+]3ccccc3)cc2)ccc1O.[Br-]	2.1965	565.146 4	1	5
58	COc1cc(/C=C/C(=O)CC(=O)/C=C/c2ccc(OCCOCCN)c(OC)c2)ccc1OCCOCCN	2.6669	542.262 8	2	10
59	COc1cc(/C=C/C(=O)CC(=O)/C=C/c2ccc(OCC(=O)N[C@@H](CCCCN)C(=O)O)c(OC)c2)ccc1O	2.7415	554.226 4	4	9
60	O=C(/C=C/c1ccc(O)cc1)CC(=O)/C=C/c1cn(Cc2ccccc2)c2ccccc12	5.6501	421.167 8	1	4
61	COc1ccc(OC)c(/C=C/C(=O)C(C)(C)C(=O)/C=C/c2cc(OC)ccc2OC)c1	4.612	424.188 6	0	6
62	COc1cc(/C=C/C(=O)CC(=O)/C=C/c2ccc(OCCOCCN)c(OC)c2)ccc1OCCOCCN.Cl.Cl	3.5105	614.216 2	2	10
63	O=C(/C=C/c1ccc(O)cc1)CC(=O)/C=C/c1cc(O)ccc1[N+](=O)[O-]	3.2609	353.089 9	2	6
64	COc1cc(/C=C/C(=O)CC(=O)/C=C/c2ccc(O)c(OC)c2)cc(OC)c1	3.6729	382.141 6	1	6
65	COCOc1cccc(O)c1/C=C/C(=O)CC(=O)/C=C/c1ccc(OC)c(O)c1	3.344	398.136 6	2	7
66	COc1cccc(/C=C/C(=O)CC(=O)/C=C/c2cccc(OC)c2OC)c1OC	3.9759	396.157 3	0	6
67	COc1cc(/C=C/C(=O)C(=Cc2ccncc2)C(=O)/C=C/c2ccc(O)c(OC)c2)ccc1O	4.4584	457.152 5	2	7
68	COc1ccc(/C=C/C(=O)C(C)(F)C(=O)/C=C/c2ccc(OC)c(OC)c2)cc1OC	4.314	428.163 5	0	6
69	O=C(/C=C/c1ccc(O)cc1)CC(=O)/C=C/c1c[nH]c2ccccc12	4.1284	331.120 8	2	3
70	O=C(/C=C/c1ccc(O)cc1)CC(=O)/C=C/c1ccc(O)c(Cl)c1	4.0061	342.065 9	2	4
71	Cc1cc(/C=C/C(=O)CC(=O)/C=C/c2ccc(OCCN)c(C)c2)ccc1OCCN	3.2333 4	422.220 6	2	6
72	O=C(/C=C/c1ccc(O)cc1)CC(=O)/C=C/c1ccccc1C(F)(F)F	4.6659	360.097 3	1	3
73	COC(=O)c1ccccc1/C=C/C(=O)CC(=O)/C=C/c1ccc(O)cc1	3.4337	350.115 4	1	5
74	CSc1ccccc1/C=C/C(=O)CC(=O)/C=C/c1ccc(O)cc1	4.369	338.097 7	1	4
75	O=C(/C=C/c1ccc(O)cc1)CC(=O)/C=C/c1cc(O)ccc1-c1ccccc1	5.0197	384.136 2	2	4

76	O=C(/C=C/c1ccc(O)cc1)CC(=O)/C=C/c1ccc(OC(=O)c2ccccc2)cc1	4.8663	412.1311	1	5
77	Cc1cc(/C=C/C(=O)CC(=O)/C=C/c2ccc(OCCN)c(C)c2)ccc1OCCN.Cl.Cl	4.07694	494.1739	2	6
78	COC(=O)C(C(=O)/C=C/c1ccc(O)c(OC)c1)C(=O)/C=C/c1ccc(O)c(OC)c1	2.769	426.1315	2	8
79	COc1cc(/C=C/C(=O)CC(=O)/C=C/c2ccc(O)cc2)ccc1OC(=O)c1ccccc1	4.8749	442.1416	1	6
80	[2H]c1c([2H])c(/C=C/C(=O)CC(=O)/C=C/c2ccc(O)c(OC)c2)c([2H])c([2H])c1O	3.3613	342.1405	2	5
81	COc1cc(/C=C/C(=O)CC(=O)/C=C/c2ccc(OC(C)=O)cc2)ccc1O	3.581	380.126	1	6
82	COc1cc(C=C(C(=O)/C=C/c2ccc(O)c(OC)c2)C(=O)/C=C/c2ccc(O)c(OC)c2)ccc1O	4.7776	502.1628	3	8
83	CC(C)(C(=O)/C=C/c1ccc(O)c(O)c1)C(=O)/C=C/c1ccc(O)c(O)c1	3.4	368.126	4	6
84	O=C(/C=C/c1ccc(O)c(O)c1)CC(=O)/C=C/c1cc(O)c(O)c(O)c1	2.4695	356.0896	5	7
85	COc1cc(/C=C/C(=O)CC(=O)/C=C/c2ccc(OC(=O)CCNCCCl)c(OC)c2)ccc1OC(=O)CCNCCCl	4.2065	634.1849	2	10
86	CN(C)c1ccc(/C=C/C(=O)CC(=O)/C=C/c2ccc(O)cc2)c([N+](=O)[O-])c1	3.6213	380.1372	1	6
87	O=C(/C=C/c1ccc(O)cc1)CC(=O)/C=C/c1ccc(O)cc1	3.3527	308.1049	2	4
88	COc1cc(/C=C/C(=O)C(=CO)C(=O)/C=C/c2ccc(OC(C)=O)c(OC)c2)ccc1OC(C)=O	3.8611	480.142	1	9
89	CS(=O)(=O)n1ccc2ccc(/C=C/C(=O)CC(=O)/C=C/c3ccc(O)cc3)cc21	3.4095	409.0984	1	6
90	COc1cc(/C=C/C(=O)CC(=O)/C=C/c2ccc(OCC(=O)O)c(OC)c2)ccc1O	3.1277	426.1315	2	7
91	COc1cc(/C=C/C(=O)CC(=O)/C=C/c2ccc(OCC=C(C)C)cc2)ccc1OCC=C(C)C	6.6401	474.2406	0	5
92	COc1cc(/C=C/C(=O)C(=NNc2ccccc2)C(=O)/C=C/c2ccc(O)c(OC)c2)ccc1O	4.4479	472.1634	3	8
93	Cl.Cl.Cl.Cl.NCCOc1ccc(/C=C/C(=O)CC(=O)/C=C/c2ccc(OCCN)c(OCCN)c2)cc1OCCN	2.9787	656.1702	4	10
94	CO/C(O)=C/C=C(C(=O)/C=C/c1ccc(OC)c(OC)c1)C(=O)/C=C/c1ccc(OC)c(OC)c1	4.558	480.1784	1	8
95	COc1cccc(/C=C/C(=O)CC(=O)/C=C/c2cccc(OC)c2)c1	3.9587	336.1362	0	4

96	O=C(/C=C/c1ccc(O)c(O)c1)CC(=O)/C=C/c1ccc(O)c(O)c1	2.7639	340.0947	4	6
97	CCOC(=O)C(C(=O)/C=C/c1ccc(O)c(OC(F)(F)F)c1)C(=O)/C=C/c1ccc(O)c(OC(F)(F)F)c1	4.9391	548.0906	2	8
98	CC(C)(C(=O)/C=C/c1ccc(O)cc1)C(=O)/C=C/c1ccc(O)cc1	3.9888	336.1362	2	4
99	O=C(/C=C/c1ccc(C(F)(F)F)cc1)CC(=O)/C=C/c1ccc(C(F)(F)F)cc1	5.9791	412.0898	0	2
100	COc1cc(/C=C/C(=O)CC(=O)/C=C/c2ccc(O)c(OC)c2)ccc1O	3.3699	368.126	2	6
101	COc1ccc(O)c(/C=C/C(=O)CC(=O)/C=C/c2ccc(O)cc2)c1	3.3613	338.1154	2	5
102	COc1ccc(/C=C/C(=O)C(C)C(=O)/C=C/c2ccc(OC)c(OC)c2)cc1OC	4.2219	410.1729	0	6
103	COc1cc(O)cc(/C=C/C(=O)CC(=O)/C=C/c2ccc(O)c(OC)c2)c1	3.3699	368.126	2	6
104	O=C(/C=C/c1ccc(O)cc1)CC(=O)/C=C/c1ccccc1-c1cccc2ccccc12	6.4673	418.1569	1	3
105	COc1cc(OCCN)ccc1/C=C/C(=O)CC(=O)/C=C/c1ccc(OCCN)cc1OC	2.6337	454.2104	2	8
106	COc1cc(/C=C/C(=O)CC(=O)/C=C/c2ccc3c(c2)OCO3)ccc1O	3.3844	366.1103	1	6
107	CC(C)(C)[Si](C)(C)OCc1ccccc1/C=C/C(=O)CC(=O)/C=C/c1ccc(O)cc1	6.1689	436.207	1	4
108	O=C(/C=C/c1ccc(Cl)cc1)CC(=O)/C=C/c1ccc(Cl)cc1	5.2483	344.0371	0	2
109	COc1cc(/C=C/C(=O)CC(=O)/C=C/c2ccc(N(C)C)cc2Cl)ccc1O	4.3751	399.1237	1	5
110	COc1cc(/C=C/C(=O)CC(=O)/C=C/c2ccc(O)cc2)c(Br)cc1O	4.1238	416.0259	2	5
111	Cl.Cl.NCCOc1ccc(/C=C/C(=O)CC(=O)/C=C/c2ccc(OCCN)cc2)cc1	3.4601	466.1426	2	6
112	COc1cc(/C=C/C(=O)CC(=O)/C=C/c2ccc(OC)c3ccccc23)ccc1O	4.8175	402.1467	1	5
113	N[C@@H](Cc1ccccc1)C(=O)Nc1ccc(/C=C/C(=O)CC(=O)/C=C/c2ccc(O)cc2)cc1	4.1556	454.1893	3	5
114	COc1cc(/C=C/C(=O)C(C)(C)C(=O)/C=C/c2cc(OC)c(OC)c(OC)c2)cc(OC)c1OC	4.6292	484.2097	0	8
115	COc1cc(/C=C/C(=O)CC(=O)/C=C/c2ccc(O)cc2)cc(OC)c1OC	3.6729	382.1416	1	6

116	COc1cc(/C=C/C(=O)C(=Cc2ccc3c(c2)C(=O)N(C2CCC(=O)NC2=O)C3=O)C(=O)/C=C/c2ccc(O)c(OC)c2)ccc1O	3.4646	636.1744	3	10
117	COc1ccc(O)c(/C=C/C(=O)CC(=O)/C=C/c2ccc(O)c(OC)c2)c1	3.3699	368.126	2	6
118	COC(=O)C(C(=O)/C=C/c1ccc(O)cc1)C(=O)/C=C/c1ccc(O)cc1	2.7518	366.1103	2	6
119	COC(=O)COc1ccc(/C=C/C(=O)CC(=O)/C=C/c2ccc(O)c(OC)c2)cc1OC	3.2161	440.1471	1	8
120	O=C(/C=C/c1ccc(O)cc1)CC(=O)/C=C/c1cc(O)c(Br)cc1Br	4.8777	463.9259	2	4
121	O=C(/C=C/c1ccc(O)c(O)c1)CC(=O)/C=C/c1cccc(O)c1O	2.7639	340.0947	4	6
122	O=C(/C=C/c1ccc(O)cc1)CC(=O)/C=C/c1ccc(O)cc1O	3.0583	324.0998	3	5
123	COc1cc(/C=C\C(=O)CC(=O)/C=C\c2ccc(O)c(OC)c2)ccc1O	3.3699	368.126	2	6
124	COc1cc(/C=C/C(=O)CC(=O)/C=C/c2ccc(OCC=C(C)C)c(OC)c2)ccc1O	5.0093	436.1886	1	6
125	COc1cc(N(C)C)ccc1/C=C/C(=O)CC(=O)/C=C/c1ccc(O)cc1	3.7217	365.1627	1	5
126	COc1cc(/C=C/C(=O)CC(=O)/C=C/c2ccc(O)c(CO)c2)ccc1O	2.8536	368.126	3	6
127	O=C(/C=C/c1ccc(O)cc1)C(C(=O)/C=C/c1ccc(O)cc1)C(=O)Nc1ccccc1	4.2175	427.142	3	5
128	COc1cc(/C=C/C(=O)CC(=O)/C=C/c2ccc(OC(=O)[C@@H](N)C(C)C)c(OC)c2)ccc1OC(=O)[C@@H](N)C(C)C	3.7361	566.2628	2	10
129	CN1CCN(c2ccc(/C=C/C(=O)CC(=O)/C=C/c3ccc(N4CCN(C)CC4)cc3)cc2)CC1	3.4451	472.2838	0	6
130	CN(C)Cc1ccc(/C=C/C(=O)C(C(=O)/C=C/c2ccc(N(C)C)cc2)C(=O)Nc2ccccc2)cc1	4.9339	495.2522	1	5
131	COc1cc(O)ccc1/C=C/C(=O)CC(=O)/C=C/c1ccc(O)cc1OC	3.3699	368.126	2	6
132	COc1ccc([N+](=O)[O-])c(/C=C/C(=O)CC(=O)/C=C/c2ccc(O)cc2)c1	3.5639	367.1056	1	6
133	Cc1cc(/C=C/C(=O)CC(=O)/C=C/c2ccc(O)cc2)ccc1O	3.66112	322.1205	2	4
134	O=C(/C=C/c1ccc(O)cc1)CC(=O)/C=C/c1ccc2cc[nH]c2c1	4.1284	331.1208	2	3
135	COc1ccc(/C=C/C(=O)CC(=O)/C=C/c2ccc(OCC(=O)N[C@@H](CCCCN)C(=O)O)c(OC)c2)cc1OC	3.0445	568.2421	3	9
136	CC(C)(C(=O)/C=C/c1ccc(O)c(Br)c1)C(=O)/C=C/c1ccc(O)c(Br)c1	5.5138	491.9572	2	4

137	COc1cc(/C=C/C(=O)CC(=O)/C=C/c2c[nH]c3ccccc23)ccc1O	4.137	361.1314	2	4
138	O=C(/C=C/c1cccc(O)c1)CC(=O)/C=C/c1cccc(O)c1O	3.0583	324.0998	3	5
139	O=C(/C=C/c1cccc(O)c1O)CC(=O)/C=C/c1cccc(O)c1O	2.7639	340.0947	4	6
140	COc1ccc(/C=C/C(=O)CC(=O)/C=C/c2ccc(N(C)C)c3ccccc23)cc1O	4.8749	415.1784	1	5
141	O=C(/C=C/c1ccc(O)cc1)CC(=O)/C=C/c1cccc2cccnc12	4.1953	343.1208	1	4
142	COc1ccc(/C=C/C(=O)CC(=O)/C=C/c2ccc(O)c(OC)c2)cc1	3.6643	352.1311	1	5
143	COc1ccc2ccccc2c1/C=C/C(=O)CC(=O)/C=C/c1ccc(O)cc1	4.8089	372.1362	1	4
144	CCN(CC)c1ccc(/C=C/C(=O)CC(=O)/C=C/c2ccc(O)cc2)c(OCC=C(C)C)c1	5.8383	447.241	1	5
145	COc1ccc(/C=C/C(=O)CC(=O)/C=C/c2ccc(O)cc2)c(OC)c1	3.6643	352.1311	1	5
146	COc1cc(/C=C/C(=O)C(=Cc2ccc(F)cc2)C(=O)/C=C/c2ccc(O)c(OC)c2)ccc1O	5.2025	474.1479	2	6
147	CC(CO)(CO)C(=O)Oc1ccc(/C=C/C(=O)C(C)(C)C(=O)/C=C/c2ccc(OC(=O)C(C)(CO)CO)cc2)cc1	2.3702	568.2308	4	10
148	CC(=O)O[C@H]1C2=C(C([N+]#N)=C3C(=O)C4C(=O)C=CC=C4C([O-])=C32)[C@@H](OC(C)=O)[C@@](C)(O)[C@@H]1OC(C)=O	-0.15792	496.1118	1	11
149	C=CCOc1ccc(/C=C/C(=O)CC(=O)/C=C/c2ccc(OCC=C)c(OC)c2)cc1OC	5.0883	448.1886	0	6
150	O=C(/C=C/c1c[nH]c2ccccc12)CC(=O)/C=C/c1c[nH]c2ccccc12	4.9041	354.1368	2	2
151	COc1cccc(OC)c1/C=C/C(=O)C(C)(C)C(=O)/C=C/c1c(OC)cccc1OC	4.612	424.1886	0	6
152	COc1cc(/C=C/C(=O)CC(=O)/C=C/c2cccc(OC)c2O)ccc1O	3.3699	368.126	2	6
153	COc1cc(/C=C/C(=O)CC(=O)/C=C/c2ccc(OC(C)=O)cc2)ccc1OC(C)=O	3.8007	422.1366	0	7
154	O=C(/C=C/c1ccco1)C(C(=O)/C=C/c1ccco1)=C1SCCCS1	4.8191	372.049	0	6
155	COc1cc(/C=C/C(=O)CC(=O)/C=C/c2ccc(C(C)=O)c(OC)c2)ccc1C(C)=O	4.3639	420.1573	0	6
156	O=C(/C=C/c1ccc(O)cc1)CC(=O)/C=C/c1ccccc1-c1ccccc1	5.3141	368.1412	1	3

157	O=C(/C=C/c1ccc(O)cc1)CC(=O)/C=C/c1ccccc1[N+](=O)[O-]	3.5553	337.095	1	5
158	O=C(/C=C/c1cc(O)ccc1[N+](=O)[O-])CC(=O)/C=C/c1cc(O)ccc1[N+](=O)[O-]	3.1691	398.075	2	8
159	CC1(C)C(=O)/C=C\C(=O)C(C)(C)C(=O)/C=C\C(=O)C(C)(C)c2ccc(o2)C(C)(C)C(=O)/C=C\C1=O	3.984	480.2148	0	7
160	COc1ccccc1/C=C/C(=O)C(C)(C)C(=O)/C=C/c1ccccc1OC	4.5948	364.1675	0	4
161	COc1ccc(/C=C/C(=O)CC(=O)/C=C/c2ccc(OC)c(OC)c2)cc1OC	3.9759	396.1573	0	6
162	CCCOc1ccc(/C=C/C(=O)CC(=O)/C=C/c2ccc(OCCC)c(OC)c2)cc1OC	5.5363	452.2199	0	6
163	N[C@H](Cc1ccccc1)C(=O)Nc1ccc(/C=C/C(=O)CC(=O)/C=C/c2ccc(O)cc2)cc1	4.1556	454.1893	3	5
164	COc1ccc(/C=C/C(=O)CC(=O)/C=C/c2ccc(OC)c(O)c2)cc1O	3.3699	368.126	2	6
165	COc1cc(/C=C/C(=O)CC(=O)/C=C/c2ccc(OCCO)c(OC)c2)ccc1OCCO	2.7009	456.1784	2	8
166	O=C(/C=C/c1ccccc1O)CC(=O)/C=C/c1cccc(O)c1O	3.0583	324.0998	3	5
167	O=C(/C=C/c1cccc(O)c1)CC(=O)/C=C/c1cc(O)ccc1O	3.0583	324.0998	3	5
168	COC(=O)CCC(C(=O)/C=C/c1ccc([O-])c(OC(F)(F)F)c1)C(=O)/C=C/c1ccc([O-])c(OC(F)(F)F)c1.[Na+].[Na+]	-1.9268	606.0701	0	8
169	CC(C)(C(=O)/C=C/c1ccccc1)C(=O)/C=C/c1ccccc1	4.5776	304.1463	0	2
170	COc1cc(/C=C/C(=O)CC(=O)/C=C/c2ccc(O)cc2)ccc1O	3.3613	338.1154	2	5
171	COc1cc(OCC2CCCO2)ccc1/C=C/C(=O)CC(=O)/C=C/c1ccc2cc[nH]c2c1	4.9892	445.1889	1	5
172	COc1cc(/C=C/C(=O)CC(=O)/C=C/c2ccc(OC(C)=O)c(OC)c2)ccc1O	3.5896	410.1366	1	7
173	COc1cc(O)ccc1/C=C/C(=O)CC(=O)/C=C/c1ccc2cc[nH]c2c1	4.137	361.1314	2	4
174	COc1ccc(/C=C/C(=O)CC(=O)/C=C/c2ccc(N(C)C)cc2[N+](=O)[O-])cc1O	3.6299	410.1478	1	7
175	CO[13c]1[13cH][13c](/C=C/C(=O)CC(=O)/C=C/[13c]2[13cH][13cH][13c](O)[13c](OC)[13cH]2)[13cH][13cH][13c]1O	3.3699	380.1662	2	6
176	O=C(/C=C/c1cc(O)c(O)c(O)c1)CC(=O)/C=C/c1ccccc1O	2.7639	340.0947	4	6

177	O=C(/C=C/c1ccc(O)c(O)c1)CC(=O)/C=C/c1cc(O)ccc1O	2.7639	340.0947	4	6
178	COc1cc(/C=C/C(=O)CC(=O)/C=C/c2ccc(O)c(C(F)(F)F)c2)ccc1O	4.3801	406.1028	2	5
179	COc1ccc(/C=C/C(=O)C(=Cc2ccc(OC)c(OC)c2)C(=O)/C=C/c2ccc(OC)cc2)cc1	5.6694	484.1886	0	6
180	COc1cc(/C=C/C(=O)C(=Cc2cccc(F)c2)C(=O)/C=C/c2ccc(O)c(OC)c2)ccc1O	5.2025	474.1479	2	6
181	O=C(/C=C/c1cccc(O)c1)CC(=O)/C=C/c1ccc(O)c(O)c1	3.0583	324.0998	3	5
182	COc1ccc(/C=C/C(=O)CC(=O)/C=C/c2ccc(OC)c(OC)c2)cc1	3.9673	366.1467	0	5
183	COc1cc(/C=C/C(=O)CC(=O)/C=C/c2ccc(OC(=O)c3ccccc3)c(OC)c2)ccc1O	4.8835	472.1522	1	7
184	O=C(/C=C/c1ccc(O)cc1)CC(=O)/C=C/c1ccccc1-c1ccc2ccccc2c1	6.4673	418.1569	1	3
185	COc1cc(/C=C/C(=O)CC(=O)/C=C/c2ccc(O)c(O)c2)cc(OC)c1OC	3.3785	398.1366	2	7
186	COc1cc(/C=C/C(=O)CC(=O)/C=C/c2ccc(O)cc2)ccc1OCCCCC[n+]1ccccc1.[Br-]	2.1965	565.1464	1	5
187	COc1cc(/C=C/C(=O)C(Cc2ccc(O)c(OC)c2)C(=O)/C=C/c2ccc(O)c(OC)c2)ccc1O	4.5529	504.1784	3	8
188	O=C(/C=C/c1cccc([N+](=O)[O-])c1)CC(=O)/C=C/c1ccc(O)c(O)c1	3.2609	353.0899	2	6
189	CC1(C)C(=O)/C=C\C(=O)C(C)(C)C(=O)/C=C\C(=O)C(C)(C)C(=O)/C=C\C(=O)C(C)(C)C(=O)/C=C\C1=O	2.8824	496.2097	0	8
190	CCO/C(O)=C/C=C(C(=O)/C=C/c1ccc(OC)c(OC)c1)C(=O)/C=C/c1ccc(OC)c(OC)c1	4.9481	494.1941	1	8
191	CN(C)Cc1ccc(/C=C/C(=O)CC(=O)/C=C/c2ccc(N(C)C)cc2)cc1	4.0691	376.2151	0	4
192	O=C(/C=C/c1ccc(O)cc1)CC(=O)/C=C/c1cc(F)cc(F)c1	3.9253	328.0911	1	3
193	COc1cc(/C=C/C(=O)CC(=O)/C=C/c2cc(O)ccc2OC)ccc1O	3.3699	368.126	2	6
194	COc1cc(/C=C/C(=O)CC(=O)/C=C/c2ccc(OC(C)=O)c(OC)c2)ccc1OC(C)=O	3.8093	452.1471	0	8
195	COc1cc(/C=C/C(=O)CC(=O)/C=C/c2ccccc2)ccc1O	3.6557	322.1205	1	4

196	COc1cc(/C=C/C(=O)CC(=O)/C=C/c2ccc3cc[nH]c3c2)ccc1O	4.137	361.1314	2	4
197	COc1ccc(/C=C/C(=O)CC(=O)/C=C/c2ccc3cc[nH]c3c2)c(OC)c1	4.44	375.1471	1	4
198	COc1cc(/C=C/C(=O)CC(=O)/C=C/c2ccc(OC(=O)CCl)c(OC)c2)ccc1OC(=O)CCl	4.2471	520.0692	0	8
199	CC(C)(C(=O)/C=C/c1ccccc1O)C(=O)/C=C/c1ccccc1O	3.9888	336.1362	2	4
200	CCN(CC)c1ccc(/C=C/C(=O)CC(=O)/C=C/c2ccc(O)cc2)c(OC(C)C)c1	5.2805	421.2253	1	5
201	COc1ccc(/C=C/C(=O)CC(=O)/C=C/c2cccc(O)c2OC)cc1O	3.3699	368.126	2	6
202	COc1ccc(/C=C/C(=O)CC(=O)/C=C/c2ccc(O)c(OC)c2)c(O)c1	3.3699	368.126	2	6
203	COc1cc(/C=C/C(=O)CC(=O)/C=C/c2ccc(O)cc2OC)ccc1O	3.3699	368.126	2	6
204	COc1cc(OCc2ccccc2)ccc1/C=C/C(=O)CC(=O)/C=C/c1ccc2cc[nH]c2c1	6.0104	451.1784	1	4
205	COc1cc(OCc2ccccn2)ccc1/C=C/C(=O)CC(=O)/C=C/c1ccc2cc[nH]c2c1	5.4054	452.1736	1	5
206	CCc1ccc(C=C(C(=O)/C=C/c2ccc(O)c(OC)c2)C(=O)/C=C/c2ccc(O)c(OC)c2)cc1	5.6258	484.1886	2	6
207	COc1ccc(/C=C/C(=O)CC(=O)/C=C/c2cc(O)ccc2O)cc1O	3.0669	354.1103	3	6
208	CC1=C(/C=C/C(=O)/C(=C\c2ccc(O)cc2)C(=O)/C=C/c2ccc(O)cc2)C(C)(C)CCC1	6.4156	442.2144	2	4
209	COc1ccccc1/C=C/C(=O)CC(=O)/C=C/c1ccc(O)c(O)c1	3.3613	338.1154	2	5
210	CCOc1cc(/C=C/C(=O)C(=Cc2ccccc2)C(=O)/C=C/c2ccc(O)c(OCC)c2)ccc1O	5.8436	484.1886	2	6
211	O=C(/C=C/c1cc(O)ccc1O)CC(=O)/C=C/c1cc(O)ccc1O	2.7639	340.0947	4	6
212	CCCCCOc1ccc(/C=C/C(=O)CC(=O)/C=C/c2ccc(O)c(OC)c2)cc1OC	5.2333	438.2042	1	6
213	COc1cc(/C=C/C(=O)CC(=O)/C=C/c2c(O)cccc2OC)ccc1O	3.3699	368.126	2	6
214	COC(=O)COc1ccc(/C=C/C(=O)CC(=O)/C=C/c2ccc(OC)c(OC)c2)cc1OC	3.5191	454.1628	0	8
215	O=C(/C=C/c1ccc(O)cc1)CC(=O)/C=C/c1cc(OCc2ccccc2)ccc1[N+](=O)[O-]	5.1343	443.1369	1	6

216	O=C(/C=C/c1ccc(O)c(O)c1)CC(=O)/C=C/c1ccccc1O	3.0583	324.0998	3	5
217	COc1cc(/C=C/C(=O)C(=Cc2ccccc2O)C(=O)/C=C/c2ccc(O)c(OC)c2)ccc1O	4.769	472.1522	3	7
218	COc1cc(/C=C/C(=O)CC(=O)/C=C/c2ccc(OCCCCC[n+]3ccccc3)cc2)ccc1O	5.1925	486.2275	1	5
219	O=C(/C=C/c1ccccc1O)CC(=O)/C=C/c1ccccc1O	3.3527	308.1049	2	4
220	CC1(C)C(=O)C=CC(=O)C(C)(C)C(=O)C=CC(=O)C(C)(C)c2ccc(o2)C(C)(C)C(=O)C=CC1=O	3.984	480.2148	0	7
221	COc1ccc(/C=C/C(=O)CC(=O)/C=C/c2ccc(O)cc2Cl)cc1O	4.0147	372.0765	2	5
222	CC(C)(C(=O)/C=C/c1ccc(Cl)cc1)C(=O)/C=C/c1ccc(Cl)cc1	5.8844	372.0684	0	2
223	O=C(/C=C/c1ccc(O)cc1)CC(=O)/C=C/c1cc(Cl)cc(Cl)c1O	4.6595	376.0269	2	4
224	CCN(CC)c1ccc(/C=C/C(=O)CC(=O)/C=C/c2ccc(O)cc2)c(OCc2ccccc2)c1	6.0723	469.2253	1	5
225	O=C(/C=C/c1ccc(O)cc1)CC(=O)/C=C/c1cc(Cl)ccc1[N+](=O)[O-]	4.2087	371.0561	1	5
226	O=C(/C=C/c1ccc(Br)cc1)CC(=O)/C=C/c1ccc(Br)cc1	5.4665	431.9361	0	2
227	COc1cc(/C=C/C(=O)C(=Cc2ccccc2)C(=O)/C=C/c2ccc(O)c(OC)c2)ccc1O	5.0634	456.1573	2	6
228	COc1ccc(/C=C/C(=O)CC(=O)/C=C/c2ccc(O)cc2OC)cc1O	3.3699	368.126	2	6
229	COc1cc(OCCN)ccc1/C=C/C(=O)CC(=O)/C=C/c1ccc(OCCN)cc1OC.Cl.Cl	3.4773	526.1637	2	8
230	COc1cc(/C=C/C(=O)C(C(=O)/C=C/c2ccc(O)c(OC)c2)C(=O)Nc2ccccc2)ccc1O	4.2347	487.1631	3	7
231	O=C(/C=C/c1ccc(O)cc1)CC(=O)/C=C/c1ccc2ccn(Cc3ccccc3)c2c1	5.6501	421.1678	1	4
232	COc1cc(/C=C/C(=O)CC(=O)/C=C/c2ccc(O)c(CN(C)C)c2)ccc1O	3.4229	395.1733	2	6
233	O=C(/C=C/c1ccc(O)cc1)CC(=O)/C=C/c1c[nH]c2cccc([N+](=O)[O-])c12	4.0366	376.1059	2	5
234	COc1cc(/C=C/C(=O)CC(=O)/C=C/c2ccc(F)c(OC)c2)ccc1O	3.8034	370.1217	1	5

235	COc1cc(/C=C/C(=O)CC(=O)/C=C/c2cc(OC)c(O)c(OC)c2)cc(OC)c1O	3.3871	428.1471	2	8
236	COc1cc(/C=C/C(=O)CC(=O)/C=C/c2cc(F)c(O)c(OC)c2)cc(F)c1O	3.6481	404.1071	2	6
237	COc1cc(/C=C/C(=O)C(=Cc2ccc(O)cc2)C(=O)/C=C/c2ccc(O)c(OC)c2)ccc1O	4.769	472.1522	3	7
238	CC(=O)Oc1ccc(/C=C/C(=O)CC(=O)/C=C/c2ccc(O)cc2)cc1	3.5724	350.1154	1	5
239	COc1cc(/C=C/C(=O)CC(=O)/C=C/c2cc(OC)c(O)c([N+](=O)[O-])c2)ccc1O	3.2781	413.1111	2	8
240	COCOc1cc(O)ccc1/C=C/C(=O)CC(=O)/C=C/c1ccc(O)cc1	3.3354	368.126	2	6
241	COc1cc(/C=C/C(=O)CC(=O)/C=C/c2ccc(OCCO)cc2)ccc1O	3.0268	382.1416	2	6
242	COc1ccc(/C=C/C(=O)CC(=O)/C=C/c2ccc(O)cc2)cc1	3.6557	322.1205	1	4
243	COc1cc(/C=C/C(=O)CC(=O)/C=C/c2ccc(OC(=O)c3ccccc3)cc2)ccc1O	4.8749	442.1416	1	6
244	CC(=O)Oc1ccc(/C=C/C(=O)CC(=O)/C=C/c2ccc(OC(C)=O)c(O)c2)cc1	3.4977	408.1209	1	7
245	O=C(/C=C/c1ccc(O)cc1)CC(=O)/C=C/c1ccc(O)c(O)c1	3.0583	324.0998	3	5
246	COCOc1ccc(OC)cc1/C=C/C(=O)CC(=O)/C=C/c1cc(OC)ccc1O	3.647	412.1522	1	7
247	O=C(/C=C/c1ccccc1O)CC(=O)/C=C/c1cc(O)ccc1O	3.0583	324.0998	3	5
248	COc1ccc(/C=C/C(=O)CC(=O)/C=C/c2c(O)cccc2OC)cc1O	3.3699	368.126	2	6
249	O=C(/C=C/c1cccc2cc[nH]c12)CC(=O)/C=C/c1cccc2cc[nH]c12	4.9041	354.1368	2	2
250	COc1cccc(/C=C/C(=O)CC(=O)/C=C/c2ccc(O)c(O)c2)c1	3.3613	338.1154	2	5
251	COc1cc(/C=C/C(=O)CC(=O)/C=C/c2ccc(OC(=O)C(N)CC[Se]C)c(OC)c2)ccc1O	3.4577	547.1109	2	8
252	O=C(/C=C/c1ccc(O)cc1)CC(=O)/C=C/c1ccc(O)cc1Cl	4.0061	342.0659	2	4
253	COc1ccc(/C=C/C(=O)CC(=O)/C=C/c2cccc(Oc3ccccc3)c2)cc1O	5.448	414.1467	1	5
254	COc1ccc(/C=C/C(=O)C(=CO)C(=O)/C=C/c2ccc(OC)cc2)cc1	4.0105	364.1311	1	5

255	O=C(/C=C/c1ccc(OCc2ccccc2)cc1)CC(=O)/C=C/c1ccc(OCc2ccccc2)cc1	7.0995	488.1988	0	4
256	COCOc1cccc(O)c1/C=C/C(=O)CC(=O)/C=C/c1ccc(O)c(OC)c1	3.344	398.1366	2	7
257	COc1ccc(NN=C(C(=O)/C=C/c2ccc(O)c(OC)c2)C(=O)/C=C/c2ccc(O)c(OC)c2)cc1	4.4565	502.174	3	9
258	COc1cc(/C=C/C(=O)CC(=O)/C=C/c2ccc(OC(=O)[C@@H](N)C(C)C)c(OC)c2)ccc1O	3.553	467.1944	2	8
259	O=C(/C=C/c1ccc2c(c1)OCO2)CC(=O)/C=C/c1ccc2c(c1)OCO2	3.3989	364.0947	0	6
260	COc1ccc(/C=C/C(=O)CC(=O)/C=C/c2ccc(O)c(O)c2)cc1	3.3613	338.1154	2	5
261	COc1cc(/C=C/C(=O)CC(=O)/C=C/c2ccc(O)cc2Cl)ccc1O	4.0147	372.0765	2	5
262	O=C(/C=C/c1ccccc1)CC(=O)/C=C/c1ccccc1	3.9415	276.115	0	2
263	C=CC(=O)NC(c1ccccc1)C(C(=O)/C=C/c1cccc(O)c1)C(=O)/C=C/c1cccc(O)c1	4.6223	467.1733	3	5
264	O=C(/C=C/c1ccc(O)cc1)CC(=O)/C=C/c1cccc(O)c1O	3.0583	324.0998	3	5
265	O=C(/C=C/c1ccc(O)cc1)CC(=O)/C=C/c1cc(O)ccc1O	3.0583	324.0998	3	5
266	COc1cc(/C=C/C(=O)CC(=O)/C=C/c2cccc(O)c2OC)ccc1O	3.3699	368.126	2	6
267	O=C(/C=C/c1ccc(O)c([N+](=O)[O-])c1)CC(=O)/C=C/c1ccc(O)c([N+](=O)[O-])c1	3.1691	398.075	2	8
268	COc1cccc(C=C(C(=O)/C=C/c2ccc(O)c(OC)c2)C(=O)/C=C/c2ccc(O)c(OC)c2)c1	5.072	486.1679	2	7
269	COc1ccc(/C=C/C(=O)C2(C(=O)/C=C/c3ccc(OC)c(OC)c3)CCCCC2)cc1OC	5.5363	464.2199	0	6
270	COc1cc(/C=C/C(=O)CC(=O)/C=C/c2cc(C)c(O)c(OC)c2)cc(C)c1O	3.98674	396.1573	2	6
271	CCCN(CCC)C(=O)c1cccc(/C=C/C(=O)CC(=O)/C=C/c2ccc(O)cc2)c1	4.9093	419.2097	1	4
272	COc1cc(/C=C/C(=O)CC(=O)/C=C/c2cc(O)ccc2[N+](=O)[O-])ccc1O	3.2695	383.1005	2	7
273	O=C(/C=C/c1ccc(O)c(Br)c1)CC(=O)/C=C/c1ccc(O)c(Br)c1	4.8777	463.9259	2	4
274	COc1cc(/C=C/C(=O)CC(=O)/C=C/c2cccc3ccccc23)ccc1O	4.8089	372.1362	1	4
275	CCOC(=O)CCC(F)(C(=O)/C=C/c1ccc(O)c(OC)c1)C(=O)/C=C/c1ccc(O)c(OC)c1	4.0314	486.169	2	8

276	COc1cc(O)cc(/C=C/C(=O)CC(=O)/C=C/c2cc(O)cc(OC)c2)c1	3.3699	368.126	2	6
277	COc1ccc(/C=C/C(=O)C(=CO)C(=O)/C=C/c2ccc(OC)c(OC)c2)cc1OC	4.0277	424.152 2	1	7
278	Cc1ccc(/C=C/C(=O)CC(=O)/C=C/c2ccc(C)cc2)cc1	4.5583 4	304.146 3	0	2
279	COc1cc(/C=C/C(=O)CC(=O)/C=C/c2ccc(OC(=O)CN)c(OC)c2)ccc1OC(=O)CN	1.6869	482.168 9	2	10
280	C=CCOc1ccc(/C=C/C(=O)CC(=O)/C=C/c2ccc(O)c(OC)c2)cc1OC	4.2291	408.157 3	1	6
281	O=C(/C=C/c1cccc([N+](=O)[O-])c1)CC(=O)/C=C/c1cccc([N+](=O)[O-])c1	3.7579	366.085 2	0	6
282	O=C(/C=C/c1cc(O)ccc1O)CC(=O)/C=C/c1cccc(O)c1O	2.7639	340.094 7	4	6
283	O=C(/C=C/c1ccc(O)c(OC(F)(F)F)c1)C(CCOCCO)C(=O)/C=C/c1ccc(O)c(OC(F)(F)F)c1	4.775	564.121 9	3	8
284	O=C(/C=C/c1cc(O)c(O)c(O)c1)CC(=O)/C=C/c1cc(O)c(O)c(O)c1	2.1751	372.084 5	6	8
285	CC(=O)Oc1ccc(/C=C/C(=O)CC(=O)/C=C/c2ccc(OC(C)=O)cc2)cc1	3.7921	392.126	0	6
286	COc1ccc(/C=C/C(=O)CC(=O)/C=C/c2cc(OC)c(OC)c(OC)c2)cc1O	3.6815	412.152 2	1	7
287	O=C(/C=C/c1ccc(O)cc1)CC(=O)/C=C/c1cc(OCc2ccccc2)ccc1-c1ccccc1	6.8931	474.183 1	1	4
288	O=C(/C=C/c1ccccc1)C(Cc1ccccc1)(Cc1ccccc1)C(=O)/C=C/c1ccccc1	7.0232	456.208 9	0	2
289	COc1cc(/C=C/C(=O)CC(=O)/C=C/c2cc(OC)cc(OC)c2)cc(OC)c1	3.9759	396.157 3	0	6
290	O=C(/C=C/c1ccco1)CC(=O)/C=C/c1ccco1	3.1275	256.073 6	0	4
291	CNc1ccc(/C=C/C(=O)CC(=O)/C=C/c2ccc(NC)c(C(F)(F)F)c2)cc1C(F)(F)F	6.0625	470.142 9	2	4
292	CN(C)c1ccc(/C=C/C(=O)CC(=O)/C=C/c2ccc(O)cc2)c(Cl)c1	4.3665	369.113 2	1	4
293	COc1ccc(/C=C/C(=O)C(F)(F)C(=O)/C=C/c2ccc(OC)c(OC)c2)cc1OC	4.2211	432.138 4	0	6
294	COc1ccc(/C=C/C(=O)C(=Cc2ccc(C)o2)C(=O)/C=C/c2ccc(OC)c(OC)c2)cc1OC	5.5708 2	488.183 5	0	7
295	COC(=O)CC(C(=O)/C=C/c1ccc(O)c(OC(F)(F)F)c1)C(=O)/C=C/c1ccc(O)c(OC(F)(F)F)c1	4.9391	548.090 6	2	8

296	COc1cc(/C=C/C(=O)C(=Cc2ccc(O)c(O)c2)C(=O)/C=C/c2ccc(O)c(OC)c2)ccc1O	4.4746	488.147<br>1	4	8
297	CCOC(=O)CCC(C(=O)/C=C/c1ccc(O)c(OC)c1)C(=O)/C=C/c1ccc(O)c(OC)c1	3.9393	468.178<br>4	2	8
298	CCOC(=O)/C=C/C(C(=O)/C=C/c1ccc(OC)c(OC)c1)C(=O)/C=C/c1ccc(OC)c(OC)c1	4.3213	494.194<br>1	0	8
299	COc1ccc(/C=C/C(=O)C(/C=C/C(N)=O)C(=O)/C=C/c2ccc(OC)c(OC)c2)cc1OC	3.2435	465.178<br>8	1	7
300	N#Cc1cccc(/C=C/C(=O)CC(=O)/C=C/c2ccc(O)c(O)c2)c1	3.2243<br>8	333.100<br>1	2	5
301	CC(C(=O)/C=C/c1ccc(O)c(OC(F)(F)F)c1)C(=O)/C=C/c1ccc(O)c(OC(F)(F)F)c1	5.3959	490.085<br>1	2	6
302	CCCOc1ccc(/C=C/C(=O)CC(=O)/C=C/c2ccc(O)c(OC)c2)cc1OC	4.4531	410.172<br>9	1	6
303	COc1cc(/C=C/C(=O)CC(=O)/C=C/c2ccc(N(C)C)cc2[N+](=O)[O-])ccc1O	3.6299	410.147<br>8	1	7
304	COc1ccc(/C=C/C(=O)CC(=O)/C=C/c2ccc(OC)cc2OC)c(OC)c1	3.9759	396.157<br>3	0	6
305	CCOC(O)=C(C(=O)/C=C/c1ccc(O)c(OC)c1)C(=O)/C=C/c1ccc(O)c(OC)c1	3.7859	440.147<br>1	3	8
306	COc1cc(/C=C/C(=O)CC(=O)/C=C/c2cncc(C(F)(F)F)c2)ccc1O	4.0695	391.103<br>1	1	5
307	COc1cc(/C=C/C(=O)CC(=O)/C=C/c2ccc(OC(=O)CN)c(OC)c2)ccc1O	2.5284	425.147<br>5	2	8
308	COc1ccc(O)cc1/C=C/C(=O)CC(=O)/C=C/c1cc(O)ccc1OC	3.3699	368.126	2	6
309	CCOC(=O)CCC(C(=O)/C=C/c1ccc(OC)c(OC)c1)C(=O)/C=C/c1ccc(OC)c(OC)c1	4.5453	496.209<br>7	0	8
310	O=C(/C=C/c1cccc(C(F)(F)F)c1)CC(=O)/C=C/c1cccc(C(F)(F)F)c1	5.9791	412.089<br>8	0	2
311	CCO/C(O)=C/C=C(C(=O)/C=C/c1ccc(O)c(OC)c1)C(=O)/C=C/c1ccc(O)c(OC)c1	4.3421	466.162<br>8	3	8
312	COc1ccc(/C=C/C(=O)C(/C=C/CO)C(=O)/C=C/c2ccc(OC)c(OC)c2)cc1OC	3.7505	452.183<br>5	1	7
313	COc1cc(/C=C/C(=O)C(=NNc2ccc(C)cc2)C(=O)/C=C/c2ccc(O)c(OC)c2)ccc1O	4.7563<br>2	486.179<br>1	3	8
314	CC(C)(C(=O)/C=C/c1ccccc1C(F)(F)F)C(=O)/C=C/c1ccccc1C(F)(F)F	6.6152	440.121<br>1	0	2

315	COc1ccc(/C=C/C(=O)C(=Cc2ccc(O)c(OC)c2)C(=O)/C=C/c2ccc(OC)cc2)cc1	5.3664	470.1729	1	6
316	COC(=O)CCC(C(=O)/C=C/c1ccc(O)c(OC(F)(F)F)c1)C(=O)/C=C/c1ccco1	4.318	452.1083	1	7
317	COc1cc(/C=C/C(=O)C(=NNc2ccc([N+](=O)[O-])cc2)C(=O)/C=C/c2ccc(O)c(OC)c2)ccc1O	4.3561	517.1485	3	10

Supplementary table 2. Top-100 curcumin derivatives with lower docking score toward HER2 WT

No	Chembl ID	SMILES	Docking Score
1	CHEMBL1077035	O=C(O)C(NC(=O)c1ccc(NCc2nc3C(=O)NC(N)=Nc3nc2)cc1)CCC(=O)OCCC(C(=O)/C=C/c1cc(OC)c(O)cc1)C(=O)/C=C/c1cc(OC)c(O)cc1	-11.464
2	CHEMBL3598000	O=C(/C=C/c1cc(OC)c(OC(=O)c2c(Nc3c(C)c(C)ccc3)cccc2)cc1)CC(=O)/C=C/c1cc(OC)c(O)cc1	-8.9509
3	CHEMBL450381	O=C(/C=C/c1cc(O)c(O[C@H]2[C@H](O)[C@@H](O)[C@H](O)[C@@H](CO)O2)cc1)CC(=O)/C=C/c1cc(O)c(O[C@H]2[C@H](O)[C@@H](O)[C@H](O)[C@@H](CO)O2)cc1	-9.70886
4	CHEMBL3598016	O=C(Oc1c(OC)cc(/C=C/C(=O)CC(=O)/C=C/c2cc(OC)c(OC(=O)CCc3oc(c(-c4ccccc4)n3)-c3ccccc3)cc2)cc1)CCc1oc(c(-c2ccccc2)n1)-c1ccccc1	-8.70176
5	CHEMBL3598018	O=C(/C=C/c1cc(OC)c(OC(=O)c2c(OC(=O)c3c(O)cccc3)cccc2)cc1)CC(=O)/C=C/c1cc(OC)c(OC(=O)c2c(OC(=O)c3c(O)cccc3)cccc2)cc1	-10.6294
6	CHEMBL3558376	O=C(/C=C/c1cc(OC)c(OCCCCC[n+]2ccccc2)cc1)CC(=O)/C=C/c1cc(OC)c(O)cc1	-9.15451
7	CHEMBL4802943	[P+](CCCOc1c(OC)cc(/C=C/C(=O)CC(=O)/C=C/c2cc(OC)c(O)cc2)cc1)(c1ccccc1)(c1ccccc1)c1ccccc1	-8.94341
8	CHEMBL3597993	O=C(/C=C/c1cc(OC)c(OC(=O)c2c(OC(=O)C)cccc2)cc1)CC(=O)/C=C/c1cc(OC)c(O)cc1	-8.87415
9	CHEMBL3758528	O=C(/C=C/c1cc(OC)c(OC/C=C(\C)/C)cc1)C(C(=O)/C=C/c1cc(OC)c(OC/C=C(\C)/C)cc1)C/C=C(\C)/C	-9.10491
10	CHEMBL2064003	O=C(/C(/C(=O)/C=C/c1c(OC)ccc(OC)c1)=C\c1cc(OC)c(O)c(OC)c1)/C=C/c1c(OC)ccc(OC)c1	-8.96421
11	CHEMBL2260090	ClCCNCC(=O)Oc1c(OC)cc(/C=C/C(=O)CC(=O)/C=C/c2cc(OC)c(OC(=O)CNCCCl)cc2)cc1	-9.33683
12	CHEMBL3758791	O=C(/C=C/c1cc(OC)c(OC/C=C(\CC/C=C(\C)/C)/C)cc1)CC(=O)/C=C/c1cc(OC)c(O)cc1	-9.13588
13	CHEMBL2260076	O=C(Oc1c(OC)cc(/C=C/C(=O)CC(=O)/C=C/c2cc(OC)c(OC(=O)CCNc3sc(C)nn3)cc2)cc1)CCNc1sc(C)nn1	-10.1987
14	CHEMBL3598015	O=C(Oc1c(OC)cc(/C=C/C(=O)CC(=O)/C=C/c2cc(OC)c(OC(=O)[C@H](C)c3cc4c(cc(OC)cc4)cc3)cc2)cc1)[C@H](C)c1cc2c(cc(OC)cc2)cc1	-9.37912
15	CHEMBL2063999	O=C(/C(/C(=O)/C=C/c1c(OC)ccc(OC)c1)=C\c1cc(OC)c(O)cc1)/C=C/c1c(OC)ccc(OC)c1	-9.05625
16	CHEMBL2064107	Brc1c(O)c(OC)cc(/C=C(\C(=O)/C=C/c2c(OC)cc(OC)cc2OC)/C(=O)/C=C/c2c(OC)cc(OC)cc2OC)c1	-9.42508
17	CHEMBL2260081	ClCCNCC(=O)Oc1c(OCC)cc(/C=C/C(=O)CC(=O)/C=C/c2cc(OCC)c(OC(=O)CNCCCl)cc2)cc1	-9.54031

18	CHEMBL1088293	O=C(/C=C/c1ccc(OC(=O)c2ccccc2)cc1)CC(=O)/C=C/c1ccc(OC(=O)c2ccccc2)cc1	-8.97708
19	CHEMBL4300150	O=C(/C=C/c1cc(OC)c(OCCOCCN)cc1)CC(=O)/C=C/c1cc(OC)c(OCCOCCN)cc1	-9.18416
20	CHEMBL2259878	O=C(Oc1c(OC)cc(/C=C/C(=O)CC(=O)/C=C/c2cc(OC)c(OC(=O)CCCCCC)cc2)cc1)CCCCCC	-9.12757
21	CHEMBL3827917	O=C(O)[C@@H](NC(=O)COc1c(OC)cc(/C=C/C(=O)CC(=O)/C=C/c2cc(OC)c(O)cc2)cc1)CCCCN	-8.92006
22	CHEMBL3759749	O=C(/C=C/c1cc(OC)c(OC/C=C(\CC/C=C(\CC/C=C(\C)/C)/C)/C)cc1)CC(=O)/C=C/c1cc(OC)c(OC/C=C(\CC/C=C(\CC/C=C(\C)/C)/C)/C)cc1	-9.45939
23	CHEMBL1077099	O=C(/C=C/c1cc(OC)c(OC(=O)c2ccccc2)cc1)CC(=O)/C=C/c1cc(OC)c(OC(=O)c2ccccc2)cc1	-9.06118
24	CHEMBL4098212	O=C(Oc1c(OC)cc(/C=C/C(=O)C(C(=O)/C=C/c2cc(OC)c(OC(=O)C(CO)(CO)C)cc2)(CC)CC)cc1)C(CO)(CO)C	-9.75226
25	CHEMBL2064002	Brc1c(O)c(OC)cc(/C=C(\C(=O)/C=C/c2c(OC)ccc(OC)c2)/C(=O)/C=C/c2c(OC)ccc(OC)c2)c1	-8.91475
26	CHEMBL2064106	Fc1c(O)ccc(/C=C(\C(=O)/C=C/c2c(OC)cc(OC)cc2OC)/C(=O)/C=C/c2c(OC)cc(OC)cc2OC)c1	-9.81097
27	CHEMBL3598007	Clc1c(Nc2c(CC(=O)Oc3c(OC)cc(/C=C/C(=O)CC(=O)/C=C/c4cc(OC)c(OC(=O)Cc5c(Nc6c(Cl)cccc6Cl)cccc5)cc4)cc3)cccc2)c(Cl)ccc1	-10.7538
28	CHEMBL3598002	O=C(Oc1c(OC)cc(/C=C/C(=O)CC(=O)/C=C/c2cc(OC)c(O)cc2)cc1)CCc1oc(c(-c2ccccc2)n1)-c1ccccc1	-9.44806
29	CHEMBL3827366	O=C(O)[C@@H](NC(=O)COc1c(OC)cc(/C=C/C(=O)CC(=O)/C=C/c2cc(OC)c(O)cc2)cc1)CCCCNC(=O)[C@@H](NC(=O)[C@@H](NC(=O)[C@@H]1CNCC1)C)CCCCN	-10.3758
30	CHEMBL4299939	O=C(/C=C/c1cc(OCc2ccccc2)c(OCCCN)cc1)CC(=O)/C=C/c1cc(OCc2ccccc2)c(OCCCN)cc1	-9.2898
31	CHEMBL2260082	ClCCN(CC(=O)Oc1c(OCC)cc(/C=C/C(=O)CC(=O)/C=C/c2cc(OCC)c(OC(=O)CN(CCCl)CCCl)cc2)cc1)CCCl	-9.56722
32	CHEMBL2064000	O=C(/C(/C(=O)/C=C/c1c(OC)ccc(OC)c1)=C\c1cc(OC)c(OC)cc1)/C=C/c1c(OC)ccc(OC)c1	-9.47049
33	CHEMBL2260092	O=C(Oc1c(OC)cc(/C=C/C(=O)CC(=O)/C=C/c2cc(OC)c(OC(=O)CNCCN(C)C)cc2)cc1)CNCCN(C)C	-10.2721
34	CHEMBL2260072	O=C(/C=C/c1cc(OCC)c(OC(=O)c2sccc2)cc1)CC(=O)/C=C/c1cc(OCC)c(OC(=O)c2sccc2)cc1	-9.70894
35	CHEMBL2260079	O=C(Oc1c(OCC)cc(/C=C/C(=O)CC(=O)/C=C/c2cc(OCC)c(OC(=O)CNc3sc4c(n3)ccc(OC)c4)cc2)cc1)CNc1sc2c(n1)ccc(OC)c2	-9.6858
36	CHEMBL2063994	O=C(/C(/C(=O)/C=C/c1c(OC)c(OC)ccc1)=C\c1c(OC)c(OC)ccc1)/C=C/c1c(OC)c(OC)ccc1	-8.89896

37	CHEMBL2260077	ClCCNCCC(=O)Oc1c(OC)cc(/C=C/C(=O)CC(=O)/C=C/c2cc(OC)c(OC(=O)CCNCCCl)cc2)cc1	-9.43085
38	CHEMBL3597997	FC(F)(F)c1cc(Nc2c(C(=O)Oc3c(OC)cc(/C=C/C(=O)CC(=O)/C=C/c4cc(OC)c(O)cc4)cc3)cccc2)ccc1	-9.28529
39	CHEMBL2260089	O=C(Oc1c(OC)cc(/C=C/C(=O)CC(=O)/C=C/c2cc(OC)c(OC(=O)CNc3sc(C)nn3)cc2)cc1)CNc1sc(C)nn1	-10.1299
40	CHEMBL2259875	O=C(Oc1c(OCC)cc(/C=C/C(=O)CC(=O)/C=C/c2cc(OCC)c(OC(=O)CCNCCN(CC)CC)cc2)cc1)CCNCCN(CC)CC	-9.80431
41	CHEMBL212610	O=C(/C(/C(=O)/C=C/c1cc(OC)c(OC2OCCCC2)cc1)=C\C=C(/OCC)\O)/C=C/c1cc(OC)c(OC2OCCCC2)cc1	-10.2018
42	CHEMBL508738	O=C(/C=C/c1ccc(O[C@H]2[C@H](O)[C@@H](O)[C@H](O)[C@@H](CO)O2)cc1)CC(=O)/C=C/c1ccc(O[C@H]2[C@H](O)[C@@H](O)[C@H](O)[C@@H](CO)O2)cc1	-9.16657
43	CHEMBL4537307	O=C(/C=C/c1cc(OC)c(O[C@H]2[C@H](O)[C@@H](O)[C@H](O)[C@@H](CO)O2)cc1)CC(=O)/C=C/c1cc(OC)c(O[C@H]2[C@H](O)[C@@H](O)[C@H](O)[C@@H](CO)O2)cc1	-9.68433
44	CHEMBL3598008	Fc1c(-c2cc(O)c(C(=O)Oc3c(OC)cc(/C=C/C(=O)CC(=O)/C=C/c4cc(OC)c(OC(=O)c5c(O)cc(-c6c(F)cc(F)cc6)cc5)cc4)cc3)cc2)ccc(F)c1	-9.94333
45	CHEMBL3598010	FC(F)(F)c1cc(Nc2c(C(=O)Oc3c(OC)cc(/C=C/C(=O)CC(=O)/C=C/c4cc(OC)c(OC(=O)c5c(Nc6cc(C(F)(F)F)ccc6)cccc5)cc4)cc3)cccc2)ccc1	-10.6145
46	CHEMBL2260088	O=C(Oc1c(OC)cc(/C=C/C(=O)CC(=O)/C=C/c2cc(OC)c(OC(=O)CNc3sc4c(n3)ccc(OC)c4)cc2)cc1)CNc1sc2c(n1)ccc(OC)c2	-10.033
47	CHEMBL2260080	O=C(Oc1c(OCC)cc(/C=C/C(=O)CC(=O)/C=C/c2cc(OCC)c(OC(=O)CNc3sc(C)nn3)cc2)cc1)CNc1sc(C)nn1	-9.56871
48	CHEMBL2064108	O=C(/C(/C(=O)/C=C/c1c(OC)cc(OC)cc1OC)=C\c1cc(OC)c(O)c(OC)c1)/C=C/c1c(OC)cc(OC)cc1OC	-9.50885
49	CHEMBL3758656	O=C(/C=C/c1cc(OC)c(OC/C=C(\CC/C=C(\C)/C)/C)cc1)C(C(=O)/C=C/c1cc(OC)c(OC/C=C(\CC/C=C(\C)/C)/C)cc1)C/C=C(\CC/C=C(\C)/C)/C	-11.5176
50	CHEMBL1800975	O=C(/C(/C(=O)/C=C/c1cc(OC)c(OC)c(OC)c1)=C\c1cc(OC)c(OC)cc1)/C=C/c1cc(OC)c(OC)c(OC)c1	-9.37018
51	CHEMBL3746241	O=C(/C(/C(=O)/C=C/c1cc(OC)c(O)cc1)=C\c1cc2C(=O)N(C(=O)c2cc1)C1C(=O)NC(=O)CC1)/C=C/c1cc(OC)c(O)cc1	-9.18674
52	CHEMBL3598014	O=C(/C=C/c1cc(OC)c(OC(=O)c2c(Nc3c(C)c(C)ccc3)cccc2)cc1)CC(=O)/C=C/c1cc(OC)c(OC(=O)c2c(Nc3c(C)c(C)ccc3)cccc2)cc1	-10.0827
53	CHEMBL455159	O=C(Oc1c(OC)cc(/C=C/C(=O)CC(=O)/C=C/c2cc(OC)c(OC(=O)[C@@H](N)C(C)C)cc2)cc1)[C@@H](N)C(C)C	-8.86642
54	CHEMBL3827698	O=C(O)[C@@H](NC(=O)COc1c(OC)cc(/C=C/C(=O)CC(=O)/C=C/c2cc(OC)c(OC)cc2)cc1)CCCCN	-8.87809
55	CHEMBL3759699	O=C(C(C(=O)/C=C/c1cc(OC)c(OC/C=C(\CC/C=C(\C)/C)/C)cc1)(C/C=C(\CC/C=C(\C)/C)/C)C/C=C(\CC/C=C(\C)/C)/C)/C=C/c1cc(OC)c(OC/C=C(\CC/C=C(\C)/C)/C)cc1	-10.9522

56	CHEMBL3759699	O=C(/C=C/c1cc(C(C)(C)C)c(O)c(C(C)(C)C)c1)CC(=O)/C=C/c1cc(C(C)(C)C)c(O)c(C(C)(C)C)c1	-9.19664
57	CHEMBL2259872	O=C(Oc1c(OCC)cc(/C=C/C(=O)CC(=O)/C=C/c2cc(OCC)c(OC(=O)CNCCN(CC)CC)cc2)cc1)CNCCN(CC)CC	-11.4745
58	CHEMBL3558372	O=C(/C=C/c1cc(OC)c(OCCCCC[n+]2ccccc2)cc1)CC(=O)/C=C/c1cc(OC)c(OCCCCC[n+]2ccccc2)cc1	-10.0854
59	CHEMBL3598011	O=C(Oc1c(OC)cc(/C=C/C(=O)CC(=O)/C=C/c2cc(OC)c(OC(=O)C(C)c3ccc(CC(C)C)cc3)cc2)cc1)C(C)c1ccc(CC(C)C)cc1	-9.70746
60	CHEMBL4444656	O=C(/C(/C(=O)/C=C/c1cc(OC)c(O)cc1)=C\C1CCN(C(OC(C)(C)C)=O)CC1)/C=C/c1cc(OC)c(O)cc1	-8.88335
61	CHEMBL3765149	O=C(/C=C/c1cc(OC)c(OCCN2CCN(Cc3ccc(-c4oc(-c5cc(Nc6nc(-c7cnccc7)ccn6)c(C)cc5)nn4)cc3)CC2)cc1)CC(=O)/C=C/c1cc(OC)c(O)cc1	-10.1332
62	CHEMBL2063997	O=C(/C(/C(=O)/C=C/c1c(OC)c(OC)ccc1)=C\c1c(OC)c(OC)c(OC)cc1)/C=C/c1c(OC)c(OC)ccc1	-9.27672
63	CHEMBL4300045	O=C(/C=C/c1cc(OCCN)c(OCCN)c(OCCN)c1)CC(=O)/C=C/c1cc(OCCN)c(OCCN)c(OCCN)c1	-9.54523
64	CHEMBL3598019	Clc1c(C)c(Nc2c(C(=O)Oc3c(OC)cc(/C=C/C(=O)CC(=O)/C=C/c4cc(OC)c(OC(=O)c5c(Nc6c(C)c(Cl)ccc6)cccc5)cc4)cc3)cccc2)ccc1	-9.40117
65	CHEMBL1077036	O=C(O)C(NC(=O)c1ccc(NCc2nc3C(=O)NC(N)=Nc3nc2)cc1)CCC(=O)OCCOc1c(OC)cc(/C=C/C(=O)CC(=O)/C=C/c2cc(OC)c(O)cc2)cc1	-9.39491
66	CHEMBL2397422	O=[N+]([O-])c1cc(-c2c(/C=C(\C(=O)/C=C/c3cc(OC)c(O)cc3)/C(=O)/C=C/c3cc(OC)c(O)cc3)cn(-c3ccccc3)n2)ccc1	-9.1872
67	CHEMBL1800964	O=C(/C(/C(=O)/C=C/c1cc(OC)c(OC)cc1)=C\c1cc(OC)c(OC)c(OC)c1)/C=C/c1cc(OC)c(OC)cc1	-9.27477
68	CHEMBL3827416	O=C(O)[C@@H](NC(=O)COc1c(OC)cc(/C=C/C(=O)CC(=O)/C=C/c2cc(OC)c(OC)cc2)cc1)CCCCNC(=O)[C@@H](NC(=O)[C@@H](NC(=O)[C@@H]1CNCC1)C)CCCCN	-10.2491
69	CHEMBL541222	O=C(/C(/C(=O)/C=C/C=1C(C)(C)CCCC=1C)=C\c1cc(OCC)c(OCC)cc1)/C=C/c1cc(OCC)c(OCC)cc1	-9.44678
70	CHEMBL2260091	ClCCN(CC(=O)Oc1c(OC)cc(/C=C/C(=O)CC(=O)/C=C/c2cc(OC)c(OC(=O)CN(CCCl)CCCl)cc2)cc1)CCCl	-9.07722
71	CHEMBL3598012	Clc1ccc(C(=O)n2c(C)c(CC(=O)Oc3c(OC)cc(/C=C/C(=O)CC(=O)/C=C/c4cc(OC)c(OC(=O)Cc5c(C)n(C(=O)c6ccc(Cl)cc6)c6c5cc(OC)cc6)cc4)cc3)c3c2ccc(OC)c3)cc1	-9.14394
72	CHEMBL2397432	O=C(/C(/C(=O)/C=C/c1cc(OC)c(O)cc1)=C\c1c(-c2c(O)cccc2)nn(-c2ccccc2)c1)/C=C/c1cc(OC)c(O)cc1	-9.26866
73	CHEMBL3598006	O=C(/C=C/c1cc(OC)c(OC(=O)c2c(OC(=O)C)cccc2)cc1)CC(=O)/C=C/c1cc(OC)c(OC(=O)c2c(OC(=O)C)cccc2)cc1	-9.25243
74	CHEMBL2063996	Brc1c(O)c(OC)cc(/C=C(\C(=O)/C=C/c2c(OC)c(OC)ccc2)/C(=O)/C=C/c2c(OC)c(OC)ccc2)c1	-9.08231

75	CHEMBL558067	O=C(/C(/C(=O)/C=C/C=1C(C)(C)CCCC=1C)=C\c1cc(OC)c(O)c(OC)c1)/C=C/c1cc(OC)c(O)c(OC)c1	-9.28434
76	CHEMBL3598009	O=C(Oc1c(OC)cc(/C=C/C(=O)CC(=O)/C=C/c2cc(OC)c(OC(=O)CC3(CC)OCCc4c5c(c(CC)ccc5)[nH]c34)cc2)cc1)CC1(CC)OCCc2c3c(c(CC)ccc3)[nH]c12	-9.66999
77	CHEMBL4088696	O=C(Oc1c(OC)cc(/C=C/C(=O)C(C(=O)/C=C/c2cc(OC)c(OC(=O)C(CO)(CO)C)cc2)(C)C)cc1)C(CO)(CO)C	-9.10242
78	CHEMBL444980	O=C(Oc1c(OC)cc(/C=C/C(=O)CC(=O)/C=C/c2cc(OC)c(OC(=O)[C@@H](N)CCC(=O)O)cc2)cc1)[C@@H](N)CCC(=O)O	-10.2566
79	CHEMBL4087450	O=C(/C(/C(=O)/C=C/c1cc(OCC)c(O)cc1)=C\c1cc(OCC)c(O)cc1)/C=C/c1cc(OCC)c(O)cc1	-9.12395
80	CHEMBL3667025	FC(F)(F)Oc1c(O)ccc(/C=C/C(=O)C(C(=O)/C=C/c2cc(OC(F)(F)F)c(O)cc2)CCC(=O)OC(C)(C)C)c1	-8.91507
81	CHEMBL1800974	O=C(/C(/C(=O)/C=C/c1cc(OC)c(OC)cc1)=C\c1c(OC)c(OC)ccc1)/C=C/c1cc(OC)c(OC)cc1	-9.37714
82	CHEMBL2259876	S(=O)(=O)(N)c1ccc(NCC(=O)Oc2c(OC)cc(/C=C/C(=O)CC(=O)/C=C/c3cc(OC)c(OC(=O)CNc4ccc(S(=O)(=O)N)cc4)cc3)cc2)cc1	-10.2654
83	CHEMBL1091724	O=C(Oc1c(OC)cc(/C=C/C(=O)CC(=O)/C=C/c2cc(OC)c(OC(=O)[C@@H](N)CC(=O)O)cc2)cc1)[C@@H](N)CC(=O)O	-8.97903
84	CHEMBL3758432	O=C(C(C(=O)/C=C/c1cc(OC)c(OC/C=C(\C)/C)cc1)(C/C=C(\C)/C)C/C=C(\C)/C)/C=C/c1cc(OC)c(OC/C=C(\C)/C)cc1	-9.49989
85	CHEMBL1253958	O=C(/C=C/c1cc(OC)c(OCCCCC)cc1)CC(=O)/C=C/c1cc(OC)c(OCCCCC)cc1	-9.59552
86	CHEMBL4079269	O=C(Oc1c(OC)cc(/C=C/C(=O)C(C#CCO)(C#CCO)C(=O)/C=C/c2cc(OC)c(OC(=O)C(CO)(CO)C)cc2)cc1)C(CO)(CO)C	-9.74854
87	CHEMBL4640876	O=C(O[C@H]1[C@H](C)[C@H]2[C@@]34OO[C@@](C)(O[C@H]3O1)CC[C@H]4[C@H](C)CC2)CCC(=O)Oc1c(OC)cc(/C=C/C(=O)CC(=O)/C=C/c2cc(OC)c(O)cc2)cc1	-9.49036
88	CHEMBL2259874	O=C(Oc1c(OCC)cc(/C=C/C(=O)CC(=O)/C=C/c2cc(OCC)c(OC(=O)CCNCCN(C)C)cc2)cc1)CCNCCN(C)C	-10.0948
89	CHEMBL4639879	O=C(C(C(=O)/C=C/c1cc(OC)c(OCC(=O)OC)cc1)(CC(=O)OC)CC(=O)OC)/C=C/c1cc(OC)c(OCC(=O)OC)cc1	-9.06458
90	CHEMBL4165877	O=C(/C=C/c1cc(OC)c(OCc2c(C)nc(C)c(C)n2)cc1)CC(=O)/C=C/c1cc(OC)c(OCc2c(C)nc(C)c(C)n2)cc1	-9.05718
91	CHEMBL2392187	O=C(/C=C/c1cc(OC)c(O)c(C/C=C(\C)/C)c1)CC(=O)/C=C/c1cc(OC)c(O)c(C/C=C(\C)/C)c1	-9.33403
92	CHEMBL2260073	O=C(Oc1c(OC)cc(/C=C/C(=O)CC(=O)/C=C/c2cc(OC)c(OC(=O)CNCCN(CC)CC)cc2)cc1)CNCCN(CC)CC	-9.39647
93	CHEMBL2260075	O=C(Oc1c(OC)cc(/C=C/C(=O)CC(=O)/C=C/c2cc(OC)c(OC(=O)CCNc3sc4c(n3)ccc(OC)c4)cc2)cc1)CCNc1sc2c(n1)ccc(OC)c2	-9.42579

94	CHEMBL1800976	O=C(/C(/C(=O)/C=C/c1cc(OC)c(OC)cc1)=C\c1c(OC)ccc(OC)c1)/C=C/c1cc(OC)c(OC)cc1	-8.938
95	CHEMBL211812	FC(C(=O)/C=C/c1cc(OC)c(OC2OCCCC2)cc1)(C(=O)/C=C/c1cc(OC)c(OC2OCCCC2)cc1)CCC(=O)OCC	-9.82298
96	CHEMBL448824	O=C(Oc1c(OC)cc(/C=C/C(=O)CC(=O)/C=C/c2cc(OC)c(OC(=O)/C=C/C=C/c3cc(O)c(O)cc3)cc2)cc1)/C=C/C=C/c1cc(O)c(O)cc1	-9.24333
97	CHEMBL3597995	Fc1c(-c2cc(O)c(C(=O)Oc3c(OC)cc(/C=C/C(=O)CC(=O)/C=C/c4cc(OC)c(O)cc4)cc3)cc2)ccc(F)c1	-9.50089
98	CHEMBL473041	O=C(/C=C/c1cc(OC)c(O)c(C(/C=C/c2cc(OC)c(OC)cc2)C)c1)CC(=O)/C=C/c1cc(OC)c(O)cc1	-8.94995
99	CHEMBL450047	O=C(/C=C/c1cc(OC)c(O)c(C(/C=C/c2c(OC)cc(OC)c(OC)c2)C)c1)CC(=O)/C=C/c1cc(OC)c(O)cc1	-9.37303
100	CHEMBL378846	O=C(/C(/C(=O)/C=C/c1cc(OC)c(O)cc1)=C\C=C(/OCC)\O)/C=C/c1cc(OC)c(O)cc1	-9.11455

Supplementary table 3. Selected RMSD, RMSF, Rg, and SASA value of Lapatinib and Curcumin Derivatives in complex with HER2 L755S, HER2 T798I, and HER2 T798M

HER2 L755S*				
Compound	RMSD**	RMSF***	Rg**	SASA**
HER2 L755S	2.5963 ± 0.5146	2.5421 ± 0.5272	31.1379 ± 0.4037	397.5591 ± 31.4130
HER2 L755S+CHEMBL3758656	3.7323 ± 0.5287	1.7435 ± 0.6670	31.8764 ± 0.3236	377.8879 ± 28.0848
HER2 L755S+CHEMBL3827366	2.7127 ± 0.4358	2.2223 ± 0.6418	31.8068 ± 0.2527	338.5619 ± 18.0597
HER2 L755S+Lapatinib	3.8734 ± 0.5335	1.7489 ± 0.6290	31.9469 ± 0.3813	424.0151 ± 46.6045
HER2 T798I*				
Compound	RMSD**	RMSF***	Rg**	SASA**
HER2 T798I	3.5471 ± 0.6026	1.9927 ± 0.5325	31.5545 ± 0.2847	370.3534 ± 31.0325
HER2 T798I+CHEMBL3758656	4.3060 ± 0.6329	1.2665 ± 0.6417	30.8208 ± 0.2307	344.0984 ± 23.4107
HER2 T798I+CHEMBL3827366	4.8675 ± 0.9201	1.9309 ± 0.4972	32.4446 ± 0.4332	366.7163 ± 21.3381
HER2 T798I+Lapatinib	3.8610 ± 0.5799	1.6654 ± 0.6493	31.2615 ± 0.2359	385.9371 ± 27.9364
HER2 T798M*				
Compound	RMSD**	RMSF***	Rg**	SASA**
HER2 T798M	2.9399 ± 0.5049	1.5769 ± 0.4454	31.7918 ± 0.2452	317.3796 ± 25.2859
HER2 T798M+CHEMBL3758656	4.2836 ± 0.4868	2.2466 ± 0.4751	31.9298 ± 0.2576	385.2948 ± 29.9294
HER2 T798M+CHEMBL3827366	3.8428 ± 0.5072	1.6453 ± 0.5231	32.5311 ± 0.3189	428.1876 ± 40.0778
HER2 T798M+Lapatinib	4.8196 ± 0.5435	1.6654 ± 0.6494	31.2615 ± 0.2359	385.9371 ± 27.9364

*Data was expressed in mean ± SD

**The value was obtained at 10 ns simulation

***The value was obtained at amino acid Val750

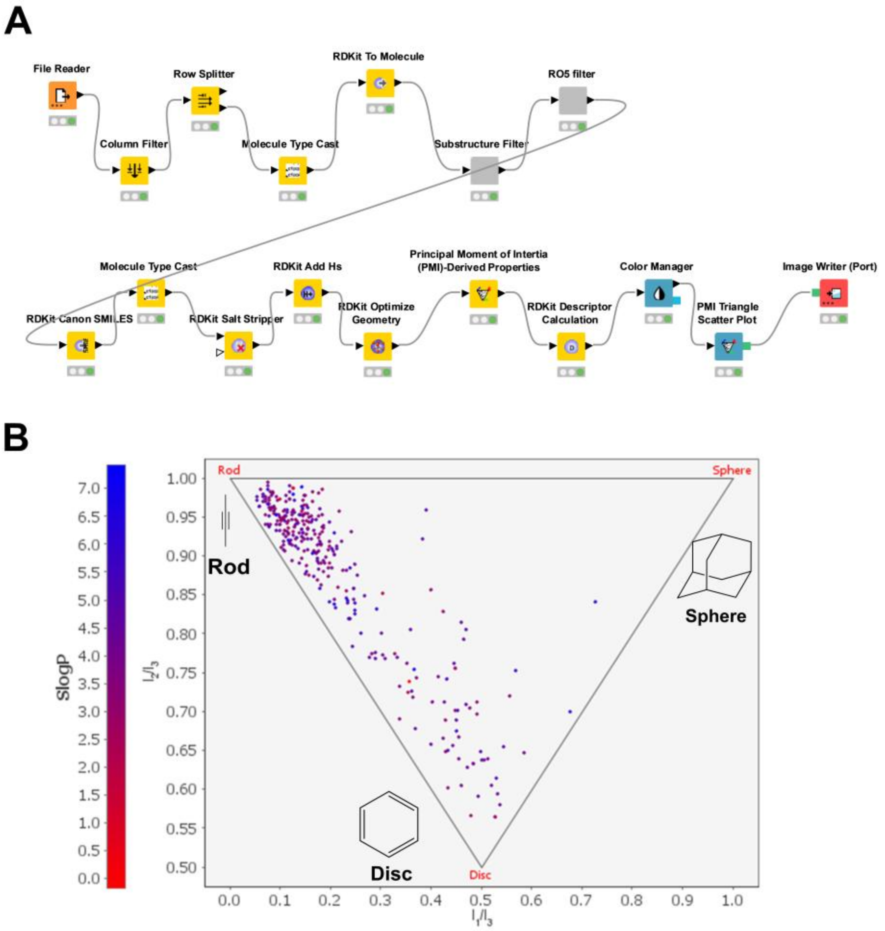

Supplementary Figure 1. Curation and analysis of curcumin derivatives database. (A) KNIME workflow for filtering curcumin derivatives. (B) PMI analysis of retrieved curcumin derivatives from the database

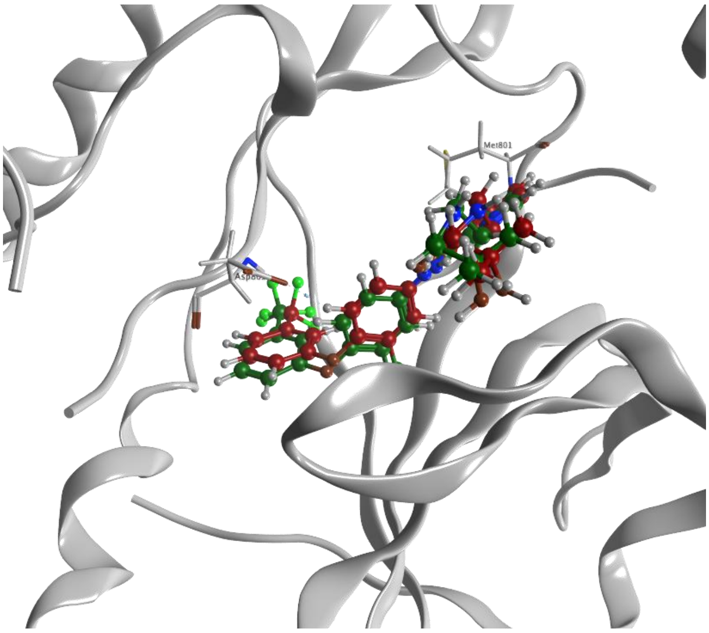

Supplementary Figure 2. The crystal structure of the redocking study used TAK-285 as the native ligand. Protein was illustrated as a grey ribbon while compounds and interacting amino acids were demonstrated as ball stick color.

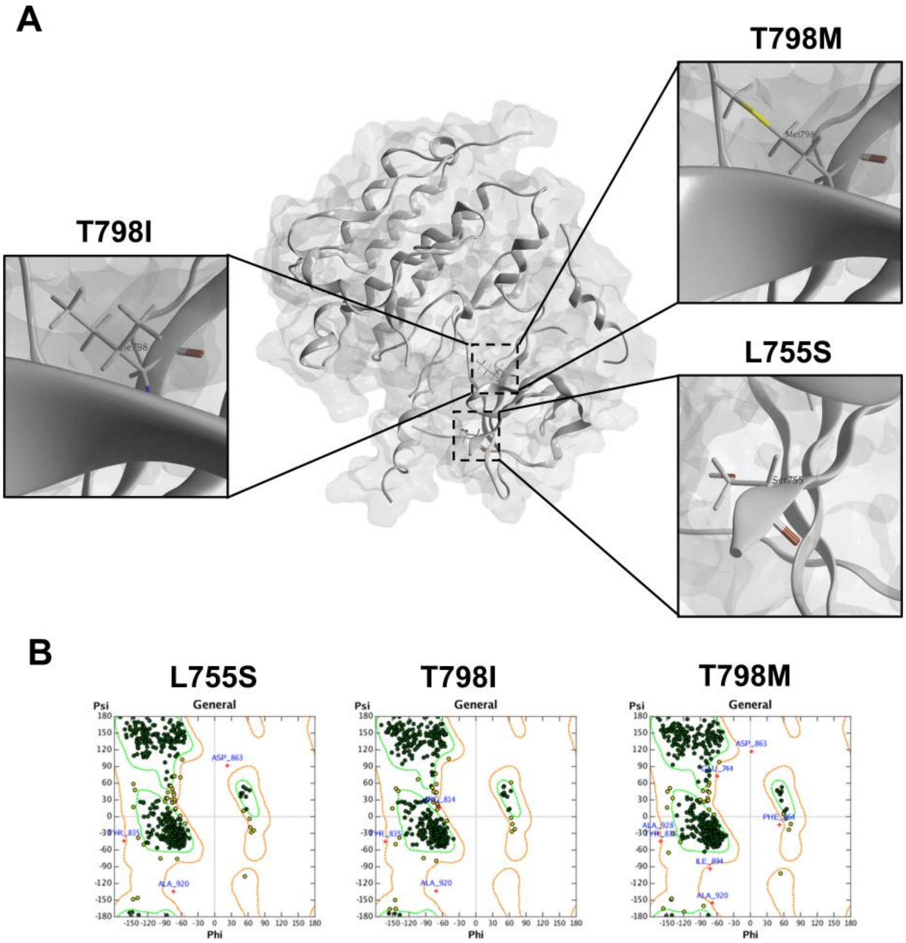

Supplementary Figure 3. Structural and conformational analysis of HER2 mutations (L755S, T798I, and T798M) and their Ramachandran plot. (A) Overal structure of kinase domain highlighting the mutation site. (B) Ramachandran plots for mutated residues. The green dots represent the conformational spaces of the Phi-Psi angles for mutated residues. Deviations from typically allowed regions (orange outlines) suggest changes in backbone conformational preferences.

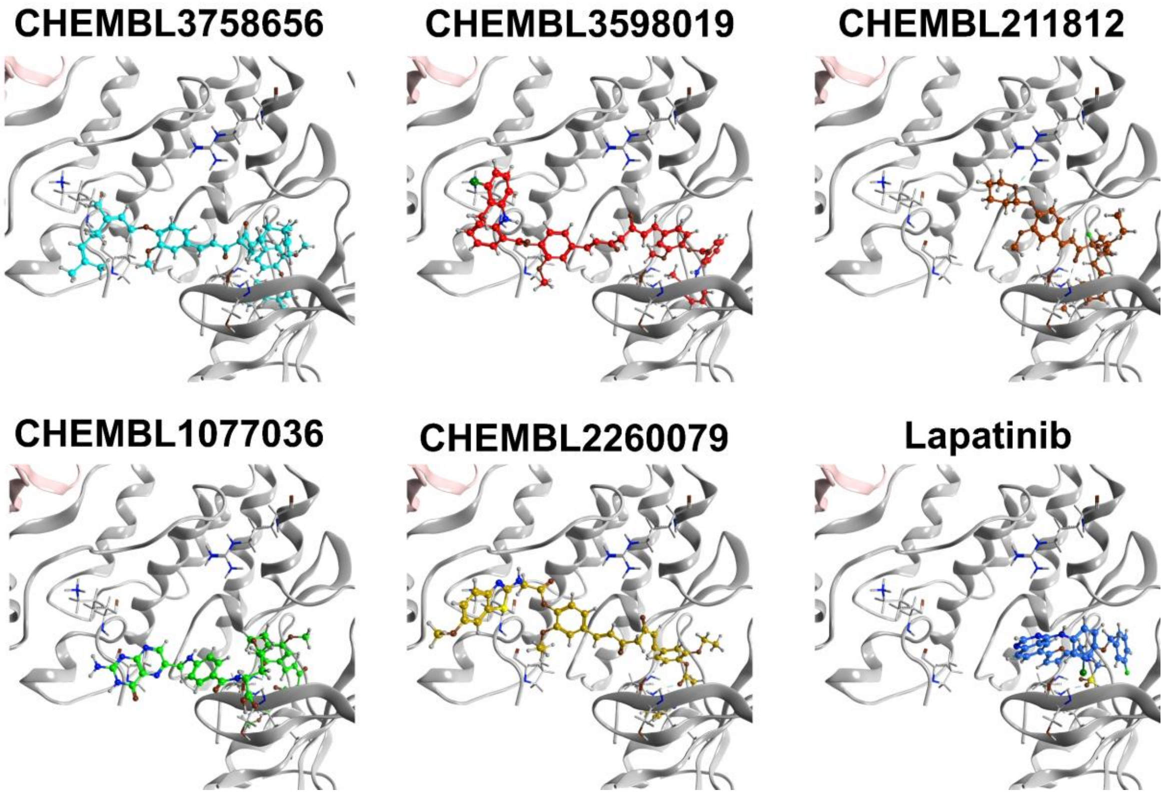

Supplementary Figure 4. Binding interaction of Lapatinib and top five Curcumin Derivatives against HER2 L755S in 3D Visualization

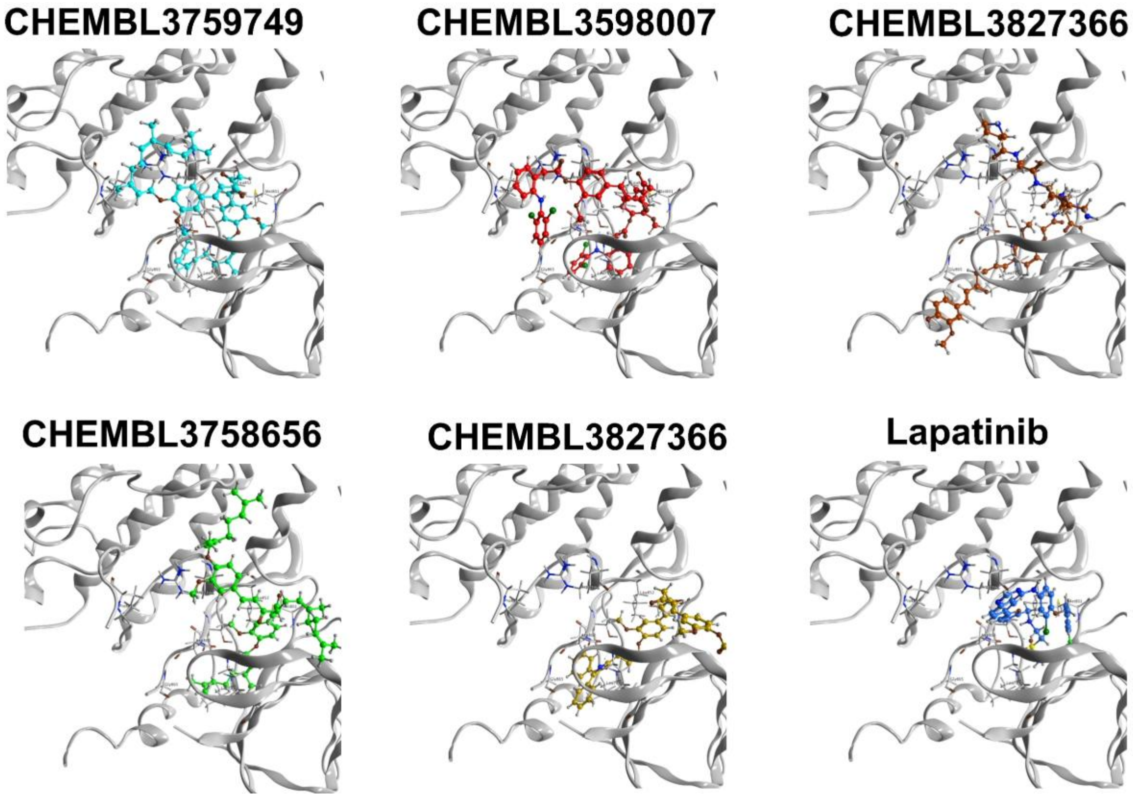

Supplementary Figure 5. Binding interaction of Lapatinib and top five Curcumin Derivatives against HER2 T798I in 3D Visualization

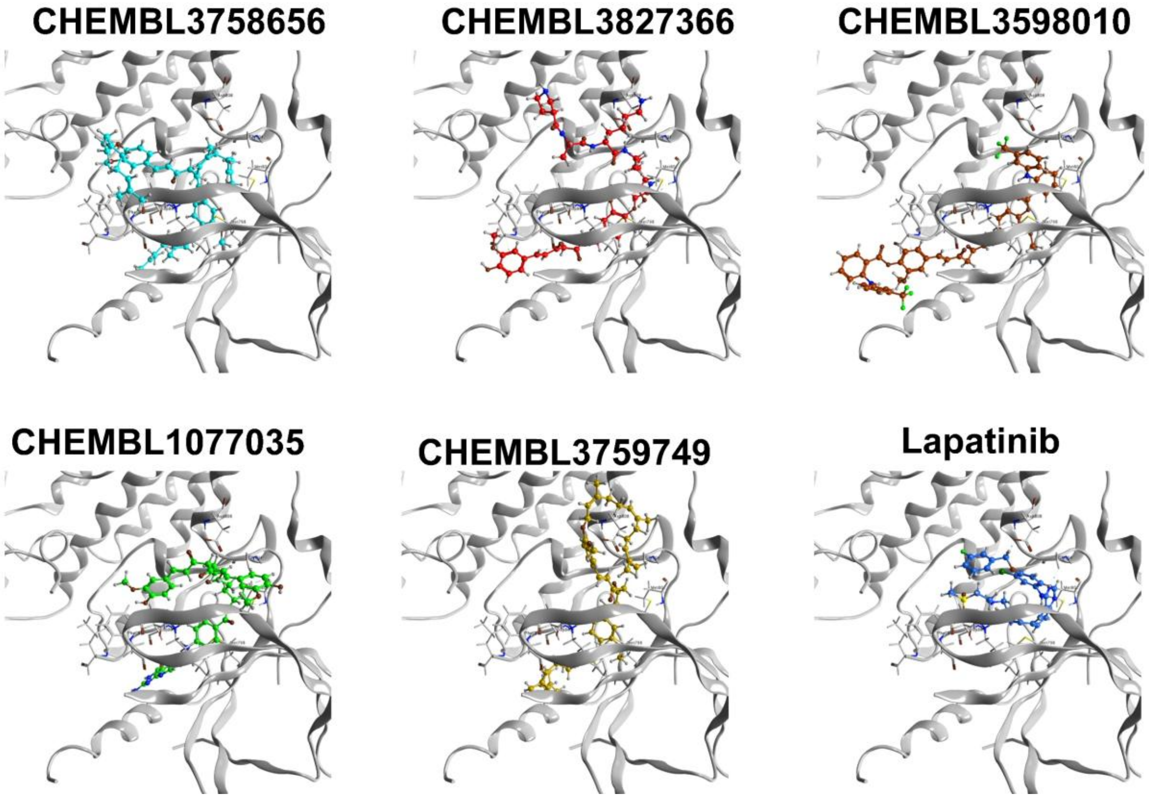

Supplementary Figure 6. Binding interaction of Lapatinib and top five Curcumin Derivatives against HER2T798M in 3D Visualization

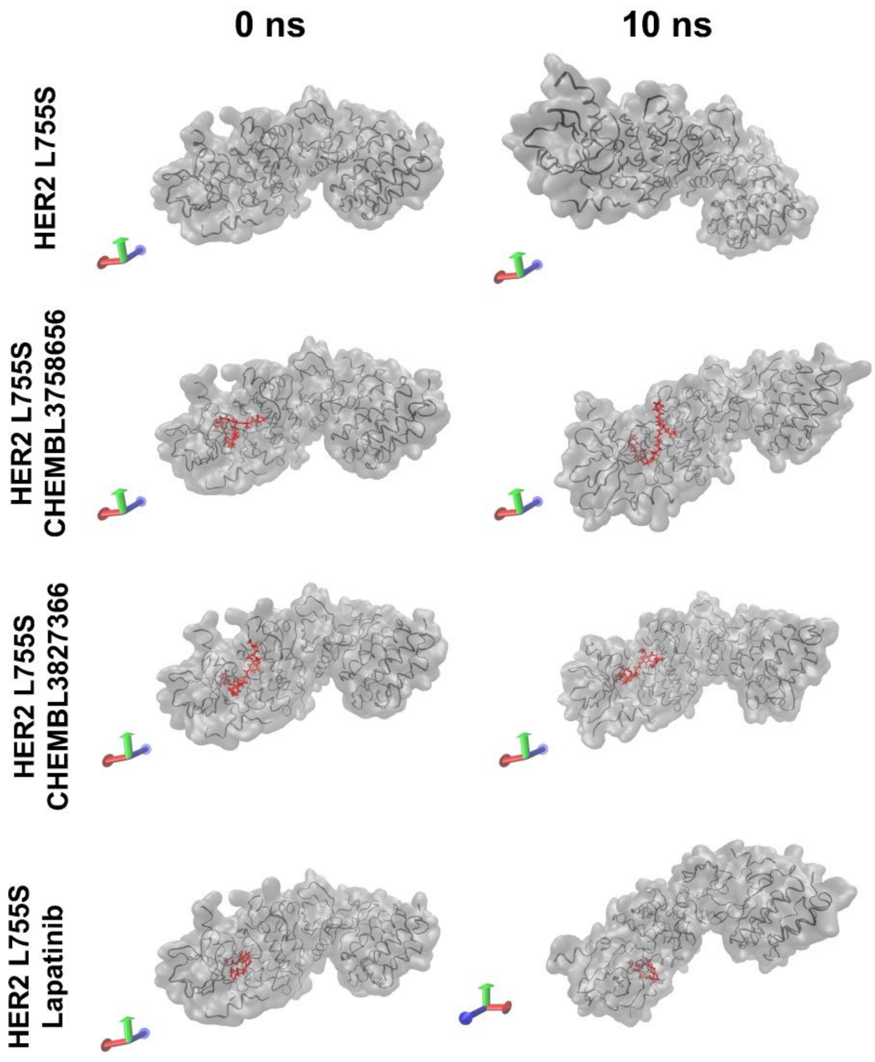

Supplementary Figure 7. Visualization of the binding interaction of curcumin derivatives againts HER2 L755S

Commented [R1]: why 10 ns

Commented [r2R1]: Thank you very much for your concern, the main reason for performing 10 ns simulation is sufficient and adequate to gain preliminary insights into the binding stability and dynamic behavior of curcumin derivatives complexed with HER2 mutants. Several reports below performed short simulation on their work,

Hermawan, A., Wulandari, F., Hanif, N., Utomo, R. Y., Jenie, R. I., Ikawati, M., & Tafrihani, A. S. (2022). Identification of potential targets of the curcumin analog CCA-1.1 for glioblastoma treatment: integrated computational analysis and in vitro study. Scientific Reports, 12(1), 13928.
Lestari B, Utomo RY, Rahman FA, Putri DDP, Zulfin UM, Suenaga Y, Meiyanto E, Hippo Y. Discovery of pyroptosis-inducing natural products in neuroblastomas: computational studies with experimental validation. BMC Complement Med Ther.25, 1,279

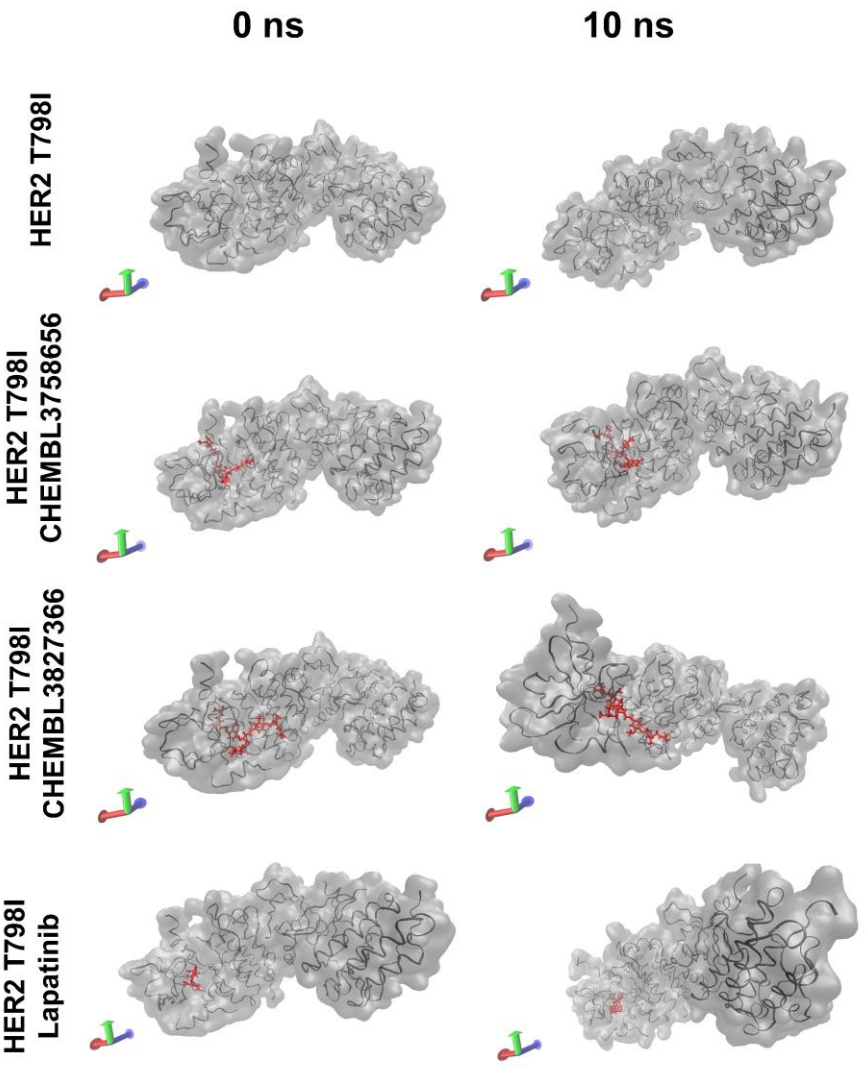

Supplementary Figure 8. Visualization of the binding interaction of curcumin derivatives againts HER2 T798I.

Commented [R3]: Images are useful but would benefit from structural annotations (e.g., ligand conformation changes).

Commented [r4R3]: Thank you very much for the suggestion, we have added the explanation of structural change in the discussionsection

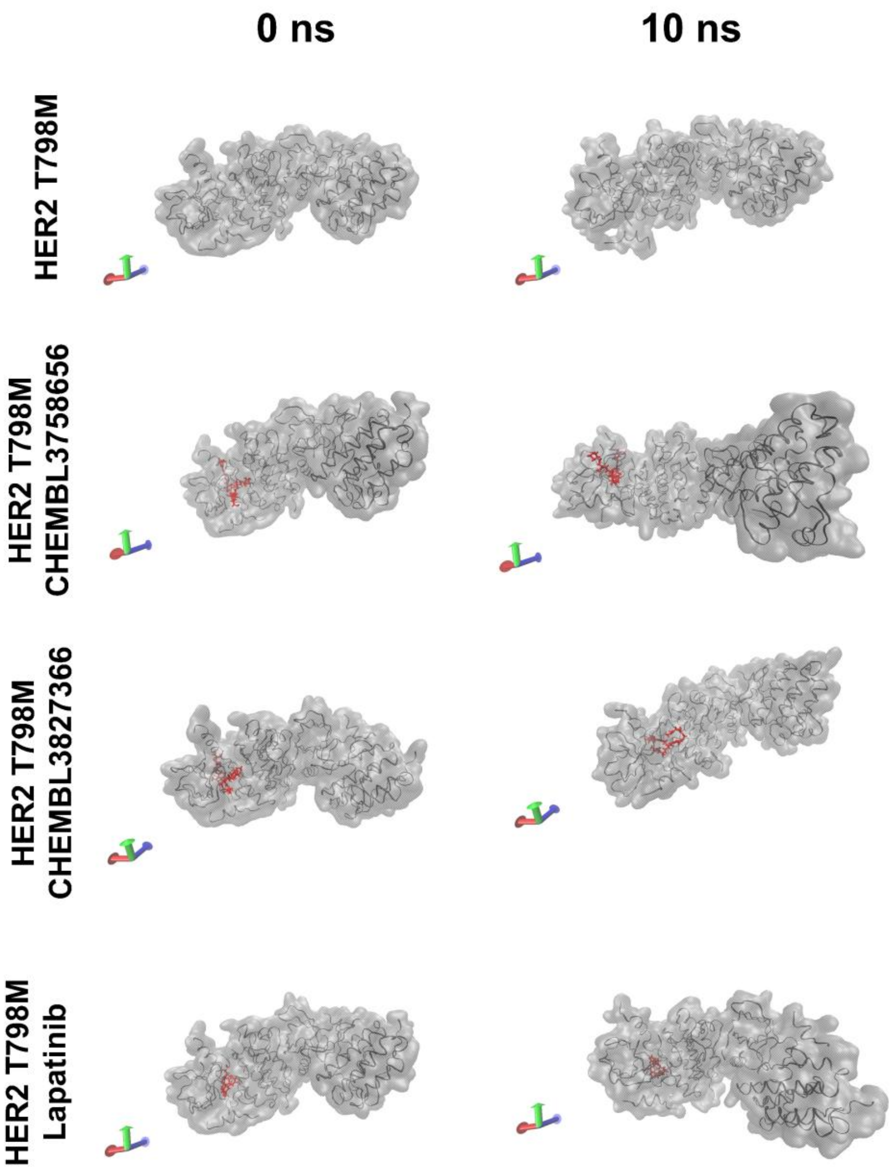

Supplementary Figure 9. Visualization of the binding interaction of curcumin derivatives againts HER2 T798M.
